# Evaluation of a multi-pronged intervention to improve access to safe abortion care in two districts in Jharkhand

**DOI:** 10.1186/1472-6963-14-227

**Published:** 2014-05-21

**Authors:** Sushanta K Banerjee, Kathryn L Andersen, Traci L Baird, Bela Ganatra, Sangeeta Batra, Janardan Warvadekar

**Affiliations:** 1 Ipas India, E-63 Vasant Marg, Vasant Vihar, New Delhi, India; 2 Ipas, 300 Market St., Suite 200, Chapel Hill, NC 27516, USA; 3 Department of Reproductive Health and Research, World Health Organization, Geneva, Switzerland

**Keywords:** Medical abortion, Community health workers, India, Evaluation

## Abstract

**Background:**

Despite the adoption of the Medical Termination of Pregnancy Act in 1972, access to safe abortion services remains limited in India. Awareness of the legality of abortion also remains low, leading many women to seek services outside the health system. Medical abortion (MA) is an option that has the potential to expand access to safe abortion services. A multi-pronged intervention covering a population of 161,000 in 253 villages in the Silli and Khunti blocks of Jharkhand was conducted between 2007 and 2009, seeking to improve medical abortion services and create awareness at the community level by providing information through community intermediaries and creating an enabling environment through a behavior change communication campaign. The study evaluates the changes in knowledge about abortion-related issues, changes in abortion care-seeking, and service utilization as a result of this intervention.

**Methods:**

A baseline cross-sectional survey was conducted pre-intervention (n = 1,253) followed by an endline survey (n = 1,290) one year after the completion of the intervention phase. In addition, monitoring data from intervention facilities was collected monthly over the study period.

**Results:**

Nearly 85% of respondents reported being exposed to safe abortion messaging as a result of the intervention. Awareness of the legality of abortion increased significantly from 19.7% to 57.6% for women, as did awareness of the specific conditions for which abortion is allowed. Results were similar for men. There was also a significant increase in the proportion of men and women who knew of a legal and safe provider and place from where abortion services could be obtained. Multivariate analysis showed positive associations between exposure to any component of the intervention and increased knowledge about legality and gestational age limits, however only interpersonal communication was associated with a significant increase in knowledge of where to obtain safe services (OR 4.8, SE 0.67). Utilization of safe abortion services, and in particular MA, increased at all intervention sites over the duration of the intervention with a shift towards women seeking care earlier in pregnancy.

**Conclusion:**

The evaluation demonstrates the success of the intervention and its potential for replication in similar contexts within India.

## Background

Despite adoption of the Medical Termination of Pregnancy (MTP) Act that has allowed for abortion on wide-ranging medical and social grounds since 1972, access to safe abortion services remains limited in India. Abortion-related deaths contribute to an estimated 8% of maternal deaths in the country and 10% of maternal deaths in the states with the weakest socio-demographic indicators^a^[1]. Hospital and community-based studies suggest that complications from unsafe abortions continue to be very high, especially in the northern and central parts of the country [1-3].

Awareness of the legality of abortion remains low, leading many women to seek services outside the health system. For rural women, especially, informal and untrained providers are the only option. Within the healthcare system, several studies have found that even after four decades of the MTP Act, the majority of women and men believe that abortion is illegal in India [4-6]. All public sector health facilities are mandated to provide abortion services, but many, especially at the primary care level, are not equipped to provide safe services as recommended by the WHO and as outlined in the MTP Act. There is a shortage of trained providers in India, which is exacerbated by a policy that restricts provision of services to obstetrician-gynecologists (OB-GYNs) or specially certified general physicians. Private providers are an alternative for those who can afford them, but many private sites may be uncertified or provide poor quality of care [7,8]. Furthermore, outdated technologies such as dilatation and curettage (D&C) continue to be used by providers in both the public and private sectors [9].

Medical abortion (MA) has the potential to expand access to safe abortion services because this method is well-suited for the primary health care setting [10]. In this context, medical abortion refers to early pregnancy termination using two drugs – mifepristone and misoprostol. Mifepristone has been registered in India since 2002; at the time misoprostol was available, but its use in abortion was off-label. In 2003 the MTP Act was modified to allow OB-GYNs and other MTP-certified physicians to provide medical abortion even if their clinics were not government-certified abortion sites, followed by introduction of guidelines for medical abortion use developed by a national expert group in 2004. Finally, misoprostol was registered for use in early pregnancy termination in 2006. Although the use of medical abortion has grown in the private sector and in tertiary hospitals, the drugs are not yet routinely procured, and thus not available in many public sector facilities in India [11].

Though MA has the potential to expand access to safe services, availability of services is a necessary but not sufficient step in meeting women’s needs. It is also paramount that women become aware of services and know how to access them. Existing community intermediaries (e.g., auxiliary nurse midwives (ANMs), traditional birth attendants or *dais*, accredited social health activists (ASHAs), and *Anganwadi* workers^b^ (AWWs) [12]) could be used to bridge the knowledge gap between women and MTP services, potentially linking women to information and services by providing emotional support as women explore their options for responding to an unwanted pregnancy, providing accurate information to women about how and where to seek safe abortion services, and providing referrals to safe services. Intermediaries could also support the woman after she has an abortion and help meet her postabortion contraception needs [13-15].

Another aspect of access to safe abortion services is creating an enabling environment that will allow women to access safe services. Family members, especially husbands and mothers-in-law, serve as sources of information, gatekeepers, and sometimes proxy clients when the husband goes to the pharmacist to procure drugs instead of the woman herself. Thus, they also require intervention in order to ensure women’s access to available safe abortion services. Behavior change communication (BCC) campaigns have been used successfully in India to create an enabling environment for reproductive health issues such as family planning and HIV/AIDS [16,17].

### Intervention in Jharkhand

Ipas India is a non-governmental organization (NGO) that works in partnership with state governments to strengthen training systems, service delivery of comprehensive abortion care services, and advocacy initiatives to increase women’s access to safe abortion. Ipas has an active presence in Jharkhand, where reproductive health outcomes are poor, and availability and use of abortion services in public sector facilities is low. Jharkhand is a predominantly rural state with 76.5% of its population of 30.5 million living in villages, and over one quarter (28%) of its population is tribal. Infrastructure is not well-developed, and only 45% of villages have electricity. Contraceptive prevalence is 31.1% in Jharkhand compared to 48.5% nationally, and 60.2% of women are married before age 18 compared to 45.6% nationally [18].

Abortion services in the state are inadequate; a recent study showed poor availability and utilization of government services [8]. Women’s use of informal providers such as *dais* and ANMs, and a paucity of confidential, high-quality care has also been documented [19]. Knowledge about the legal status of abortion remains low. Less than one fourth of the 22,476 individuals polled in one rapid assessment were aware of abortion being legal under some circumstances [4]. In addition, quality of care is low. One study showed that private OB-GYNs in Jharkhand had begun to use medical abortion, but use was conservative and inaccurate information on doses and protocols was common. Though pharmacies were the most common way to obtain the drugs, only 35% of pharmacies, primarily large outlets, stocked mifepristone and misoprostol [20]. Other types of drugs, including Ayurvedic and hormonal drugs, were widely available in pharmacies and commonly sold as abortifacients [20]. Awareness of the use of mifepristone and misoprostol for medical abortion within the OB-GYN community was low, but the concept of abortion through use of traditional as well as other drugs available from pharmacists was common [20].

The present study evaluates an intervention implemented by Ipas India with the support of the government of Jharkhand. The multi-pronged intervention strategy was designed to improve availability of safe abortion services at facilities and awareness of safe abortion services, with an emphasis on MA, at the community level by providing information through community intermediaries and creating an enabling environment through a BCC campaign. The intervention, including preparation and training, was held between 2007 and 2009 and was implemented in Silli block in Ranchi district and Khunti block in Khunti district, Jharkhand. The intervention covered a total population of 161,000 in 253 villages.

The initial component of the intervention strategy was to train providers and equip facilities to ensure that safe abortion services were available when women sought services. Between May and July of 2008, 10 health providers at two public facilities (one community health center and one district hospital) and four private health facilities in the intervention blocks were trained on comprehensive abortion care (CAC), including provision of MA. These providers also received government certification as abortion providers. To ensure access to MA drugs, the mifepristone/misoprostol combination pack was provided to intervention facilities free of cost. Public sector facilities provided all CAC services free of cost, while private sector providers charged a minimal consultation fee (2-3 USD). Prior to the intervention, MA was not available in the public sector and was only available in private sector urban locations outside of and at some distance from the intervention area.

Once abortion services became locally available, a behavior change communication campaign was implemented at the end of 2008 to create an enabling environment for women to access safe abortion services. The BCC campaign was designed for a low-literacy population with limited access to mass media such as television or newspaper. Intervention activities included wall signs, posters, street drama and interpersonal communication (IPC). Wall signs and posters provided information about availability of MA at the nearest government health facility; more than 500 wall signs were painted in intervention communities. The street drama provided information on safe abortion services and introduced the concept of MA. The street drama was developed by a local NGO, and local actors did over 350 performances (at least one per village). IPC activities were conducted by trained IPC moderators who provided information on safe abortion, legal aspects and method of uterine evacuation, including MA to women in group setting through flip charts, games, and flash cards. More than 3,500 IPC sessions were conducted with around 20,000 women in the intervention communities.

In addition, community intermediaries were trained, between February and August of 2009, to provide information on safe abortion services, including MA, and referrals to safe services for women who wanted to terminate their pregnancies. A total of 709 community intermediaries (including ANMs, AWWs, ASHAs, and traditional birth attendants) from the two intervention blocks were identified through intermediary listing, and at least one intermediary from each village was trained. Because ANMs are trained health providers, they were trained separately and received additional clinical information on MA and contraception.

### Study objective

The objective of this study is to evaluate the multi-pronged intervention strategy used to increase women’s access to medical abortion in rural areas of Jharkhand, India. Specifically, this study examines the changes in knowledge and attitudes about abortion-related issues, changes in abortion care-seeking, and service utilization.

## Methods

### Study design

**Figure 1:**
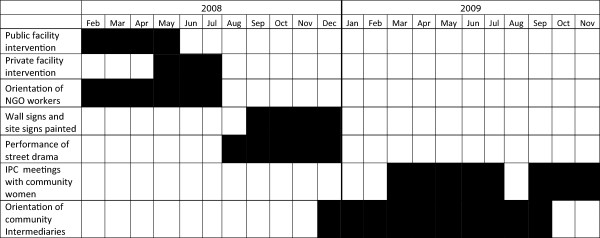
Timeline for implementation of MA Community Intervention in two selected blocks of Jharkhand.

The study uses a pre-post design with two cross-sectional household surveys of men and women, measuring knowledge, attitudes and health service utilization prior to and following the intervention. Baseline data collection occurred between December 2007 and February 2008 (before the intervention began), and endline data were collected between September and December 2010 (one year after the intervention concluded). The timing of the intervention activities is shown in Figure 1.

### Sampling and data collection

As no information was available on the primary outcome of interest (i.e. proportion of the target population aware that medical abortion is a safe option for early abortion) the minimum required sample size was estimated using the proportion of the target population with correct knowledge of legality and gestational limits of legal abortion in India. Based on data from a previous study [4], this proportion was estimated to be 1.1% of women. The minimum required sample size of 650 each for men and women was calculated based on the assumption that at the end of the two-year evaluation period, awareness would rise by at least four percentage points to 5% of women. The sample size is adequate to detect this level of change with 95% confidence and 80% power, and takes into account the design effect due to the two-stage cluster sampling approach and anticipated 2% non-response rate.

A two-stage stratified random sampling technique was used to select households for participation in the study. First, the primary sampling units (PSUs), which were individual villages, were selected through probability proportional to size (PPS) sampling. Then, households within each sample village were selected through systematic random sampling. A total of 43 villages were sampled, and in each sampled village, a structured household listing was conducted to create the sampling frame of households with eligible women and men. Sample villages larger than 500 households were segmented using the standard DHS guideline for village segmentation [18], and two segments were randomly selected.

The target population consisted of married women between the ages of 15 and 45 and married men between the ages of 18 and 49. Potential respondents were excluded from the study if they (or their spouses) had undergone sterilization three or more years prior to the survey; this group was excluded as they were unlikely to have had an induced abortion experience in the recent past. The households with eligible respondents were then selected through systematic random sampling, ensuring that both husband and wife were not included in the survey. A total of 1253 respondents, 633 women and 620 men, provided informed consent and participated in the baseline survey, and 1290 respondents, 645 women and 645 men participated in the endline survey. The non-response rate was 2% for women and 4% for men at baseline and <1% for both groups at endline. Non-response was minimized at endline by reducing the time period between the household listing and survey implementation, and up to three visits were made to households when the selected respondent was not available.

Data were collected by trained interviewers using a modular, semi-structured and pre-tested questionnaire (see Additional files 1 and 2). The questionnaire was based on one used in previous successful surveys and was pretested with abortion experts and with women from the target communities. The first module collected basic information from all respondents, including socio-demographic characteristics and reproductive histories, media exposure, health-seeking behaviors, and knowledge and attitudes about abortion. Respondents were then asked if they (or their wives) had an induced abortion during the past three years. Filter questions about missed periods, unwanted or mistimed pregnancy and any attempts made to bring on delayed periods were included in order to maximize reporting. Those who reported an induced abortion were asked further questions about the care-seeking pathway, including providers visited, methods used for induction and any complications experienced.

Health service statistics were collected monthly from each of the six health facilities in the two intervention blocks from September 2008 to May 2010.

### Data management and analysis

Questionnaires were checked for quality by field supervisors. Edited questionnaires were entered into SPSS 14.0, which was also used for data cleaning and analysis. For all outcomes of interest, proportions are reported for both men and women at baseline and endline. Chi-square tests were used to test for differences between proportions at baseline and endline, both overall and stratified by sex. Significance was evaluated at an alpha of 0.05.

Multivariate analyses were carried out through logistic regressions to understand the association between exposure to BCC activities and knowledge outcomes after adjusting for socio-demographic characteristics such as age, education, caste, religion, household structure, standard of living, and exposure to mass media. Potential confounding factors were identified through the literature, and we examined bivariate associations before inclusion in the multivariate analyses. Two multivariate models were considered for each of the three knowledge-related outcomes: the first assessed outcomes associated with exposure to any intervention, while the second assessed the outcomes associated with exposure to each intervention type. Odds ratios (OR) are reported with standard errors (SE) and associated p-values; a statistically significant OR of 3.0 would indicate that those exposed to the intervention had three times the odds of the outcome of interest as compared to people not exposed to the intervention.

## Results

### Respondent profile

Socio-demographic characteristics of study respondents were similar at baseline and endline (Table 1), with the exception of religion, caste and exposure to mass media. On average, men were aged 34, and most commonly had secondary education. Female respondents were younger (mean age = 31), and less educated. Most men and women lived in nuclear households. The majority of respondents could be classified as poor based on their ownership of household and related assets. Although the majority of respondents were Hindu, the distribution of religion differed between the two time periods; there were fewer Hindu and more Sarna respondents at endline compared to baseline (p < 0.001). Similarly, while most respondents were members of the Scheduled Tribe (ST) or Other Backward Class (OBC) castes^c^, the distribution of caste varied between the two time periods with more ST and fewer OBC at endline compared to baseline (p < 0.001). Exposure to mass media was higher at endline among men, women and overall (p < 0.001), however the increase was primarily due to radio access while television exposure remained limited (results not shown).

**Table 1:** Sociodemographic characteristics of survey respondents at baseline and endline, by sex and overall (n = 2,543)

	**Male**			**Female**			**Total**		
	**Baseline**	**Endline**		**Baseline**	**Endline**		**Baseline**	**Endline**	
	**(n = 620)**	**(n = 645)**		**(n = 633)**	**(n = 645)**		**(n = 1,253)**	**(n = 1,290)**	
	**%**	**%**	**p-value**	**%**	**%**	**p-value**	**%**	**%**	**p-value**
**Current age**									
15-19 years	0.2	0.00	0.307	6.8	8.7	0.206	3.5	4.3	0.282
20-29 years	28.1	27.9	0.950	52.3	52.4	0.967	40.3	40.2	0.939
30-39 years	44.2	45.7	0.581	34.6	33.3	0.633	39.3	39.5	0.922
40 and above	27.6	26.4	0.623	6.3	5.6	0.577	16.8	16.0	0.553
**Average age (years)**	34.3	33.9	0.341	28.0	28.1	0.766	31.1	31.0	0.739
**Education**									
Up to primary	34.8	36.7	0.480	57.2	55.7	0.581	46.1	46.2	0.970
Secondary	51.1	50.5	0.834	39.7	37.7	0.467	45.3	44.1	0.535
High school & above	14.0	12.7	0.490	3.2	6.7	0.004	8.5	9.7	0.313
**Average schooling (years)**	8.6	8.2	0.076	7.5	7.6	0.662	8.1	8.0	0.534
**Religion**									
Hindu	77.7	59.8	<0.001	76.5	60.2	<0.001	77.1	60.0	<0.001
Muslim	1.9	3.9	0.041	2.1	3.4	0.137	2.0	3.6	0.012
Christian	5.5	4.7	0.499	5.4	4.7	0.555	5.4	4.7	0.371
Sarna	14.8	31.6	<0.001	16.1	31.8	<0.001	15.5	31.7	<0.001
**Caste**									
Scheduled caste (SC)	9.4	6.1	0.027	5.8	8.5	0.064	7.6	7.3	0.776
Scheduled tribe (ST)	40.3	55.6	<0.001	33.5	50.7	<0.001	36.9	53.1	<0.001
Other Backward class (OBC)	43.7	37.3	0.018	55.3	38.4	<0.001	49.6	37.9	<0.001
General	6.6	1.1	<0.001	5.4	2.3	0.005	6.0	1.7	<0.001
**Family Type**									
Nuclear	64.0	57.1	0.011	52.9	51.2	0.529	58.4	54.1	0.028
Joint/extended	36.0	42.9	0.011	47.1	48.8	0.529	41.6	45.9	0.010
Exposure to mass media	65.5	72.1	0.011	50.1	83.7	<0.001	57.7	77.9	<0.001

### Abortion knowledge

At both baseline and endline, respondents were asked a series of questions related to their knowledge of the legality and indications for abortion. Awareness that abortion is legal was higher at endline, 57.6% at endline compared to 19.7% at baseline (p < 0.001) overall, with similar outcomes for men and women (data not shown). Participants were asked which indications they believed to be legal for abortion in India. Knowledge of the correct indications and gestational limits of legal abortion was higher at endline (44.3%) as compared to baseline (21.4%, p < 0.001) for all conditions, and for both men and women (Table 2).

**Table 2:** Knowledge of legal indications of abortion at baseline and endline, by sex and overall (n = 2,543)

	**Male**			**Female**			**Total**		
	**Baseline**	**Endline**		**Baseline**	**Endline**		**Baseline**	**Endline**	
	**(n = 620)**	**(n = 645)**		**(n = 633)**	**(n = 645)**		**(n = 1,253)**	**(n = 1,290)**	
	**%**	**%**	**p-value**	**%**	**%**	**p-value**	**%**	**%**	**p-value**
**Legal indications**									
Pregnancy due to contraceptive failure	13.5	36.1	<0.001	13.9	42.6	<0.001	13.7	39.4	<0.001
Rape	14.4	59.5	<0.001	12.8	59.7	<0.001	13.6	59.6	<0.001
Women's health in danger	13.9	36.3	<0.001	17.5	53.2	<0.001	15.7	44.7	<0.001
Serious fetal deformity	13.5	30.4	<0.001	15.2	44.0	<0.001	14.4	37.2	<0.001
**Gestational limit**									
Legal for pregnancy only up to 20 weeks^1^	8.1	0.5	<0.001	4.1	0.0	<0.001	6.1	0.2	<0.001
**Correct knowledge of the law**^**2**^	**18.9**	**30.7**	**<0.001**	**23.9**	**57.8**	**<0.001**	**21.4**	**44.3**	**<0.001**

^1^Pregnancy beyond 20 weeks is not legal under the MTP Act.

^2^This includes both legal indications and the gestational limit. Under the 1971 Medical Termination of Pregnancy (MTP) Act, abortion is legal in India for a wide range of indications: to save the woman’s life and in cases of grave threats to the woman’s physical or mental health, as well as in cases of rape, fetal abnormality or failure of contraception for married women.

Table 3 shows changes in participants’ awareness of abortion sources. Intervention sites refer to those facilities specifically included in this intervention project and approved by the government to provide abortion services, including medical abortion. Non-intervention sites refer to those facilities where services were not available at the time of the project, although the public non-intervention sites were legally allowed to do so. At baseline, participants’ awareness of public sector intervention sites for abortion services was already high, but was still significantly higher after the intervention. At endline, over 90% of both men and women recognized that abortion was available at public intervention sites. Men reported a significant drop in their belief that public non-intervention sites provide abortion (from 22.4% at baseline to 5.9% at endline; p < 0.001), although the same was not observed for women. Awareness of private intervention abortion sites also increased significantly from the baseline for both groups. Interestingly, there was an even larger increase in belief that private non-intervention sites provide abortion (although they did not offer these services at the time of the project). Both male and female participants at endline had significantly lower rates of selecting chemist shops or pharmacies as sources of abortion care compared to baseline. Furthermore, by endline very few women (1.6%) or men (5%) reported having "no idea" where to obtain an abortion, a significant drop from baseline reporting.

**Table 3:** Awareness of abortion availability and methods at baseline and endline, by sex and overall (n = 2,543)

	**Male**			**Female**			**Total**		
	**Baseline**	**Endline**		**Baseline**	**Endline**		**Baseline**	**Endline**	
	**(n = 620)**	**(n = 645)**		**(n = 633)**	**(n = 645)**		**(n = 1,253)**	**(n = 1,290)**	
	**%**	**%**	**p-value**	**%**	**%**	**p-value**	**%**	**%**	**p-value**
**Abortion service sites**^**1**^									
Public intervention site^2^	81.3	90.7	<0.001	78.4	90.7	<0.001	79.8	90.7	<0.001
Public non-intervention site^2^	22.4	5.9	<0.001	19.6	18.4	0.603	21.0	12.2	<0.001
Private intervention site^2^	0.0	16.4	<0.001	0.0	12.6	<0.001	0.0	14.5	<0.001
Private non-intervention site	4.8	33.3	<0.001	3.9	43.3	<0.001	4.4	38.3	<0.001
Chemist shop/pharmacy	21.3	7.6	<0.001	24.3	2.9	<0.001	22.8	5.3	<0.001
No idea/do not know	12.9	4.7	<0.001	15.0	1.6	<0.001	14.0	3.1	<0.001
**Abortion providers**^**1,3**^									
Any health worker^4^	22.0	0.3	<0.001	18.2	1.8	<0.001	20.1	1.0	<0.001
Any qualified doctor^5^	33.1	26.5	0.685	26.8	31.0	0.116	29.9	28.7	0.507
Trained & certified doctor^6^	30.7	71.3	<0.001	37.4	59.7	<0.001	34.1	55.7	<0.001
No idea/do not know	14.3	1.9	<0.001	17.5	7.5	<0.001	15.9	4.6	<0.001
**Abortion methods**^**1,7**^									
MA	15.8	86.8	<0.001	13.0	72.4	<0.001	14.4	79.6	<0.001
MVA	5.5	32.7	<0.001	5.7	28.5	<0.001	5.6	30.6	<0.001
D&C	4.7	18.0	<0.001	3.6	30.5	<0.001	4.2	24.3	<0.001

^1^Respondents were able to select all that apply; the total for each category will not sum to 100%.

^2^Correct responses according to the India MTP Act.

^3^Percentage computed among respondents reporting awareness of abortion services.

^4^Includes Nurse, ANM, RMP, etc.

^5^Any medical doctor

^6^Trained and certified medical doctors. Note that the BCC campaign specifically promoted the idea that only CAC trained medical doctors can provide abortion services.

^7^Percentage computed among those who know of any abortion methods.

Respondents’ knowledge that trained and certified providers were the most suitable choice for a legal abortion was significantly higher after the intervention (Table 3). Both men and women showed improvement on this indicator (increased from 34.1% to 55.7% after intervention, p < 0.001), as well as a concomitant reduction in the perception that any health worker can provide abortion (20.1% vs 1.0%, p < 0.001). In addition, the percentage of respondents who had "no idea" about appropriate abortion sites or providers was significantly lower at endline than at baseline for both men and women. Knowledge of MA as a method of abortion increased from 14.4% at baseline to 79.6% at endline (p < 0.001), this pattern was true for both men and women. Knowledge of D&C also increased significantly from baseline to endline (p < 0.001). Although not asked at baseline, knowledge of the legal gestational limit for MA (7 weeks at the time of the project) was approximately 66.4% at endline, with no difference between men and women.

### Abortion experience

**Figure 2:**
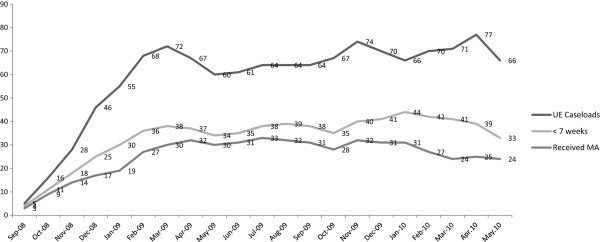
MTP Service Statistics in Intervention Facilities – overall, <7 weeks gestation, and MA; September 2008 – May 2010.

At baseline, only one intervention site was routinely providing legal abortion services using MVA, by endline all six intervention sites were routinely providing abortion services using MVA and MA. Figure 2 shows a dramatic rise in the uterine evacuation caseload in the months following the onset of the intervention. Furthermore, over half of the women presented for abortion by 7 weeks gestation, and a large proportion of their abortions were completed using MA.

Reports of induced abortion (Table 4) were significantly higher at endline than at baseline (11.3% at endline compared to 4.2%; p < 0.001). Both men and women report that men are the primary abortion decision-makers. Use of intervention sites for termination of pregnancies was higher after the intervention, as reported by men and women, increasing among all respondents from 28.3% to 66.6% at endline (p < 0.001), however use of informal providers did not decrease as a result of the intervention. Correspondingly, reported self-induced abortions were lower after the intervention (20.8% at baseline vs 2.7% at endline, p < 0.001). Abortion method also changed as a result of the intervention with use of medical abortion (16.7% vs. 47.9%, p < 0.001) and MVA (19.0% vs. 48.6%, p0.001) significantly higher following the intervention, while use of D&C decreased after the intervention (47.6% vs. 2.1%, <0.001). There was a non-statistically significant decline in self-reported postabortion complications (37.5% to 19.0%, p = 0.059). Women’s mean reported cost of abortion decreased following the intervention from 700 INR to 615 INR for women presenting for induced abortion. Women reported no change in the uptake of postabortion contraception after the intervention.

**Table 4:** Induced abortion incidence and decision-making in last three years at baseline and endline, by sex and overall (n = 2,543)

	**Male**			**Female**			**Total**		
	**Baseline**	**Endline**		**Baseline**	**Endline**		**Baseline**	**Endline**	
	**(n = 620)**	**(n = 645)**		**(n = 633)**	**(n = 645)**		**(n = 1,253)**	**(n = 1,290)**	
	**%**	**%**	**p-value**	**%**	**%**	**p-value**	**%**	**%**	**p-value**
**Reported induced abortion in last 3 years**									
n	24	59		29	87		53	126	
%	3.9	9.1	<0.001	4.6	13.5	<0.001	4.2	11.3	<0.001
**Primary decision maker**^**1**^									
Wife	12.5	30.5	0.087	55.2	42.5	0.236	35.8	37.7	0.813
Husband	83.3	69.5	0.195	37.9	55.2	0.108	58.5	61.0	0.753
Mother in law			---	3.4	1.1	0.410	1.9	0.7	0.452
Other relatives	4.2		---	3.4	1.1	0.410	3.8	0.7	0.114
**Provider who performed the termination**^**1**^									
Doctor at intervention site	29.2	79.7	<0.001	27.6	57.5	0.005	28.3	66.4	<0.001
Doctor at private non-intervention site	41.7	6.8	<0.001	31.0	14.9	0.055	35.8	11.6	<0.001
Health worker^2^	4.2	3.4	0.874	6.9	8.0	0.841	5.7	6.2	0.894
Informal providers^3^	12.5	8.6	0.587	17.2	17.2	1.000	15.1	13.7	0.802
Self-induced^4^	12.5	1.7	0.038	27.6	3.4	<0.001	20.8	2.7	<0.001
**Abortion method**^**5**^									
Medical (MA)	22.2	51.7	0.028	12.5	45.2	0.004	16.7	47.9	<0.001
MVA^6^	16.7	43.7	0.042	20.8	52.4	0.006	19.0	48.6	0.001
D&C^7^	55.6	3.4	<0.001	41.7	1.2	<0.001	47.6	2.1	<0.001
Other - traditional method	0.0	1.7	0.575	25.0	2.4	<0.001	14.3	2.1	0.001
Can’t say	5.6						2.4		
**Received post abortion contraception**^**2**^				66.7	67.9	0.912			
**Post abortion complication**^**2**^				37.5	19.0	0.059			

^1^Percentage computed among the n = 146 who reported induced abortion in last 3 years. The sample sizes are as follows: male baseline = 24; male endline = 59; female baseline = 29; female endline = 87; total baseline = 53; total endline = 146.

^2^Health worker includes nurses, ANMs, AWWs, and ASHAs.

^3^Informal providers include those not approved by the government to offer abortion services, such as chemist shops, rural medical practitioners, and traditional healers.

^4^Self-induction is defined as women who tried home remedies, e.g., herbal concoctions, external massage, inserting objects into the vagina, and unknown tablets.

^4^Percentage computed among the n = 142 who reported induced abortion by provider rather than self-induced. The sample sizes are as follows: male baseline = 18; male endline = 58; female baseline = 24; female endline = 84; total baseline = 42; total endline = 142.

^6^MVA is defined as surgical methods done without general anesthesia, as described by women.

^7^D&C is defined as surgical methods done with general anesthesia, as described by women.

### Exposure to campaign/sources of abortion knowledge

**Figure 3:**
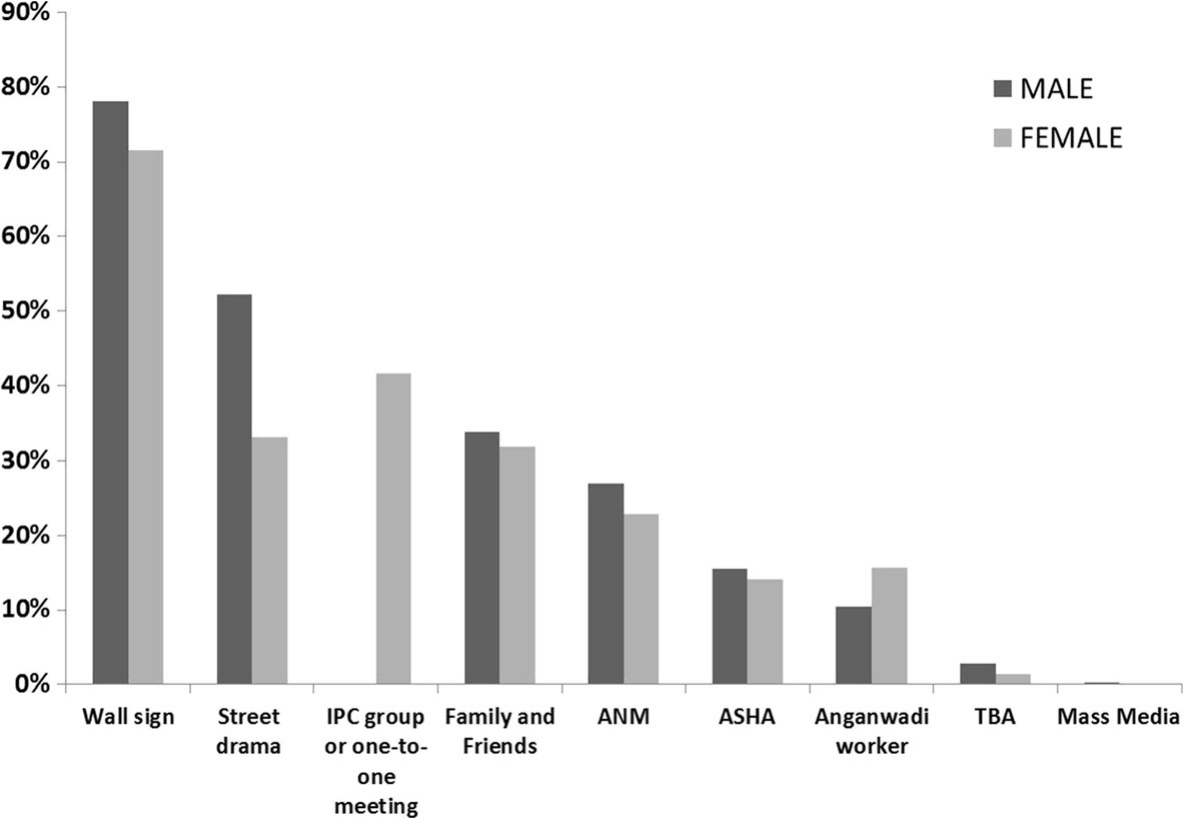
Source of safe abortion messages at endline, by sex (n = 1,290).

After the intervention, 85% of respondents reported being exposed to safe abortion messaging. As shown in Figure 3, women’s primary sources of information on safe abortion were wall signs (71.6%), IPC group or one-to-one meetings (41.6%) and community intermediaries (40.9%). Men reported receiving safe abortion messages most often from wall signs (78.0%), street dramas (52.2%) and community intermediaries (38.4%). Family and friends were also an important source of information for both men and women.

Table 5 describes the effects of different intervention components on increasing women’s awareness of abortion issues, after adjusting for socio-demographic characteristics. The three models examining overall exposure to any intervention suggest there are positive associations between any BCC intervention exposure and each knowledge outcome. In particular, respondents who were exposed to any intervention activity have nearly 11 times the odds of knowing that abortion is legal compared to those who were not exposed (p < 0.001), nearly 19 times the odds of knowing the correct gestational age limit for MA compared to those who were not exposed (p < 0.001), and over three times the odds of knowing at least one correct location for CAC services compared to those who were not exposed (p < 0.001). When considering various intervention types, the pattern differs for each knowledge outcome. In particular, even after adjusting for socio-demographic characteristics, exposure to IPC, wall signs, street dramas and community intermediaries were all positively associated with knowing that abortion is legal at endline; exposure to IPC, wall signs, and street dramas were positively associated with knowing the correct gestational limit for MA. However, only the interpersonal communication intervention type was associated with knowing a correct source of CAC services. Respondents who were exposed to the IPC intervention had nearly 5 times the odds of knowing a correct source of CAC services compared to those who were not exposed, after adjusting for socio-demographic characteristics.

**Table 5:** **Multivariate analyses of the BCC intervention on three abortion knowledge outcomes, (n=1,290)**^**1**^

**Exposed to**	**Model 1**			**Model 2**			**Model 3**		
	**Know abortion is legal**			**Correct knowledge on legal gestation of MA**			**Know at least one correct source of CAC services**		
	**Odds ratio**^**2**^	**SE**	**p-value**	**Odds ratio**^**2**^	**SE**	**p-value**	**Odds ratio**^**2**^	**SE**	**p-value**
**Model of any intervention**									
Any intervention	10.8	0.31	<0.001	18.8	0.53	<0.001	3.3	0.37	0.001
**Model of intervention type**									
Interpersonal communication (IPC)	3.2	0.25	<0.001	2.7	0.21	<0.001	4.8	0.67	0.019
Wall sign	4.8	0.30	<0.001	2.7	0.31	0.002	0.9	0.56	0.931
Street drama	3.0	0.26	<0.001	2.7	0.21	<0.001	0.6	0.53	0.288
Community intermediary	2.8	0.25	<0.001	1.2	0.21	0.388	1.4	0.48	0.466

^1^This table presents six adjusted models: two for each knowledge outcome: (1) considering the effect of exposure to any intervention after adjusting for control variables and (2) considering the effect of exposure to each intervention type, after adjusting for control variables.

^2^Controlling for age, education, caste, religion, family type, standard of living, and exposure to mass media.

## Discussion

This study suggests that the multi-pronged intervention of facility/provider readiness, deployment of community intermediaries and a coordinated BCC campaign on abortion was successful in improving use of and knowledge about safe MA services in Jharkhand. Abortion caseload at intervention facilities and, more specifically, both eligibility for (presenting at <7 weeks gestation) and the proportion of women using MA, increased over time. It is important to note that the increase in reported abortion does not reflect an actual increase in abortion incidence, rather it reflects a shift from less safe methods to safe, legal abortion services and potentially a decrease in abortion-related stigma. Even after controlling for sociodemographic factors, community members were more likely to know 1) abortion is legal in India; 2) the legal gestational limits for MA; and 3) at least one safe, legal source for abortion services. Based on our review of the literature, this intervention is the first documented behavior change communication intervention on abortion in Jharkhand, and likely in India as a whole, although Ipas conducted subsequent activities in Maharashtra, Bihar and Jharkhand (November 2008) and Madhya Pradesh (2013) and a youth-focused intervention in Jharkhand (2012-2013).

Despite rigorous use of random sampling procedures, caste and religion varied between the baseline and endline samples. The intervention area is dominated by the scheduled caste and scheduled tribe populations. In addition, the Sarna religion is specific to the tribal population of Jharkhand [21]; thus, having a larger sample of scheduled tribe population at endline explains the correlated increase in respondents who identify as tribal. While the deviation in tribal populations between baseline and endline may be due random variation because the overall tribal population is relatively static in Jharkhand, it is possible that the measured differences reflect more seasonal migration of the tribal population at baseline [22]. Nonetheless, inclusion of more tribal population at endline is likely to have resulted in an underestimate of intervention impact because the non-tribal populations had better socioeconomic status which was positively associated with outcomes.

Study findings suggest good penetration of the BCC messaging overall, as the majority of respondents report exposure to safe abortion messages. Furthermore, multivariate analyses demonstrate that the combined strategies were successful in increasing women’s knowledge of legal abortion, gestation for medical abortion and knowledge of proper sources of CAC services. Wall signs, street dramas and friends/families were identified sources of information for all participants and were associated with the simpler messages, while the more complicated messages (knowing at least one correct source for CAC services) was more effectively communicated through IPC than other BCC methods. It is possible that the overall strategy could benefit from simplifying messages or relying more heavily on IPC. Subsequent abortion-focused BCC campaigns in India resulted in similar findings, suggesting that such campaigns can be effective in increasing awareness and utilization of safe abortion services [23-25].

In addition to increased knowledge about availability of abortion services at public and private sector intervention sites observed among men and women, men also reported a significant drop in their belief that public non-intervention sites provide abortion; although it is true that the public non-intervention sites were not providing abortion services at the time, this perception could be problematic in the long run public non-intervention sites *should be* providing safe abortion services. Future work should emphasize ensuring availability of abortion services at all legally approved sites. Another potential concern is with the reported increase in knowledge of D&C as a method of abortion from 4.2% at baseline to 24.3% at endline (p < 0.001). It is possible that this might result in more women seeking D&C in lieu of the WHO-preferred first trimester abortion methods of MVA and MA.

Service statistics and household interviews evinced a significant increase in MA use over the intervention period. Accordingly, increased access to abortion services through the formal health system and certified providers was reported at endline as compared to baseline. Over time there was a significant improvement in abortion technology, with increased use of both WHO approved methods – MA and MVA – and a corresponding decrease in D&C and traditional methods. In addition, there was a positive shift in seeking abortion services earlier in gestation, many by the 7-week legal limit at that time. Although we cannot distinguish between the effects of the facility-level intervention from the effects of the BCC campaign, it is likely that the multi-pronged nature of the intervention led to changes in both abortion service use and community-level knowledge about abortion during a relatively short time frame.

Before the intervention abortion service availability in Jharkhand was limited, in part due to a lack of MA drugs. In response, MA drugs were provided to both public and private intervention sites as part of the intervention package. Public sector facilities provided MA and MVA abortion services free of cost, while private sector providers charged a minimal consultation fee (2-3 USD). Cost of abortion services decreased between baseline and endline which likely reflects women accessing care earlier in gestation as well as the effect of subsidized MA drugs. Even after the intervention, MA and MVA services in India’s public health sector continue to be free to women, although private sector providers have more leeway in charges. If increased cost of MA in the private sector limits women’s access to abortion beyond shifting them to public facilities, the impact of our intervention may be diminished and affect the feasibility of replicating the intervention in other settings. Note that in the private sector MA continues to be a less expensive than surgical methods of abortion.

No significant change was reported in provision of postabortion contraception over the course of the intervention; however, both baseline and endline rates are high (approximately 67%) when compared to the regional contraceptive prevalence rate for modern methods, 25.2% [17]. Increased use of MA may itself account for the observed lack of improvement in postabortion contraceptive uptake; although the standard CAC guideline suggests that providers offer a contraceptive method after medical abortion during a return visit two weeks after the initial procedure, many women who have had a successful MA do not choose to return for follow-up [26].

The findings of this study should be viewed in light of the limitations; as with any pre/post evaluation design this study is unable to account for historical or maturation changes that may have affected the outcomes. The BCC intervention was focused on married men and women, omitting the perspective of unmarried women with unwanted pregnancy. In addition, exposure information was limited to self-report. Very small numbers of women reported any experience with abortion, which limits the generalizability of their data. Moreover, there is an overlap in the recall period of 6-8 months, which suggests that abortion was underreported at baseline and that higher reporting at endline could be associated with normalization of abortion (decrease in stigma) and improved knowledge on legality of abortion. Finally, we do not know how sustainable the changes in knowledge might be, nor the impact of the information itself changing over time (e.g., the gestational limits for MA have increased from 49 to 63 days).

## Conclusions

Despite these limitations, this evaluation suggests a positive impact of the project - the first community intervention on safe abortion services in Jharkhand where no other organizations were working in the area of abortion at the time. This intervention increased knowledge about abortion and improved access to safe abortion care. The findings from this evaluation have led to implementation of similar interventions in other districts of Jharkhand and other states of India.

## Endnotes

^a^Also called the Empowered Action Group (EAG) states under the National Rural Health Mission. EAG states include Bihar, Chhattisgarh, Jharkhand, Madhya Pradesh, Odisha, Rajasthan, Uttar Pradesh and Uttarakhand.

^b^*Anganwadi* workers (AWWs) are health workers selected by their home communities to receive 4 months training in reproductive health (including antenatal care and contraception), nutrition and child immunizations. The AWWs each serve a population of approximately 1000 people through an *Anganwadi*, which means "courtyard shelter" in Hindi.

^c^Under Article 340-342 of the Indian Constitution, the Government of India (GoI) classifies some of its citizens based on their social and economic conditions. Scheduled Caste, Schedule Tribe and Other Backward Class are the three broad designations for historically disadvantaged caste groups. Scheduled Caste and Scheduled Tribe groups are the most socially and economically disadvantaged groups in India, and the Scheduled Tribes receive the most government support, as they have been farthest removed from mainstream Indian society. The term ‘backward class’ is a collective term used by the government of India for castes which are economically and socially disadvantaged.

## Abbreviations

ANM: Auxiliary nurse midwives; ASHA: Accredited social health activists; AWW: *Anganwadi* workers; BCC: Behavior change communication; CAC: Comprehensive abortion care; D&C: Dilatation & curettage; GoI: Government of India; INR: Indian Rupee; IPC: Interpersonal communication; MA: Medical abortion; MTP: Medical Termination of Pregnancy; MVA: Manual Vacuum Aspiration; NGO: Non-governmental organization; OBC: Other Backward Class; OB-GYNs: Obstetrician-gynecologists; OR: Odds ratio; SE: Standard error; ST: Scheduled tribe; USD: United States Dollar.

## Competing interests

The authors declare that they have no competing interests.

## Authors’ contributions

SKB, TB, BG, and SB contributed to the design and implementation of the intervention. SKB, JW and KA participated in the design of the evaluation, data management and analysis. KA led the write-up; all authors contributed to review and finalization of the manuscript. BG was employed at Ipas at the time of the study. All authors read and approved the final manuscript.

## Pre-publication history

The pre-publication history for this paper can be accessed here:

http://www.biomedcentral.com/1472-6963/14/227/prepub

## Supplementary Material

Additional file 1 **Abortion related knowledge and care seeking behavior and practice in Jharkhand: A KABP follow-up study among women and men of reproductive age.** Men’s questionnaire. Click here for file

Additional file 2 **Abortion related knowledge and care seeking behavior and practice in Jharkhand: A KABP follow-up study among women and men of reproductive age.** Women’s questionnaire. Click here for file
