# Supplementary material for: Evaluation of a multi-pronged intervention to improve access to safe abortion care in two districts in Jharkhand
Source: BMC Health Serv Res. 2014 May 21;14:227. doi: 10.1186/1472-6963-14-227 (PMC4035795; doi:10.1186/1472-6963-14-227)
Supplement: Additional file 1 — Abortion related knowledge and care seeking behavior and practice in Jharkhand: A KABP follow-up study among women and men of reproductive age. Men’s questionnaire. [file 1472-6963-14-227-S1.pdf]

CMS social

HOUSEHOLD LEVEL SURVEY

Abortion related knowledge and care seeking behavior and practice in Jharkhand: A KABP follow-up study among women and men of reproductive age

MEN'S QUESTIONNAIRE

PSU IDENTIFICATION	CODES
1. District: ______________________	☐
2. Block : ______________________	☐☐☐
3. Panchayat: ______________________	☐☐☐
4 Village: ______________________	☐☐☐
**CLIENT IDENTIFICATION:**	
5. Respondent's ID No (Record from listing sheet): ______________________	☐☐☐
6. Address (with landmark): ______________________	
**INTERVIEW STATUS:**	
7. Date of interview<br>**Date** **Month** **Year**<br>☐☐ ☐☐ ☐☐☐☐	
8. Interview Status: ______________________<br>1 Completed<br>2 Partly Completed<br>3 Refused by respondent<br>4 Refused by wife/ attendants<br>5 Not at Home<br>6 Other (specify)______________________	☐

Name & Code	**Investigator**	**Monitored by**	**Edited by**
	____________<br>____________<br>☐☐	____________<br>____________<br>☐☐	____________<br>____________<br>☐☐

CMS social

INTRODUCTION AND CONSENT

नमस्ते मेरा नाम ——————————————— है। मैं सेन्टर फार मीडिया स्टडीज की तरफ से हूँ। जो कि एक गैर सरकारी शोध संस्था है।

मेरे साथ बातचीत तथा समय देने का आपका घन्यवाद। यह सर्वे सेन्टर फार मीडिया स्टडीज और आइपास, के द्वारा करवाया जा रहा है। आइपास दिल्ली में स्थित अन्तर्राष्ट्रीय संस्था है जो महिलाओं के प्रजनन स्वास्थ्य और अधिकारों खासकर सुरक्षित गर्भपात की सेवाओं को प्राप्त करने में उनकी मदद करती है। इस अध्ययन के द्वारा हम महिलाओं के स्वास्थ्य, खासकर गर्भ के बारे में और महिलाएँ गर्भ को रोकने के लिए क्या कर सकती है और ऐसी परिस्थिति में जब वह गर्भ ना चाहते हुए भी गर्भ धारण कर ले के बारे में भी जानना चाहते है। और स्वास्थ्य सेवाऐं जो इस तरह की परिस्थितियों में महिलाओं के लिए उपलब्ध है। यह अध्ययन झारखण्ड के दो जिले में हो रहा है और हम आप जैसे पुरूषो से इन मुद्दों के बारे में बात कर रहे है। यह जानकारी महिलाओं की इस तरह की जरुरतों के लिए कार्यक्रम को बेहतर बनाने और जवाबों को समझने में हमारी मदद करेगी।

इस अध्ययन मे भाग लेने के लिए मै आपकी अनुमति लेना चाहूँगा। खासतौर से मै आप व आपकी पत्नी के गर्भ से सम्बधित उनके अनुभव के बारे में आपसे प्रश्न पूछना चाहूँगा। इस सर्वे में भाग लेना स्वैच्छिक है। यदि आप सर्वे में भाग नही लेना चाहते है या साक्षात्कार के दौरान किसी भी समय साक्षात्कार बन्द कर देते है, तो किसी भी क्लीनिक व अन्य जगहों से आपको मिलने वाली सेवाओं पर वर्तमान या भविष्य में कोई असर नही पड़ेगा।

हम आपको भरोसा दिलाते है कि जो भी जानकारी आप हमें देंगें उन्हे पूर्ण रूप से गुप्त रखा जायेगा और इस जानकारी को सिर्फ महिलाओं की जरूरतो को समझने और उनसे सम्बन्धित कार्यक्रमों के विकास के लिए इस्तेमाल किया जायेगा। जब इस अध्ययन के नतीजे प्रकाशित होंगे, आपकी गोपनियता को पूर्ण रूप से सुरक्षित रखा जायेगा। हमे उम्मीद है कि आप हमारे प्रश्नो का जबाव देने के लिए तैयार होंगी, आपकी राय और अनुभव हमारे लिए महत्वपूर्ण और लाभदायक होंगे।

यदि आप साक्षात्कार के लिए तैयार हैं, तो हम ऐसी जगह बातचीत करेंगे जहां कोई हमारी बातचीत ना सुन सके, इस बातचीत मे लगभग आधा घंटे का समय लगेगा।

यदि आपका इस अध्ययन के बारे में कोई प्रश्न है तो श्री देवाशिश सिन्हा - फोन न0 09334196224, से सम्पर्क कर सकते है।

उत्तरदाता का हस्ताक्षर ....दिनांक ....

मैने इसकी पुष्टि कर ली है कि उत्तरदाता साक्षात्कार के लिए सहमत/असहमत है। (जो लागू नही उसे काट दें)

साक्षात्कारकर्त्ता के हस्ताक्षर...दिनांक ....

उत्तरदाता साक्षात्कार के लिए सहमत ....1 ↓ उत्तरदाता साक्षात्कार के लिए असहमत........ 2 समाप्त →

क्या मै अब साक्षात्कार शुरू कर सकता हूँ?

CMS social

Thank you for agreeing to take part in this study. I want to start by asking you a few general questions about you and your household. **इस सर्वे में भाग लेने हेतू सहमति के लिए धन्यवाद। अब मैं आपके एवं आपके परिवार संबंधित कुछ सामान्य प्रश्न पूछना चाहूँगा।**

SECTION 1: RESPONDENT'S BACKGROUND & HOUSEHOLD INFORMATION

Q.No	Questions and filters	Coding Categories	Skip to
100	How old were you at your last birthday?<br>आपने अपने आखिरी जन्मदिन पर कितने वर्ष पूरे किए?<br>[COLLECT AGE IN COMPLETED YEARS]<br>आयु पूर्ण वर्षों में लिखें,	Age in completed years<br>आयु पूर्ण वर्षों में ☐☐	
101	Have you ever attended school?<br>**क्या आप कभी स्कूल गऐ हैं?**<br>If Yes, what is the highest level of schooling you have successfully completed?<br>यदि हाँ, तो आपने सबसे ऊँची कौन–सी कक्षा सफलतापूर्वक पूरी करी है?	Completed years of schooling<br>स्कूल में शिक्षा पूर्ण वर्षो में लिखें ☐☐<br>Never attended school……………..………95<br>स्कूल कभी नहीं गए	
**CHECK BOX NO. 1**	**IF Q101= LESS THAN 05 or 95 ⟶ Q102**	**IF Q101= 05 OR MORE⟶ Q103**	
102	Can you read and write?<br>क्या आप पढ़ लिख सकते है?	Read only/सिर्फ पढ़ना……………………..…..1<br>Write only/सिर्फ लिखना…………………...…..2<br>Read & Write both/पढ़ना और लिखना दोनो…..3<br>Can't read and write/पढ़ना और लिखना नहीं आता……………………………………..…...4	
103	What is your current marital status?<br>Are you……<br>आपकी अभी वैवाहिक स्थिति क्या है?<br>क्या आप.......	Currently Married/वर्तमान में शादी–शुदा………1<br>Separated/ Divorced/छोड़ दिया/तलाकशुदा......2<br>Widower/विधुर……………..……………..…....3<br>Never Married/शादी नहीं हुई…………….….4	 <br>►**End**<br>►**End**<br>►**End**
104	How old were you at the time of your marriage?<br>आपकी शादी के समय आपकी आयु क्या थी?	Age in completed years<br>आयु पूर्ण वर्षो में लिखे ☐☐	
105	How old were you when you started staying with your wife? जब आप अपनी पत्नी के साथ रहने लगे उस समय आपकी क्या आयु थी?	Age in completed years<br>आयु पूर्ण वर्षो में लिखे ☐☐	
106	What is the religion you follow?<br>आप किस घर्म के है?	Hindu/हिन्दु……………........……….……......1<br>Muslim/मुस्लिम……………....……….………..2<br>Christian/ईसाई……………..………….……......3<br>Sarna/सरना…...……………………...…......4<br>Others (specify)__________________________5<br>अन्य (स्पष्ट करें)	
107	Do you belong to scheduled caste, scheduled tribe or other backward class or general caste?<br>क्या आप अनुसूचित जाति या अनुसूचित जन–जाति या अन्य पिछड़े वर्ग या सामान्य वर्ग से हैं?<br>**INS: If respondent doesn't know specify the caste**<br>**निर्देशः यदि उत्तरदाता अपनी ग्रुप नहीं बता सकता है, तो जाति का नाम लिखें**	SC/एस सी…………………………………......1<br>ST/एस टी……………………...………...2<br>OBC/ओबीसी…....………..………………....3<br>General/सामान्य……....……………………..4<br>Caste Name_________________________<br>जाति का नाम	

CMS social

No.	Question	Response / Code	Skip
108	Now I would like to know about your family composition that is whether you are living in a nuclear, or extended or joint family.<br>अब मे आपके परिवार के संबध में जानना चाहूँगा कि आपका एकल परिवार है या संयुक्त<br><br>Please tell me the names of all household members one by one and their relationship to the head of the household. ***कृपया आप परिवार के सारे सदस्यों का एक–एक कर नाम बताए तथा घर के मुखिया से उनके संबध भी बताऐं।***	Nuclear Family/एकल परिवार .... 1<br>Extended Family/विस्तारित परिवार .... 2<br>Joint Family/संयुक्त परिवार .... 3<br><br>**INS:** NOTE DOWN THE NAMES AND RELATIONS IN YOUR NOTE BOOK AND CIRCLE THE APPROPRIATE CODE. SEE THE FAMILY MATRIX BELOW<br>**निर्देश:** नाम और उनका संबंध अपनी पुस्तिका में लिखें एंव उचित कोड को चिन्हित करें। नीचे दिया गया परिवार मेट्रीक्स देखें	

**Type of Family**<br>परिवार के प्रकार	**Wife**<br>पत्नी	**Children (Unmarried)**<br>बच्चे (विवाहित नही है)	**Father-In-law/ Mother-in-law/ Father/Mother**<br>ससुर/सास /माता–पिता	**Brother/Sister [Unmarried]**<br>भाई/बहन (विवाहित नही)	**Brother/Sister's family and/or their children**<br>भाई/बहन परिवार और उनके बच्चे
**Nuclear**					
**Extended**					
**Joint Family**					
**Joint Family**					

No.	Question	Response / Code	Skip
109	Have you done any work in the last 3 months for which you have been paid in cash, or kind or both?<br>क्या आपने पिछले तीन माह में कोई काम किया जिसके लिये आपको नकद या वस्तु या दोनो प्राप्त हुए हो?	Yes, only cash/हाँ,सिर्फ नकद .... 1<br>Yes, only kind/हाँ,सिर्फ वस्तु .... 2<br>Yes, cash & kind both/हाँ,सिर्फ नकद एवं वस्तु .... 3<br>No, did not work/नहीं, काम नही करते 4	4 → 111
110	What kind of such work have you mainly done in the last 3 month?<br>आपने पिछले तीन माह में मुख्य रूप से किस प्रकार का काम किया?	Cultivation/Family farm/खेती/परिवारिक खेती .... 1<br>Agricultural labour/कृषि श्रमिक .... 2<br>Non-agri wage Labor/गैर कृषि श्रम मजदूरी .... 3<br>Business/व्यापार .... 4<br>Salaried/वेतन भोगी .... 5<br>Other (specify)________ 9<br>अन्य (स्पष्ट करें)	
111	What is the main occupation of your wife?<br>आपकी पत्नी का मुख्य व्यवसाय क्या है?	Cultivation/Family farm/खेती/परिवारिक खेती .... 1<br>Agricultural labour/कृषि श्रमिक .... 2<br>Non-agri wage Labor/गैर कृषि श्रम मजदूरी .... 3<br>Business/व्यापार .... 4<br>Salaried/वेतन भोगी .... 5<br>Not working/काम नहीं करते .... 6<br>Other (specify)/ अन्य (स्पष्ट करें)________ 9	
112	What is the main source of your household income?<br>आपके घर की मुख्य आमदनी कहाँ से आती है?	Own farm/अपनी जमीन पर खेती .... 1<br>Share Farm/बंटाई पर खेती .... 2<br>Daily Wage/ दहाड़ी मजदूरी .... 3<br>Business/निजी कारोबार .... 4<br>Salary/pension/(वेतन/पेंशन) .... 5<br>No regular work/नियमित काम नहीं .... 6<br>Other (specify)________ 9<br>अन्य (स्पष्ट करें)	

113	What is the average monthly income of your household?<br>आपके घर की औसतन मासिक आय क्या है?	Less than 1000/1000 से कम............1<br>1001-2000............2<br>2001-3000............3<br>3001-4000............4<br>4001 and above/4001 से ज्यादा............5<br>Don't want to share/बताना नहीं चाहते............6<br>No Regular income/नियमित आय नहीं............7<br>Don't Know/पता नहीं............8	
114	Agricultural Land (in acre)<br>कृषि योग्य भूमि (एंकड में)<br>**INS: Note local units and transfer it to acre**<br>**(स्थानिय युनिट में लिखें को एकड़ में बदल कर लिखें)**	Total/कुल ____________________ ☐☐<br>Irrigated/सिंचित______________ ☐☐<br>Non-irrigated/गैर–सिंचित ______ ☐☐	
114a	Does your household own/access any of the following?<br><br>क्या आपके घर में निम्नलिखित चीजें हैं/स्वामित्व में हैं?		

	Yes	No
Electricity at home/घर पर बिजली	1	2
TV (B&W)/टी.वी (ब्लैक एंड व्हाईट)	1	2
TV (color)/टी.वी (रंगीन)	1	2
Radio/रेडियो	1	2
Thresher/थ्रेसर	1	2
Tractor/ट्रेक्टर	1	2
Water pump/पानी का पंप	1	2
Cycle/साइकिल	1	2
Moped/Motorcycle/मोटरसाइकिल	1	2
Telephone/Mobile/फोन/मोबाइल	1	2
Electric fan/बिजली का पंखा	1	2
Sewing machine/सिलाई मशीन	1	2

EXPOSURE TO MASS MEDIA

115 Do you watch TV or listen to radio or read Newspaper?
क्या आप टी.वी देखते है या रेडियो सुनते है या समाचार पत्र पढ़ते है?

Please ask one by one.
कृपया एक–एक कर पूछे

If Yes, in an average week how many days out of 7 do you watch TV or listen Radio or read Newspaper?
(Please tick as appropriate)

यदि हॉ, तो औसतन सप्ताह के सात दिनों में आप टी. वी. कितने दिन देखते है? रेडियो कितने दिन सुनते है ? अखबार कितने दिन पढ़ते है?
(सभी उपयुत्त उत्तरो पर निशान लगायें)

	**Exposure**		**Frequency of exposure**			
	**Yes**	**No**	**All 7 Days**	**5-6 days**	**3-4 days**	**Not regular**
TV/टीवी	1	2	1	2	3	4
Radio/ रेडियों	1	2	1	2	3	4
Newspaper /अखवार	1	2	1	2	3	4

CMS social

HEALTH SEEKING BEHAVIOUR स्वास्थ्य के प्रति व्यवहार			
121	Where do you go to treat general ailments for your household members (children, parents etc.)?<br>आप अपने घर के सदस्यों (बच्चे, माता–पिता आदि) की सामान्य बिमारी के उपचार के लिये कहाँ जाते है?<br><br>**MULTIPLE RESPONSES POSSIBLE**<br>**(एक से अधिक उत्तर सम्भव)**<br><br>**WRITE DOWN THE NAME IF CAN'T IDENTIFY THE SITE TYPE**<br>**यदि स्वास्थ्य सुविधा को पहचान न सके तो कृपया नाम लिखें**	**Public Medical Sector**<br>Govt./Urban Hospital (DH/SDH)/<br>सरकारी / शहरी अस्पताल (डीएच / एसडीएच)...A<br>CHC/Rural Hospital/PHC/<br>सामुदायिक चिकित्सा केन्द्र / ग्राम अस्पताल / प्राथमिक चिकित्सा केन्द्र....B<br>Sub-Centre/सब सेन्टर....C<br>Nurse / ANM/नर्स / एएनएम....D<br>Other Public Sector Health Facility (Specify)/<br>अन्य सार्वजनिक स्वास्थ्य सुविधा (स्पष्ट करे)___E<br><br>**NGO Sector**<br>NGO/Trust Hospital/Clinic/गैर–सरकारी संस्था / ट्रस्ट अस्पताल / क्लिनिक....F<br>NGO Worker/ NGO कार्यकर्ता....G<br>Sahiyaa didi/ASHA/सहिया दीदी / आशा....H<br><br>**PVT Sector**<br>Private hospital/Clinic/Nursing home<br>प्राइवेट अस्पताल / क्लिनिक / निजी अस्पताल...I<br>Private Doctor/प्राइवेट डाक्टर....J<br>Pharmacy/Drugstore/दवा की दुकान....K<br>Vaidya/Hakim/Homeopath/<br>वैद्य / हकीम / होम्योपेथ....L<br>Traditional Healer (*Ojha*)/ओझा....M<br>Quack/unqualified village doctor/<br>नीम हकीम / अयोग्य ग्राम डाक्टर....N<br>Other Private Sector Health Facility (Specify)/<br>अन्य सार्वजनिक स्वास्थ्य सुविधा ________O<br>Don't Know/पता नही....P<br><br>Other (specify)__________X<br>अन्य (स्पष्ट करे)	

			ANC	Personal
122	Where does your wife mostly go to consult/treat her personal health problems, more specifically for the events like:<br>आपकी पत्नी अपने स्वास्थ्य की समास्याओं की सलाह एंव ईलाज के लिये कहाँ गई खास तौर पर निम्नलिखित परिस्थितियों में:<br><br>a. Antenatal care (ANC)<br>प्रसव पूर्व देखभाल<br><br>b. Personal health problem (white discharge, RTI, Menstruation)<br>निजी स्वास्थ्य समस्याएँ (जैसे सफेद पानी निकलना, प्रजनन मार्ग संक्रमण (RTI), माहवारी संबंधी)<br><br>**INS: ASK ABOUT THEIR LAST VISIT**<br><br>**निर्देशः अन्तिम बार कब गई इसके संबध में पूछें**	Govt./Urban Hospital (DH/SDH)/ सरकारी / शहरी अस्पताल (डीएच / एसडीएच)	11	11
		CHC/Rural Hospital/PHC/ सामुदायिक चिकित्सा केन्द्र / ग्राम अस्पताल / प्राथमिक चिकित्सा केन्द्र	12	12
		Sub-Centre/सब सेन्टर	13	13
		Other Public Sector Health Facility/ अन्य सार्वजनिक स्वास्थ्य सुविधा	14	14
		Nurse / ANM/नर्स / एएनएम	15	15
		NGO/Trust Hospital/Clinic/ गैर–सरकारी संस्था / ट्रस्ट अस्पताल / क्लिनिक	21	21
		NGO Worker/ NGO कार्यकता	22	22
		Sahiyaa didi/ASHA/ सहिया दीदी / आशा	23	23
		Dai /TBA /प्रशिक्षित / ट्रेंड दाई	24	24
		Pvt. hospital/Clinic/Nursing Home/ प्राइवेट अस्पताल / क्लिनिक / निजी अस्पताल	31	31
		Private Doctor/प्राइवेट डाक्टर	32	32
		Pharmacy/Drugstore/दवा की दुकान	33	33
		Vaidya/Hakim/Homeopath/ वैद्य / हकीम / अयोग्य ग्राम डाक्टर	34	34
		Traditional Healer (*Ojha*) /ओझा	35	35
		Quack/ unqualified village doctor/ नीम हकीम / अयोग्य ग्राम डाक्टर	36	36
		Other Private Sector Health Facility/ अन्य सार्वजनिक स्वास्थ्य सुविधा	37	37
		Don't Know / Never Tried/ पता नहीं / कोशिश नही की	98	98
		Other (specify) ________________ अन्य (स्पष्ट करें)	99	99
123	What is the location of the health facility (mentioned in Q122)? Is it located in this village or other village or in any nearby town?<br>स्वास्थ्य सेवा (प्रश्न 122 मे बताई गई) कहॉ पर स्थित है। यह आपके गॉव में ही है या दूसरे गॉव में है या करीब के शहर में है?	Own village/अपना गांव…………….<br>Other Village/अन्य गांव…..……….<br>Town/शहर…..……………………...<br>Don't know/Can't say/ पता नहीं / कह नही सकते…………	1<br>2<br>3<br>8	1<br>2<br>3<br>8

Now, I would like to ask you some questions regarding the decision making in your household
अब में आपसे कुछ प्रश्न आपके परिवार के निर्णय लेने संबंधी विषय के बारे में पूछना चाहूँगा

124	Please tell me, who makes the following decisions in your household.<br>कृपया बताएं कि निम्नलिखित मे आपके परिवार में निर्णय कौन लेता है	1=Respondent/स्वंय<br>2= Wife/ पत्नी<br>3= Jointly with wife/ पत्नी के साथ<br>4=Others in household/घर के अन्य सदस्य<br>5= Jointly with others in household/ घर के अन्य सदस्यों के साथ मिलकर					
	a. Obtaining Health care for your wife?<br>आपकी पत्नी के स्वास्थ्य देखभाल के लिए?	1	2	3	4	5	
	b. Choosing any particular doctor?<br>किसी चिकित्सक को चुनने के लिए?	1	2	3	4	5	
	c. What items to cook?<br>खाने में क्या बने?	1	2	3	4	5	
	d. Purchasing major household items (land, Jewelry)?<br>घर की मुख्य वस्तुए (जमीन, गहने) खरीदने के संबध मे?	1	2	3	4	5	

SECTION-2: REPRODUCTIVE HISTORY

Now I would like to ask you some questions about reproductive experiences of your wife. I know that some of these questions may embarrass you or make you feel uncomfortable, but your response will be kept confidential and help us to improve issues for women like your wife.
अब मै आपकी पत्नी के प्रजनन के अनुभव के बारे में आपसे कुछ प्रश्न पूछना चाहूँगा। हो सकता है कुछ प्रश्नो का जबाव देने में आपको संकोच हो लेकिन आपके द्वारा दिये गये जबाव गुप्त रखे जायेंगे और महिलाओं की समस्याओं में सुधार लाने में हमें सहायता देगें।

Q.No	Questions and filters	Coding Categories	Skip to
201	Now I would like to ask you about all the pregnancies your wife* have had during her life. Has she ever been pregnant?<br>अब में आपसे आपकी पत्नी* अब तक कितनी बार गर्भवती हुई है के बारे में पूछना चाहूँगा? क्या कभी वो गर्भवती हुई है?<br>***: Current wife in case of multiple marriage**<br>***:वर्तमान पत्नी के बारे मे पूछें यदि एक से ज्यादा बार विवाहित हो**	Yes/हाँ……………………………………….1<br>No/नहीं……………………………………..2	 → **212**
202	How many times she became pregnant (include all pregnancies)? वे कितनी बार गर्भवती हुई। (सभी गर्भधारण को मिलाकर)?	**Total number of pregnancies** [ ][ ]<br>Not sure/Can't say/पता नही………….98	
203	How many live births did she have (male, female)?<br>अब तक उन्होंने कितने बच्चों (जीवित) को जन्म दिया (लडका / लडकी)?	Male ………………… [ ]<br>Female ………………… [ ]<br>Total ………………… . [ ]	
204	Among them how many are alive now?<br>उनमे से अभी तक कितने जीवित है?	Male ………… [ ]<br>Female……… [ ]	
205	Has she ever had a pregnancy that ended in a still birth?<br>क्या उन्होने कभी मृत बच्चे को भी जन्म दिया है?	Yes/हाँ……………………………………1<br>No/नहीं …………………………………..2<br>Don't know/पता नहीं……………………..8	 → **207**<br>→ **207**

206	How many still births did she have?<br>कितने मृत बच्चों को उन्होने जन्म दिया?	**Number of still births** …… …. . ☐	
207	Has she ever had a pregnancy that ended in a miscarriage (spontaneous abortion)?<br>क्या वो कभी गर्भवती हुई है जिसमें उनका स्वतः(अपने आप) गर्भपात हुआ हो?	Yes/हाँ……………………………………1<br>No/नहीं ……………………………………2<br>Don't know/पता नहीं……………………8	 → **209**<br>→ **209**
208	How many such miscarriages happened?<br>ऐसे कितने गर्भपात हुऐ है?	**Number of miscarriages** ☐	
209	Has she ever aborted a pregnancy (induced abortion)?<br>क्या उन्होने कभी गर्भपात कराया है?	Yes/हाँ……………………………………1<br>No/नहीं ……………………………………2<br>Don't know/पता नहीं……………………8	 → **212**<br>→ **212**
210	How many such induced abortions happened?<br>कितनी बार ऐसे गर्भपात कराया है?	**Number of induced abortions** ☐	
211	When did she have her last induced abortion?<br>उन्होने अन्तिम गर्भपात कब कराया?	☐☐ ☐☐<br>**m m** **y y**	
212	In addition to the details of pregnancies mentioned above, has there been any occasion during the past three years when her menstruation was delayed?<br>उपर बताये गये गर्भधारण के संबध में दिये विवरण के अतिरिक्त, क्या **पिछले तीन सालों** में कभी ऐसा समय आया जब उनका मासिक देर से हुआ है?	Yes/हाँ……………………………………1<br>No/नहीं ……………………………………2<br>Don't know/पता नहीं……………………8	 → **215**<br>→ **215**
213	Did she do something to resume her menstruation, as it was not in time?<br>क्या उन्होने कोई उपाय किये जिससे उनका मासिक समय पर हो?	Yes/हाँ……………………………………1<br>No/नहीं ……………………………………2<br>Don't know/पता नहीं……………………8	 → **215**<br>→ **215**
214	If yes, What did she do? यदि हॉ, तो उन्होने क्या किया?<br>**(Open ended)**<br><br>**(INS FOR SUPERVISORS: IF YOU FEEL IT IS INDUCED ABORTION, GO BACK TO Q209 AND CORRECT IT)**<br>**(यदि आपको लगता हैं कि स्वयं कराया गया गर्भपात है, तो प्रश्न 209 में जाकर इसे सही करे।)**	________________<br>________________<br>________________	
215	Is your wife currently pregnant?<br>क्या आपकी पत्नी वर्तमान में गर्भवती है?	Yes/हाँ……………………………………1<br>No/नहीं ……………………………………2	**Check Box 2 & Box 3**

CHECK BOX NO. 2

Check Q203, Q206, Q208 Q210 & Q215. Sum of all these should be equal to Q202.
Cross-check and make correction.
Check Q215=1 (Currently Pregnant)→ Check Box No: 3

Q203, Q206, Q208 Q210 और **Q215.** का जोड़ **Q202** के बराबर होना चाहिए।
यादि **Q215=1** महिला वर्तमान में गर्भवती हो तो→ **Check Box No: 3**

216	Are you or your wife currently doing something or using any method to delay or avoid getting pregnant?<br>क्या आप या आपकी पत्नी ने गर्भ ना ठहरने या गर्भ में अन्तराल के लिये वर्तमान समय में कोई उपाय/तरीका अपनाया है?	Yes/हाँ……………………………………1<br>No/नहीं ……………………………………2	→ **218**

217	Why are you or your wife currently not using any methods to delay or avoid pregnancy?<br>आप या आपकी पत्नी ने वर्तमान समय मे गर्भ से बचने या इसमे अन्तराल रखने के लिये कोई उपाय क्यों नही अपना रहे है?<br><br>**INS: MULTIPLE RESPONSES POSSIBLE.**<br>**निर्देशः एक से अधिक उत्तर संभव है**<br><br>**PROBE: Any other reason?**<br>**पूछें: कोई अन्य कारण?**	**Fertility related reasons**<br>Want more children/ज्यादा बच्चे चाहते है....A<br>Not having sex/infrequent sex/wife away/संबंध नहीं बना रहे/कभी–कभी संबंध/पत्नी बाहर है......................................B<br>Menopausal/had hysterectomy/ महावारी बंद हो जाना/बच्चादानी निकाल दी…..…...…..C<br>Sub fecund/infecund/बाँझपन……......……..D<br>**Opposition to use**<br>Opposed to family planning/परिवार नियोजन के विरूद्व…………..……………….………..E<br>Wife opposed/ पत्नी का विरोध……..........F<br>Other family members/friends opposed/ अन्य सदस्यों/मित्रो का विरोध……….......G<br>Against religion/धर्म के विरूद्व…....……..…H<br>**Access/Knowledge**<br>Knows no method/तरीका मालूम नहीं……....I<br>Knows no source/ श्रोत मालूम नहीं...…....…J<br>Hard to get method/provider too far away/ clinic hours restrictive/मिलने में कठिनाई/सेवाप्रदाता का दूर होना/क्लिनिक का समय सीमित है………………...……..K<br>**Method related reasons**<br>Health concerns/स्वास्थ्य की चिन्ता…..……L<br>Fears side effects/नाकारात्मक प्रभाव….........M<br>Fears insertion/procedure/treatment/ विधि अपनाने में डर……………………….....N<br>Not effective/प्रभावशाली नहीं…..……...…..O<br>Inconvenient/असुविधाजनक….……………..P<br>Costs too much/अधिक मँहगा……….…..……Q<br>Don't like existing methods/तरीके मुझे पसंद नहीं…………………………………..………..R<br>Embarrassed/शर्म आती है…………..…..…...S<br>**Other reasons**<br>Other (specify)______________________X<br>अन्य (स्पष्ट करें)<br>Do not know/पता नही….……..…..……… Y	**If any response Goto Check Box 3**
218	Which method are you or your wife currently using?<br>आप या आपकी पत्नी वर्तमान मे कौन सा तरीका प्रयोग कर रहे है?	Female sterilization/महिला नसबंदी.……….A<br>Male sterilization/पुरूष नसबंदी.……..……B<br>IUD/Cu-T/आई यू डी/कापर टी…….…….C<br>Pill/गोली…………………………….……….……D<br>Condom/Nirodh/कंडोम/निरोध…………..…E<br>Injectables/DMPA/ गर्भ–निरोधक इंजेक्शन/डीएमपीए..……..…F<br>Rhythm/Safe period/सुरक्षित काल पद्वित….G<br>Withdrawal/अर्धपतन यानि विदद्रावल……..H<br>Others (Specify) ____________________ X<br>अन्य (स्पष्ट करें)	

219	Since how long have you or your wife been using the method continuously?<br>आप या आपकी पत्नी कब से इसका नियमित रूप से प्रयोग कर रहे है?	months ☐☐<br>8 years or longer……………………….96<br>आठ वर्ष से ज्यादा	

CHECK BOX NO. 3
Check Q209, Q211 & Q214: Wife had an induced Abortion during last 3 years? (August 2007 to July 2010)
Yes=1 → Q301 No=2 → Q401 [Section 4]
क्या पिछले 3 साल के दौरान पत्नी ने कोई गर्भपात करवाया है?

SECTION 3: ABORTION PREVELANCE AND EXPERIENCE (IN LAST 3 YEARS)

Now I would like to ask you some questions about abortion experience of your wife. Please remember that this interviews confidential. Your answers will be very helpful in improving abortion services in communities like this. I want to talk only about the **last** abortion that your wife had.

अब मै आपकी पत्नी के गर्भपात के अनुभव के बारे में आपसे कुछ प्रश्न पूछना चाहूँगा। कृपया ध्यान दे यह साक्षात्कार गोपनीय है। आपके जबाव आपके समुदाय में गर्भपात की सेवाओं में सुधार लाने के लिए बहुत मददगार होगें। मै सिर्फ आप से आपकी पत्नी के अंतिम गर्भपात के बारे में पूछना चाहूँगा।

Q.No	Questions and filters	Coding Categories		Skip to
301	Why did she have this abortion (TALK ABOUT LAST ABORTION ONLY)?<br><br>उन्होने यह गर्भपात क्यों करवाया? (सिर्फ आखरी गर्भपात के विषय में बात करे)<br><br>**Any other reason?**<br>**अन्य कोई कारण?**<br><br>**MULTIPLE RESPONSES POSSIBLE**<br>**एक से अधिक उत्तर संभव है**	Did not want another child then/ last child was too young/और बच्चा नहीं चाहते/अखिरी बच्चा बहुत छोटा है……...	A	
		Did not want a child at all/ have enough/बच्चा नहीं चाहते/काफी है……………………..….....	B	
		Contraceptive Failed/गर्भ–निरोधक का असर नहीं हुआ……………..…………………………	C	
		I did not want the child/ मै बच्चा नहीं चाहता था……………………………….……………..	D	
		My mother did not want the child/मेरी माँ बच्चा नहीं चाहती थी……………………..……….	E	
		Could not afford another child/poverty/और बच्चे का पालन–पोषण नहीं कर सकते/गरीबी…..…..	F	
		Pregnancy was a result of rape/गर्भधारण बलात्कार द्वारा हुआ……………..………………	G	
		Health problems/स्वास्थ्य संबंधी समस्या….……..	H	
		Fetus had congenital defects/भ्रूण में पैदायशी दोष……………………………………………..	I	
		Fetus was female/भ्रूण का लड़की होना…..…….	J	
		Fetus was male/भ्रूण का लड़का होना…………	K	
		Other (specify)___________________________<br>अन्य (स्पष्ट करें)	X	

302	Who all were involved in taking decision of her last abortion?<br>उनके आखिरी गर्भपात से संबधित निर्णय लेने में कौन कौन सम्मिलित थे?<br>Anyone else?<br>अन्य कोई?<br>**[MULTIPLE RESPONSES POSSIBLE]**<br>**(एक से अधिक उत्तर संभव है)**	Wife / पत्नी ....……………………………….……A<br>Myself/स्वय..……………………………...……B<br>Parents-in-law/सास ससुर……...……..……..……….C<br>Parents/माता–पिता…..……………………..…..….D<br>Other relatives/अन्य रिश्तेदार…..……….……..……..E<br>Other (specify)______________________________X<br>अन्य (स्पष्ट करें)	
302A	Who was the main decision maker?<br>मुख्य रूप से इसका निर्णय किसने लिया था?<br><br>**[SINGLE RESPONSE ONLY]**<br>**(एक ही उत्तर)**	Wife / पत्नी ....…………………………………...…1<br>Myself/स्वयं..……………………………….……...2<br>Mother-in-law/सास ……………………..……..…..3<br>Father-in-law/ससुर…...……………………………4<br>Parents/माता–पिता……………..……………….......5<br>Other relatives/अन्य रिश्तेदार …………………………6<br>Other(specify)________________________________9<br>अन्य (स्पष्ट करें)	→ 303
302B	Did you have agreement in this decision?<br>क्या आप इस गर्भपात के लिए सहमत थे ?	Yes/हाँ…...……………………………………………1<br>No/नहीं……………………………………………..…..2<br>Other<br>(specify)__________________________________9<br>अन्य (स्पष्ट करें)	
303	Where did she perform this (last) abortion? I mean at home or health facility/clinic or both at home and health facility?<br>उन्होने अंतिम गर्भपात कहाँ करवाया?अर्थात् क्या उन्होने अपना गर्भपात घर मे **या** क्लिनिक/अस्पताल मे कराया **या** घर मे और क्लिनिक/अस्पताल दोनो जगह कराया?	At Home/घर मे……………………………...…...……...1<br>At clinic/hospital/क्लिनिक/अस्पताल मे.........................2<br>Both at home and clinic/घर और क्लिनिक दोनो में…...3	→ 304<br>→ 308<br>→ 303A
303A	You just mentioned that your wife performed abortion both at home and clinic; can you please explain this process in little detail? Did she first attempt performing abortion at home and then visited a clinic? Or, she visited a clinic (doctor, chemist) first and performed abortion at home.<br><br>**NOTE DOWN THE STEPS** (क्रम से लिखें)<br><br>अभी आपने बताया किं आपकी पत्नी ने गर्भपात घर मे और क्लिनिक/अस्पताल दोनो जगह कराया। क्या आप इस बारे मे कुछ विस्तार मे बता सकते है? क्या पहले उन्होने गर्भपात घर में कराने की कोशिश की, फिर क्लिनिक गई थी या पहले आप क्लिनिक गई और बाद में घर पर गर्भपात कराया?	(see steps table below)	

**Steps**	**At home / Clinic**
1st	
2nd	
3rd	
4th	

304	If at home, who assisted her to perform her last abortion?<br><br>यदि अंतिम गर्भपात घर पर कराया, तो इसमें उनकी सहायता किसने की?	Mother-in-Law/सास………………………………..…A<br>Sister/बहन…………………………………………….B<br>Sister-in-Law/नंनद……………………………….……C<br>Mother/माँ……………………………………………..D<br>Friend/दोस्त……………………………………….....E<br>Myself / स्वय……………..…………….…………..……F<br>Any other(specify) ____________________________X<br>कोई अन्य (स्पष्ट करें)	
305	You just mentioned that you (your wife/ relative/friend) tried to perform the abortion.<br><br>जैसा कि आपने कहा कि आप/ आपकी पत्नी /रिश्तेदार/आपके मित्र ने गर्भपात की कोशिश की<br><br>What did you (or that person) do?<br>आपने/उस व्यक्ति ने यह कैसे किया?	Took home made concoction (Kahva, papaya, chilli)<br>घरेलू विधि से (काहवा/पपीता/मिर्च)………………....1<br>Took goli/tablets/गोलियाँ लीं……………………..….....2<br>Ayurvedic remedies/आयुर्वेदिक दवाई ली…………….....3<br>Inserted herbs/stick/जड़ी–बूटी को अंदर डाला………..4<br>Inserted instruments/कोई उपकरण अंदर डालकर…….5<br>External massage/बाहरी मालिश………………………6<br>Any other (Specify)____________________________7<br>कोई अन्य (उल्लेख करें)<br>Don't know/can't say/पता नही/कह नही सकते…..…8	
306	Did this action enable her to terminate the pregnancy completely?<br>क्या उस विधि को अपनाने से आपकी पत्नी का पूर्ण गर्भपात हो पाया?	Yes/ हाँ………………………..……………….…….1<br>No, it was incomplete/नहीं, अधूरा रह गया……..…....2<br>No, nothing has happened/नहीं,कुछ भी नहीं हुआ........3<br>I am not sure/ मुझे ठीक से नहीं पता………………....4<br>Other (specify)_____________________________9<br>कोई अन्य (उल्लेख करें)	
307	Did she experience any complication after this self tried abortion?<br>स्वयं गर्भपात करने से क्या उनको कुछ जटिलताओं/समस्याओं का सामना करना पड़ा?	Yes/हाँ………………………..……………..……1<br>No/नहीं……………………………….……………..2<br>Other (specify)______________________________9<br>अन्य (स्पष्ट करें)	 → 308
307A	What kind of abortion related complication(s) did she have?<br><br>उनको गर्भपात से सम्बंधित किस–किस तरह की जटिलता/समस्या आयी?<br><br>**MULTIPLE RESPONSES POSSIBLE.**<br>**एक से अधिक उत्तर संभव है**<br><br>**Probe: Any other problems/ complications?**<br>**पूछें: अन्य कोई जटिलता/ समस्या?**	Immediate heavy bleeding/ तुरंत ज्यादा रक्तस्राव.….…….A<br>Persistent bleeding……………………………..….B<br>(for 2 to 4 weeks post-abortion)<br>लगातार रक्तस्राव (गर्भपात के पश्चात 2 से 4 सप्ताह तक)<br>Persistent bleeding……………………….………….C<br>(for more than 4 weeks post-abortion)<br>लगातार रक्तस्राव (गर्भपात के पश्चात 4 सप्ताह से ज्यादा)<br>Backache/ कमरदर्द………………………….……….D<br>Abdominal pain/cramps/ पेट दर्द …………………..…….E<br>Nausea/vomiting/ बेहोशी/उल्टी होना ………………...…..F<br>Foul smelling discharge/ दुर्गंधयुक्त स्राव ……………..….G<br>High-grade fever/तेज बुखार ….........……………………H<br>Fatiuge and weakness/ थकावट और कमजोरी ……….….I<br>Fainting spells/ वेहोशी जैसा लगना....................................J<br>Uterus perforation/rapture/ बच्चेदानी मे छेद……………..K<br>Irregular menstruation/ अनियमीत महावारी ……..…......…L<br>Infertility/sterility/ बच्चा न होना…….……………………..M<br>Psychological symptoms: sadness, guilt, sleep, disturbance……………………………………………N<br>मनोवैज्ञानिक लक्षण: दु:खी, आत्मग्लानी, नींद, झिझक<br>Other (specify) __________________________X	

308	Did your wife go or consult any doctor or health worker for her abortion? By health worker we mean doctor, nurse, dai, anganwadi workers, Sahiyya/ASHA, Chemist etc. क्या आपकी पत्नी चिकित्सक / स्वास्थ्यकर्त्ता के पास गर्भपात के लिए या सलाह लेने गयी थी? स्वास्थ्यकर्त्ता का मतलब चिकित्सक, नर्स, दाई, आंगनवाड़ी दीदी, सहिया / आशा प्रशिक्षित दवा विक्रेता, आदि	Yes/हाँ……………………………..………….……1<br>No/नहीं……………………………….…………….….2<br>Don't know/पता नहीं……………………...….……….8	 → **338**<br>→ **338**
309	How many providers did she go for your abortion? For example how many doctors, nurse, Sahiyya, chemist/pharmacist, dais etc. आपकी पत्नी अपने गर्भपात के लिए कितने सेवाप्रदाता के पास गई?	No of providers ……………………. ☐<br>Don't know/cant's say/पता नहीं / कह नही सकते……8	
310	How many times did she visit this/these provider/s? I mean to ask, to whom did she consult in her first visit and then next and so on? आपकी पत्नी इन सेवाप्रदाताओं के पास कितनी बार गयी? अर्थात् गर्भपात या सलाह के लिए सवप्रथम किस सेवाप्रदाता के पास गयी उसके बाद क्रमशः किन सेवाप्रदाताओ के पास गयी थी<br>**Please tell me in sequence of your visits.**<br>**कृपया मुझे क्रमानुसार बताऐं कितने बार गई**<br>**INS: IT MIGHT BE ONE PROVIDER & MANY VISITS**<br>**एक सेवा प्रदाता के पास कई बार गये हो सकते हैं।**<br>**INS: IT MIGHT BE MANY PROVIDERS MANY VISITS**<br>**कई सेवा प्रदाता के पास कई बार गये हो सकते हैं।**	(see table below)	

Visit/ मुलाकात #	Name (if known)	Type of Provider/s
1st Visit/ मुलाकात		
2nd Visit/ मुलाकात		
3rd Visit/ मुलाकात		
4th Visit/ मुलाकात		

310A	How did you wife come to know that she can go to the provider for abortion?<br><br>आपकी पत्नी को कैसे पता चला कि वह सेवाप्रदाता के पास गर्भपात के लिए जा सकती हैं?	ANM suggested ए एन एम ने सलाह दी ……………...1<br>ASHA/SAHIYA sugested सहिया दीदी / आशा ने सलाह दी ……………………………………………………..2<br>Anganwadi Worker (AWW) suggested आंगनवाड़ी कार्यकर्ता ने सलाह दी……………………..……………3<br>Trained Birth Attendent (TBA) suggested प्रशिक्षित दाई ने सलाह दी …………………………….….………..…..4<br>Any other health worker suggested अन्य स्वास्थयकर्ता ने सलाह दी………………………………………………5<br>NGO Worker/ NGO कार्यकर्ता ……………………...….6<br>Wife shared पत्नी ने बताया ………..…………….…….7<br>Family members/Friends shared परिवार के सदस्य / मित्र ने बताया…………………………………………..……...8<br>Wall Sign दिवार लेखन द्वारा…………………….………..9<br>Street Drama नुक्कड़ नाटक…………………….……...10<br>Other (Specify) अन्य (स्पष्ट करें)_______________ 11	

Now, I would like to ask you about experiences of your wife with the provider(s) she went to for her last abortion. You told me that she visited ___ times (see 310: number of visits). Please tell me about her experience with that/each visits, starting with the 1st visit.
अब में आपसे आपकी पत्नी के आखरी गर्भपात में (310 के उत्तर को लिखें) सेवाप्रदाता के साथ उनके अनुभव के बारे में कुछ प्रश्न पूछना चाहूंगा, आपने बताया कि आपकी पत्नी (310 के उत्तर को लिखें)............. के पास (310 के उत्तर को लिखें) .... बार गयी, कृपया पहली बार जब गई तब से शुरू करके उनके हरेक अनुभव को बतायें।

Q. No.	Questions	Coding Categories	1st Visit<br>_______<br>_______	2nd visit<br>_______<br>_______	3rd visit<br>_______<br>_______
311	**INS: CHECK THE NAME OR TYPE OF PROVIDER UNDER EACH VISIT**<br>**निर्देशः प्रत्येक मुलाकात के दोरान स्वास्थ्यकर्त्ता का नाम और प्रकार जॉच लें**			Same as 1st →318<br>Different 3 →312	Same as 1st 1→318<br>Same as 2nd 2→318<br>Different 3→ 312
312	Who was the provider she went for the last abortion?<br>आपकी पत्नी अपना आखिरी गर्भपात करवाने के लिए जिनके पास गई थी वो सेवाप्रदाता कौन थे ?	Doctor- Govt./ District hospital Khunti /डॉक्टर–सरकारी ज़िला अस्पताल खुंटी	1	1	1
		Doctor- Govt. Rural hospital Silli / डॉ–सरकारी ग्रामीण अस्पताल सिल्ली	2	2	2
		Other Govt. hospital PHC/CHC______ अन्य सरकारी अस्पताल/ सामुदायिक स्वास्थ्य केंद/प्राथमिक स्वास्थ्य केंद्र	3	3	3
	**INS: ASK EACH QUESTION (312-319) BY VISIT**<br>**निर्देशः प्रत्येक सवाल (312-319) मुलाकात के अनुसार पुछे**	Nurse/ANM/ नर्स/ए०एन०एम	4	4	4
		Other staff-Govt.Hospital/centre/ अन्य सेवादाता–सरकारी अस्पताल	5	5	5
		Doctor-Private Clinic/Ipas Provider ______________________________ डॉक्टर–प्राईवेट/क्लीनिक Ipas प्रवाईडर	6	6	6
	**INS: IN CASE OF ONE (ONLY) PROVIDER, DON'T REPEAT (312-319) निर्देशः यदि एक ही सेवाप्रदाता है तो सवाल (312-319) की पुनरावत्ती न करें।**	Other Private Doctor /Clinic/ Nurshing Home _________________________ अन्य डॉक्टर–प्राईवेट/क्लीनिक/ नर्सिंग होम	7	7	7
		Other Staff Nursging home/ अन्य सेवादाता–नर्सिंग होम	8	8	8
		Chemist/pharmacist/medical shop/ दवा विक्रेता	9	9	9
		RMP/Jholachap/quack/आर०एम०पी	10	10	10
		Dai/ दाई	11	11	11
		AWW/ आंगनवाड़ी दीदी	12	12	12
		Other(specify)/ ___________________ अन्य (उल्लेख करें)	13	13	13
		Don't know/Can't say/ पता नहीं/कह नही सकते	88	88	88
313	What was the location of the provider? वह सेवाप्रदाता किस जगह स्थित है?		**1st Visit**<br>________<br>________	**2nd visit**<br>________<br>________	**3rd visit**<br>________<br>________
		Same village/ उसी गाँव/शहर	1	1	1
	**Write down the name of location**<br>**स्थान का नाम लिखें**	Other town/अन्य शहर	2	2	2
		Other village/ अन्य गाँव	3	3	3

314	How far did she travel to reach this provider?<br>आपकी पत्नी को सेवाप्रदाता तक पहुंचने में कितनी पूरी तय करनी पड़ी?	Distance दूरी (in K.M.)<br>**Put 00 in case of same village**<br>उसी गाँव के लिए 00 लिखें।	________	________	________
315	Who made the final decision to go to the provider?<br>सेवाप्रदाता के पास जाने का अन्तिम निर्णय किसका था?	Self/स्वंय	A	A	A
		Wife/ पत्नी	B	B	B
		Jointly with Family/<br>परिवार के सदस्यों के साथ	C	C	C
		Mother –In- Law/सास	D	D	D
		Mother/माँ	E	E	E
		Other (specify)/अन्य (स्पष्ट)__________________	X	X	X
316	Why did she choose this particular provider<br>उन्होने इस सेवाप्रदाता को क्यों चुना?	____________________<br>____________________<br>____________________<br>____________________	____________________<br>____________________<br>____________________<br>____________________	____________________<br>____________________<br>____________________<br>____________________	
317	How did she learn of the provider?<br>इस सेवा देने वाले के बारे में उनको कैसे पता चला?	Myself/मेरे द्वारा	A	A	A
		Family members/Friends/<br>परिवार के सदस्य/दोस्त	B	B	B
		Local provider/<br>स्थानीय सेवाप्रदाता	C	C	C
		ANM/एएनएम	D	D	D
		ASHA/SAHIYA/आशा/सहिया	E	E	E
		AWW/आगंनवाड़ी कार्यकर्ता	F	F	F
		TBA/प्रशिक्षित दाई	G	G	G
		Other Health Worker<br>____________________<br>अन्य स्वास्थ्यकर्ता	H	H	H
		NGO worker/ NGO कार्यकर्ता	I	I	I
		Wall sign /दीवार लेखन द्वारा	J	J	J
		Street Drama/नुक्कड़ नाटक	K	K	K
		Other (specify) /अन्य (स्पष्ट)__________________	X	X	X
318	What month of pregnancy did she visit the provider?<br>गर्भ के कौन से महिने के दौरान आपकी पत्नी सेवाप्रदाता के पास गयी?	Record in month<br>**महिनों मे लिखें**<br>Don't know/Can't say/<br>पता नहीं/कह नही सकते	________<br>8	________<br>8	________<br>8

319	What type of treatment/ consultancy did she receive at this visit?<br>इस मुलाकात में उन्होने किस प्रकार की चिकित्सा/ परामर्श प्राप्त किया?<br><br>**[MULTIPLE RESPONSES POSSIBLE]**<br>**(एक से अधिक उत्तर संभव है)**<br><br>**(INS: please note down services at this visit only)**<br><br>**"कृप्या उन्हीं सेवाओं को लिखें जो उनको सेवाप्रदाता से इस मुलाकात में प्राप्त हुई"**	Had internal examination/ अंदरूनी जाँच की	A	A	A
		Had pregnancy test/ गर्भ की जाँच की	B	B	B
		Discussed reasons of seeking abortion/ गर्भपात करवाने के कारणों के बारे में चर्चा की	C	C	C
		Had ultra sound/ अल्ट्रासाऊँड हुआ	D	D	D
		Had surgical abortion with general anesthesia/ ऐनेस्थीसिया के साथ औजारों के प्रयोग से गर्भपात किया	E	E	E
		Had surgical abortion without general anesthesia/ ऐनेस्थीसिया के बिना औजारों के प्रयोग से गर्भपात किया	F	F	F
		Got tablets for abortion/ गोली खाकर गर्भपात किया	G	G	G
		Injections/ सुई/इंजैक्शन से	H	H	H
		Given something for applying at home/ घर पर लगाने के लिए कोई पदार्थ दिया	I	I	I
		Inserted something into the vagina/ गुप्तांग/योनि के अंदर कुछ डाला	J	J	J
		Had heavy (oil) massage/ तेल से जोर से मालिश की	K	K	K
		Had abortion by_______________ से गर्भपात करवाया	L	L	L
		Treatment for abortion related complication / गर्भपात जटिलता के लिए इलाज	M	M	M
		Could not meet any doctor/refused to see/ चिकित्सक से मिल नहीं पाए/ मिलने से मना किया	N	N	N
		Referred to another doctor/hospital/ अन्य चिकित्सक/अस्पताल के पास भेजा	O	O	O
		Other (specify)_______________ अन्य (स्पष्ट)	X	X	X

CMS *social*

CHECK BOX NO. 4<br>CHECK Q319<br>**Wife had abortion in this visit?**<br>**इस मुलाकात में पत्नी ने गर्भपात कराया?**		Y:1→320<br>N:2→Next visit	Y:1→320<br>N:2→Next visit	Y:1→320<br>N:2→Next visit
320	Did doctor tell her the name of the abortion procedure?<br>क्या चिकित्सक ने उनको गर्भपात की विधि का नाम बताया था?	Yes.... 1<br>No... 2	Yes.... 1<br>No... 2	Yes.... 1<br>No... 2

321	Could you tell me what methods have been applied on her?<br>क्या आप बता सकते हैं कि आपकी पत्नी पर कौन सी विधि का इस्तेमाल किया था?<br>**INS: PROBE TO IDENTIFY THE METHOD AS THE**<br>**निर्देश: तरीके की पहचान के संबंध में पूछें**<br>MA: tablets or pills which can be taken orally or can be inserted into the vagina for abortion<br>MA: गर्भपात के लिए टेबलेट या गोलियां जो कि मुंह से खाई जा सकती है या फिर योनि के अन्दर डाली जा सकती है	1: Any surgical method with general anasthesia<br>जनरल एनसथेसिया के साथ कोई शल्य तरीका	1	1	1
		2: Any surgical method without general anasthesia<br>बिना जनरल एनसथेसिया के साथ कोई शल्य तरीका	2	2	2
		3. Medical Abortion (MA)<br>दवाइयों द्वारा गर्भपात	3	3	3
		4. Other (specify)__________<br>अन्य (स्पष्ट)	9	9	9
**INS: PLEASE ASK FOR ALL VISITS BEFORE GOING TO CHECK BOX NO.5**			**GO TO Next Visit**	**GO TO Next Visit**	**GO TO Next Visit**

CHECK BOX NO. 5

CHECK Q321: Wife had medical abortion ? **Yes=1→ Q322** **No =2→ Q328**

क्या पत्नी का गर्भपात गोलियो से हुआ था?

322	You just mentioned that her abortion has been carried out through tablets. How did she get these tablets?<br>आपने बताया कि आनकी पत्नी का गर्भपात टेबलेट/गोलियॉ के द्वारा हुआ। उनको ये टेबलेटस/गोलियाँ कैसे प्राप्त हुई?<br>**Would you say…….READ THE RESPONSE**<br>**क्या आप कहेंगें.........**	Doctor provided the tablets/चिकित्सक द्वारा……….......1<br>Doctor given prescription to buy tablets………………..2<br>चिकित्सक ने टेबलेट/गोलियॉ लेने के लिए लिखित विवरण दिया<br>Chemist provided without prescription………………………………..…..…..3<br>कैमिस्ट द्वारा बिना चिकित्सक के विवरण के<br>Other(specify) ________________4<br>अन्य (स्पष्ट)<br>Don't Know /Can't say/ पता नही/कह नही सकते…..8	→ **323**
322a	In total, how many times did she visit to her doctor or hospital for completing the procedure?<br>कुल मिलाकर, गर्भपात के लिए, आपकी पत्नी कितनी बार डाक्टर के पास या अस्पताल में गई?	____________ ☐	

323	Could you remember, in total, how many tablets did she take for this abortion?<br>क्या आप याद कर सकते है कि उन्होने कुल मिलाकर, इस गर्भपात के लिए कितनी टेबलेट/गोलियॉ खाईं?	1 tablet/1गोली...………………………….…...………1<br>2 tablets/2 गोली ……………………….…………....2<br>3 tablets/3 गोली ……………………………………….3<br>More than 3 tablets/ 2 से ज्यादा गोली….………...……4<br>Don't remember/याद नहीं…….………………………..8	
324	Did she take one particular type of tablet or two different types of tables?<br>क्या उन्होने एक ही प्रकार की टेबलेट/गोलियॉ ली थी या दो अलग–2 प्रकार की टेबलेट/गोलियॉ ली थी?	One type of tablet/एक तरह की गोली…………………1<br>Two different types of tablet/दो अलग तरह की गोली..2<br>More than two types of tablet/दो से ज्यादा तरह की गोली……………………………………………………3<br>Don't remember/Can't say/याद नही/कह नही सकते..8	
325	Would you be able to tell me the names of the tablets you have taken for this abortion?<br>क्या आप मुझे उन टेबलेटस/गोलियॉ के बारे में बता सकते है जो उन्होने इस गर्भपात के लिए इस्तेमाल की थी?<br><br>**MULTIPLE RESPONSE POSSIBLE**<br>**(एक से अधिक उत्तर संभव)**	Mefiprestone/MT Pill………………..……………..…A<br>Misoprostol…………………………...………….….B<br><br>Other(specify)______________________________ X<br>अन्य (स्पष्ट)<br>Don't Know /Can't say/पता नही/कह नही सकते……Y<br>**INS: PLEASE CHECK THE LIST OF MA BRANDS AVAILABLE IN THE MATKET**	
325a	Did she take these tablets at hospital / doctor's clinic or at home?<br><br>क्या उन्होने ये गोलियाँ अस्पताल में/डाक्टर के क्लिनिक में/घर मे ली?	All at clinic/ Hospital/हर बार क्लिनिक में/अस्पताल में...1<br>One at hospital & other at home/एक बार अस्पताल और अन्य बार घर में……………………………………… 2<br>All at chemist shop हर बार दवाई की दुकान में ………3<br>One at chemist shop & other at home/<br>एक बार दवाई की दुकान में और अन्य बार घर में ……4<br>All at Home/हर बार घर में …………………………... 5<br>Don't Know /Can't say/पता नही/कह नही सकते…….8<br>Other (Specify)/ ____________________________9<br>/अन्य (स्पष्ट)	
326	Did she take all tablets one at a time or in sequence? If in sequence, would you be able to tell me the sequence?<br>क्या उन्होने सभी टेबलेटस/गोलियॉ एक ही बार खाई थी या क्रम से खाई थी? यदि क्रम से ली थी तो क्या आप मुझे क्रम बता सकते है?<br>**PROBE FOR THE DAY OF CONSUMPTION.**<br>**उपभोग के दिन के बारे में पूछें**	All at once/एक साथ…….………………..………..1<br>One on the 1st day & then 2nd dose on 3rd -4th day……..2<br>1 दिन एक गोली,दूसरे दिन दूसरी, तीसरे दिन तीसरी और चौथे दिन चौथी<br>Other (specify)______________________________9<br>अन्य (स्पष्ट)<br>Don't Know /Can't say/पता नहीं/कह नही सकते……8	
326A	Who explained her about how to take the tablets?<br>उन्हें किसने बताया कि गोलियाँ कैसे लेनी है?	Doctor चिकित्सक …………………………….……..1<br>Chemist कैमिस्ट ……..…………………………… 2<br>ANM /एएनएम ……………………………………….3<br>ASHA / SAHIYA आशा/सहिया …………………..….4<br>TBA प्रशिक्षित दाई ……………………………………..5<br>Other (Specify) अन्य (स्पष्ट)____________________9<br>No body explained किसी ने नहीं बताया …………………7<br>Don't Know /Can't say/पता नही/कह नही सकते……8	
327	Did the provider discuss about the possible side effects of medication abortion to her?<br>क्या आपकी पत्नी को सेवाप्रदाता ने गोलियो से गर्भपात के दुष्परिणामों के बारे में बताया था?	Yes/हाँ…..………………………………………..……1<br>No/नहीं………………………………………………2<br>Don't Know /Can't say/पता नही/कह नही सकते……8	

328	Thinking of all the visits she made to the provider for this abortion, can you tell me approximately how much cost did you incur on the following events:<br>सभी मुलाकातों, को ध्यान में रखते हुए क्या आप बता सकते है कि इस गर्भपात के लिए सेवाप्रदाता के पास जाने मे इन सभी मुलाकातों पर लगभग कितना खर्चा आया<br><br>**INS: IN CASE RESPONEDENT IS UNABLE TO PROVIDE THE COST BREAKS, PROBE HER TO PROVIDE THE TOTAL APPROXIMATE COST.**<br>**निर्देशः यदि उत्तरदाता अलग–अलग खर्चे के बारे में नही बता सकता है तो उनसे पूरे खर्चे के बारे में पूछें**	Travel /Transport यातायात में: ☐☐☐<br>Fees/Consultation फीस में: ☐☐☐<br>Abortion procedure गर्भपात करवाने में: ☐☐☐<br>Tablets for abortion गर्भपात की गोलिया: ☐☐☐<br>Medicines दवाईयों में: ☐☐☐<br>Any test /examination अन्य जाँच में: ☐☐☐<br>Other(specify):_______ अन्य उल्लेख करें: ☐☐☐<br>**TOTAL COST(Rs.)**: ☐☐☐☐	
329	Did the provider discuss with her about different / any other methods of abortion?<br>क्या सेवाप्रदाता ने उनसे गर्भपात के दूसरे तरीको के बारे में भी बातचीत की?	Yes/हाँ……1<br>No/नहीं……2<br>Don't Know/Can't say/पता नहीं / कह नही सकते……8	
329a	Did you accompany your wife to the various providers?<br>क्या विभिन्न सेवाप्रदाताओ के पास जाने के समय आप अपनी पत्नी के साथ गये?	Yes, accompanied/ हाँ, साथ दिया……1<br>No/नहीं……2	→**330** (1)
329b	Who among these accompanied you to the various providers?<br>इनमें से कौन उनके साथ विभिन्न सेवाप्रदाताओं के पास गया है?	Went alone/ अकेले जाते है……1<br>Her Sister-in law/ ननद / भाभी / गोतनी……2<br>Her Mother-in law/ सास……3<br>Her Parents/माता–पिता……4<br>Friends/मित्र……5<br>Other (specify)/अन्य (स्पष्ट)……9	
	**POST ABORTION CONTRACEPTION गर्भपात के बाद गर्भ निरोधन**		
330	Did the provider talk to her about how to avoid getting pregnant again?<br>क्या सेवाप्रदाता ने उनसे दोबारा गर्भधारण को कैसे रोक सकते है के बारे में बातचीत की?	Yes/हाँ……1<br>No/नहीं……2<br>Don't know/Can't say/पता नहीं / कह नही सकते……8	 → **331** (2)<br>→ **331** (8)
330a	Who was that provider?<br>वह सेवाप्रदाता कौन था / थी?	______________________	
330b	In which visit, provider talked to her about post abortion contraception?<br>सेवाप्रदाता ने उनसे गर्भपात के बाद गर्भनिरोधक के बारे मे कब बताया?<br><br>**MULTIPLE RESPONSES POSSIBLE**<br>**एक से अधिक उत्तर संभव है**	1st Visit/ 1st मुलाकात…… 1<br>2nd Visit/ 2nd मुलाकात…… 2<br>3rd Visit/ 3rd मुलाकात…… 3	

331	Did you or your wife start using any contraception after your abortion? गर्भपात के बाद क्या आप या आपकी पत्नी ने किसी भी प्रकार के गर्भ निरोधक का इस्तेमाल शुरू किया?	Yes/हाँ……………………………………………………1<br>No/नहीं……………………………………………………2	→ 333
332	Why didn't you or your wife accept any family planning method to avoid another unwanted pregnancy after your abortion?<br><br>गर्भपात के बाद अनचाहे गर्भ को रोकने के लिए आप या आपकी पत्नी ने परिवार नियोजन का कोई तरीका क्यों नही अपनाया?	Wasn't counseled about FP ……………………………..….1<br>परिवार नियोजन के बारे मे किसी ने बताया नहीं<br>Didn't know any methods ………………………………….2<br>परिवार नियोजन के किसी विधि के बारे मे पता नहीं<br>Didn't know how to use any methods ……………………..3<br>परिवार नियोजन के विधि का प्रयोग कैसे करते है पता नहीं<br>Don't want to use FP methods ……………………..……..4<br>किसी विधि का प्रयोग नहीं करना चाहते है<br>Due to side effects ……………………………..…………5<br>दुष्प्रभाव के डर से<br>Wife did not want ………….……………………………..6<br>पत्नी की इच्छा नही<br>Wanted a child ………………………...…………………7<br>और बच्चे की चाहत<br>Any other reason (specify) ________________________ 9<br>काई अन्य कारण (स्पष्ट)	335
333	Which method(s) did you or your wife accept?<br>आप या आपकी पत्नी ने कौन सा तरीका अपनाया?<br>**INS: MULTIPLE RESPONSES POSSIBLE**<br>**निर्देशः एक से अधिक उत्तर संभव है**<br><br>**PROBE: Any other method?**<br>**पूछेंः अन्य कोई तरीका?**	Male sterilization/पुरूष नंसबदी…..……….…………A<br>Female sterilization/महिला नंसबदी……..……………B<br>Pills/गोली…………………………………….…………...C<br>IUD/आई यू डी…..……………………..…………...D<br>Condom/कांडोम……………………………….……..…E<br>Injectables/इंजेक्शन….……………………………………F<br>Rhythm/Periodic method/सुरक्षित काल पद्वति…..…....G<br>Withdrawal method/अर्धपतन यानि विदड्रावल...............H	
334	Did you or your wife accept that/those methods(s) because you wanted to or because it was a pre-condition for receiving the abortion?<br>क्या आप या आपकी पत्नी ने उन तरीको को इसलिए अपनाया क्योकि आप चाहते थे या यह गर्भपात के लिए निर्धारित शर्त थीा?	Willingly/खुद चाहते थे…...……..……………………..1<br>As condition for getting abortion services/<br>यह एक शर्त थी……………….……..……………2<br>Both willingly and as a condition for getting abortion services/हम चाहते भी थे और इसकी शर्त भी थी………………………………………………….…3<br>Others (specify)/अन्य (स्पष्ट) ………………………….6<br>Don't know/Can't say/पता नहीं/कह नहीं सकते……..8	

Now I would like to ask you some questions regarding any complication that your wife may have experienced in the last abortion.
अब मैं आपसे कुछ प्रश्न पूछना चाहूँगा जो आपकी पत्नी के आखरी गर्भपात के दौरान आई समस्याओं के अनुभव से सम्बधित है

335	Did she have any complication after her last abortion?<br>आखरी गर्भपात के बाद क्या उनको कोई जटिलताए /समस्याए आयी है?	Yes/हाँ……………………………………………..…1<br>No/नहीं……………….………………………………2<br>Don't know/पता नही/कह नही सकते.......................8	 → 401<br>→ 401

336	What kind of abortion related physical complication(s) did she have?<br><br>उनको गर्भपात से सम्बधित किस तरह की शारीरिक जटिलताए /समस्याए आयी?<br><br>**MULTIPLE RESPONSES POSSIBLE.**<br>**एक से अधिक उत्तर संभव है**<br><br>**Probe: Any other problems/ complications?**<br>**पूछें: अन्य कोई जटिलता/समस्या?**	Immediate heavy bleeding……………………..…….…….A<br>तुरंत ज्यादा रक्तस्राव<br>Persistent bleeding (for 2 to 4 weeks post-abortion)……..…B<br>लगातार रक्तस्राव (गर्भपात के पश्चात 2 से 4 सप्ताह तक)<br>Persistent bleeding (for more than 4 weeks post-abortion….C<br>लगातार रक्तस्राव (गर्भपात के पश्चात 4 सप्ताह से ज्यादा)<br>Backache/कमरदर्द………………..………...…………..…D<br>Abdominal pain/cramps/पेट दर्द…………….…….…..…..E<br>Nausea/vomiting/बेहोशी/उल्टी होना………...………………..F<br>Foul smelling discharge/दुर्गंधयुक्त स्राव……………………..…G<br>High-grade fever/तेज बुखार…………………….……..…H<br>Fatigue and weakness/थकावट और कमजोरी…….………..…I<br>Fainting spells/वेहोशी जैसा लगना………...........................J<br>Uterus perforation/rapture/बच्चेदानी मे छेद………..…..…..K<br>Irregular menstruation/अनियमीत महावारी…………….…….L<br>Infertility/sterility/बच्चा न होना………………..……….....M<br>Psychological symptoms: sadness, guilt, sleep,<br>Disturbance/मनोवैज्ञानीक लक्षणः दुःखी, आत्मग्लानी, नीद, झिझक……….…………………………………………N<br><br>Other (specify) ________________________________X<br>अन्य (स्पष्ट)	
338	Did she seek care from a health care provider for any of her complication/s?<br>क्या उन्होने स्वास्थ्यप्रदाता से इन जटिलताए /समस्याओं के लिए कोई सलाह/चिकित्सा ली?	Yes/हाँ……………………………………………..…1<br><br>No/नहीं…………………….……………………………...2	→ **339**
338A	What treatment did she take to overcome from the complication(s)?<br><br>इन जटिलताओ/समस्याओं से उभरने के लिए आपकी पत्नी ने क्या ईलाज लिया?	1 ________________________________<br>2 ________________________________<br>3 ________________________________	**Goto 401**
339	Were she hospitalized because of any of her complications/s?<br>क्या उनको इन जटिलताओ /समस्यों की वजह से अस्पताल में भर्ती होना पड़ा?	Yes/हाँ……………………………………………..…1<br><br>No/नहीं…………………….……………………………2	→ **401**
340	Where were she hospitalized?<br>वह कहाँ भर्ती हुई थी?	District hospital/जिला अस्पताल………………………..1<br>CHC/PHC/SC/समुदायिक चिकित्सा केन्द्र/प्राथमिक चिकित्सा केन्द्र/सब सेन्टर……………………………...2<br>Private Clinic/ Nurshing Home/प्राइवेट क्लिनिक/र्नसिग होम……………………………..……………………3<br>Any other (specify) ___________________________4<br>कोई अन्य (स्पष्ट)	
341	What type of treatment did she receive for this complication?<br>इस जटिलताए /समस्या के लिए उनको किस तरह का इलाज मिला?	IV injections/ Fluids(IV इंजेक्शन/फ्लूइड)………..…A<br>Abortion Again/दुबारा गर्भपात……………………… .B<br>Blood Transfusion/रक्तधान…………………………….C<br>Any other (specify) ___________________________D<br>कोई अन्य (स्पष्ट)	

SECTION 4: KNOWLEDGE AND OPINION ON ABORTION RELATED ISSUES & TECHNIQUES

Now, I would like to ask you few questions about abortion related services that women from your community can access
अब में आपसे कुछ प्रश्न पूछना चाहूँगा जो कि गर्भपात संबंधि सेवाऐं जो आपके समुदाय की महिलाएँ प्राप्त कर सकती है के बारे में हैं।

Q.No	Questions and filters	Coding Categories	Skip To
401	Do you think termination (induced) of pregnancy is legal in India?<br>क्या आपको लगता है कि भारत में गर्भपात वैध है?	Yes, legal/हाँ,कानूनी…………………..…..……..1<br>Yes, legal if woman is married/हाँ, कानूनी अगर महिला शादीशुदा है तो…………………………...2<br>Yes, legal with certain conditions/ हाँ,कानूनी मगर कुछ शर्तो के साथ…………..……3<br>No, illegal/नहीं,गैरकानूनी………………..….….4<br>No idea / Do not know/पता नही……..……...……8	4, 8 → **403**
402	In your opinion, up to what length (month or weeks) of pregnancy abortion is legal in India?<br>आपकी राय में कितने समय (महिने/हफ्ते) तक का गर्भ, गर्भपात के लिए वैध है?<br>**INS: Probe a little & if answer is in month please transfer that into weeks**<br>**निर्देशः पूछें एंव यदि उत्तर महिनों मे हो तो हफ्ते में बदले**	Up to (weeks)……………..…… ☐☐<br>No idea / Do not know……………..…..……..98	
402A	In your opinion, up to what length (month or weeks) of pregnancy abortion can be performed using tablets?<br>आपकी राय में कितने समय (महिने/हफ्ते) तक के गर्भ का गर्भपात गोलियो से हो सकता है?<br>**INS: Probe a little & if answer is in month please transfer that into weeks**<br>**निर्देशः पूछें एंव यदि उत्तर महिनों मे हो तो हफ्ते में बदले**	Up to (weeks)……………..….. ☐☐<br>No idea / Do not know……………..….……..98	

403 & 404

403. In your knowledge, under which of the following conditions or situation abortion is legal in India? आपकी जानकारी में भारत में गर्भपात निम्नलिखित किन हालात या परिस्थितियों में वैध है?

404. Under which of the following conditions or situations do you think a woman should be able to have an abortion? Here, we are interested to know your personal opinion.
निम्नलिखित किन परिस्थितियों/दशाओं में आप सोचते है कि एक स्त्री गर्भपात करवा सकती है? यहाँ मैं आपकी व्यक्तिगत राय जानना चाहता हूँ

[INS: READ ALL OPTIONS A LOUD] **(निर्देशः सभी विकल्प पढ़कर बताएं)**
FIRST ASK Q403 AND THEN ASK Q404 FOR ALL THE OPTIONS **(सभी विकल्पो के लिए पहले प्र० 403 फिर प्र० 404 पुछे।)**

		**Q403: Knowledge on legal status** कानूनी स्थिति की जानकारी			**Q404: Personal opinion should be able to have abortion** गर्भपात के बारे में आपका निजी विचार		
		**Legal** कानूनी	**Illegal** कानूनी नहीं	**No idea** पता नहीं	**Yes** हाँ	**No** नहीं	**No idea** पता नहीं
A	If the woman is unmarried & pregnant<br>यदि महिला अविवाहित और गर्भवती हो	1	2	8	1	2	8
B	If the pregnancy is an accident (result of a contraceptive failure)/ यदि अप्रत्याशित गर्भ हो (गर्भ निरोधक के निष्क्रिय हो जाने की वजह से)	1	2	8	1	2	8

		**Legal** कानूनी	**Illegal** कानूनी नहीं	**No idea** पता नहीं	**Yes** हाँ	**No** नहीं	**No idea** पता नहीं
C	If the pregnancy is a result of rape/ यदि गर्भ बलात्कार की वजह से हो	1	2	8	1	2	8
D	If the woman's health is endangered by the pregnancy/ यदि गर्भ से महिला का स्वास्थ्य खतरे में हो	1	2	8	1	2	8
E	If there is a strong chance of serious defect in the baby/ यदि पैदा होने वाले बच्चे में काफी गंभीर त्रुटि होने की संभावना हो	1	2	8	1	2	8
F	If the woman is more than 20 weeks pregnant यदि गर्भ 20 हफ्ते से ज्यादा का हो	1	2	8	1	2	8
G	If the fetus is female/ यदि भ्रूण लड़की हो	1	2	8	1	2	8
H	If the fetus is male / यदि भ्रूण लड़का हो	1	2	8	1	2	8
I	If the woman does not want another child यदि महिला एक और बच्चा ना चाहती हो	1	2	8	1	2	8
J	If the woman cannot afford the child यदि महिला बच्चे का खर्च वहन करने मै असमर्थ हो	1	2	8	1	2	8

405	Can you tell me where do married women from your community normally go to access abortion related services?<br>क्या आप बता सकते है कि आपके समुदाय की विवाहित महिलाएं आमतौर पर गर्भपात सम्बन्धित सेवाओं को पाने के लिए कहाँ जाती है?<br><br>**MULTIPLE RESPONSE POSSIBLE**<br>**(एक से अधिक उत्तर संभव हैं)**<br><br>**WRITE DOWN THE NAME OF THE DOCTOR IN CASE YOU ARE NOT SURE ABOUT FACILITY TYPE**<br><br>**अगर सुविधा के प्रकार के बारे में नही बता सकते तो डॉक्टर का नाम लिखे।**	Govt/District hospital Khunti/ सरकारी अस्पताल/जिला अस्पताल खुटी…….…..…A<br>Govt/Rural hospital Silli/सरकारी/ ग्रामीण अस्पताल सिल्ली ……..…………………….....B<br>PHC/RH/CHC/ समुदायिक चिकित्सा केन्द/ ग्राम अस्पताल/प्राथमिक चिकित्सा केन्द्र…..........C<br>ANM/Nurse/ए एन एम/नर्स…......…..…..……D<br>Doctor-Private Clinic (Ipas provider)................ E<br>डाक्टर–प्राइवेट क्लिनिक (IPAS सेवाप्रदाता)<br>Doctor- Other Private Clinic .... F<br>डाक्टर–अन्य प्राइवेट क्लिनिक<br><br>Nursing Home/नर्सिंग होम….…………....…..…G<br>Chemist Shop/दवा की दुकान……….……....…..H<br>Ayurved Doctor/आयुवेदिक डाक्टर…….…..…..I<br><br>Dai/TBA/प्रशिक्षित/दाई………………...……...J<br>Other traditional Healer (Ojha)/ ओझा……........K<br>Village practitioner (unqualified)/……………....L<br>ग्राम सेवाप्रदाता (अप्रशिक्षित)<br>Other (specify) ________________________.X<br>अन्य (स्पष्ट)<br>No idea/Do not know/पता नही………....…...…Z	

CHECK BOX NO. 6
CHECK Q209, Q211 & Q214
Wife had an induced abortion during last 3 years? Yes=1 → Q411 No=2 → Q406
पत्नी का गर्भपात पिछले 3 साल में हुआ है ?

406	If your wife or someone from your friend/relative needed an abortion, whom would you be willing to go to?<br>यदि आपकी पत्नी या आपके दोस्तों/रिश्तेदारों में किसी को गर्भपात की जरूरत हो तो आप किसके पास जाना चाहेंगे?<br><br>**MULTIPLE RESPONSE POSSIBLE**<br>**(एक से अधिक उत्तर संभव हैं)**<br><br>**WRITE DOWN THE NAME OF THE DOCTOR IN CASE YOU ARE NOT SURE ABOUT FACILITY TYPE**<br><br>**अगर सुविधा के प्रकार के बारे में नही बता सकते तो डॉक्टर का नाम लिखे।**<br><br>**(INS: Please keep a copy of list of Ipas Supported providers with you while interviewing)**	Govt/District hospital Khunti/ सरकारी अस्पताल जिला अस्पताल खुटीं……………..……A<br>Govt/Rural hospital Silli/सरकारी/ ग्रामीण अस्पताल सिल्ली ………..…………….………………….…….B<br>PHC/RH/CHC/ समुदायिक चिकित्सा केन्द्र/ ग्राम अस्पताल/प्राथमिक चिकित्सा केन्द्र..……………......C<br>ANM/Nurse/ए एन एम/नर्स…..………….........….D<br>Doctor-Private Clinic (Ipas provider)...................... .E<br>डाक्टर–प्राइवेट क्लिनिक<br>Doctor- Other Private Clinic .... F<br>डाक्टर–अन्य प्राइवेट क्लिनिक<br><br>Nursing Home/नर्सिंग होम…..........……….…….G<br>Chemist Shop/दवा की दुकान………….……….H<br>Ayurved Doctor/आयुर्वेदिक डाक्टर……….…….…..I<br>Dai/TBA/प्रशिक्षित/दाई……………….........…...J<br>Other traditional Healer (Ojha)/ ओझा……………......K<br>Village practitioner (unqualified)/………..……...L<br>ग्राम सेवाप्रदाता (अप्रशिक्षित)<br>Other (specify)…………………………….....…..X<br>अन्य (स्पष्ट)<br>No idea/Do not know/पता नही…………..………Z	
407	How did you come to know that you can go to the provider for abortion?<br><br>आपको कैसे पता चला कि सेवाप्रदाता के पास गर्भपात के लिए जा सकते हैं?	ANM suggested/ए एन एम ने सलाह दी ……...…...1<br>ASHA/SAHIYA sugested /सहिया दीदी/आशा ने सलाह दी …………………….………...…..2<br>Anganwadi Worker (AWW) suggested/ आंगनवाड़ी कार्यकर्ता ने सलाह दी……..………………...….3<br>Trained Birth Attendent (TBA) suggested/ प्रशिक्षित दाई ने सलाह दी ………………………………….4<br>Any other health worker suggested/ अन्य स्वास्थयकर्ता ने सलाह दी ……………………………..………5<br>NGO Worker/ NGOकार्यकर्ता ……………….……….6<br>Wife shared/ पत्नी ने बताया ……………….…….7<br>Family members/Friends shared/ परिवार के सदस्य/मित्र ने बताया…………………...……...8<br>Wall Sign/दिवार लेखन द्वारा…………………..…..9<br>Street Drama/नुक्कड़ नाटक…………...…………….10<br>Other (Specify)/अन्य (स्पष्ट करें)………………...….11	

408	In your opinion, who can provide abortion related services? Would you say any health worker, or any qualified doctor, or a qualified doctor who is trained and certified or you have no idea? आपकी राय मे गर्भपात संबधित सेवायें कौन दे सकता है? क्या आप कहेंगे कोई योग्य डॉक्टर /योग्य प्रशिक्षित एवं प्रमाणित डॉक्टर /महिला स्पेशलिस्ट / या पता नहीं	Any health worker/कोई भी स्वास्थ्य कार्यकर्ता…...…...1<br>Any qualified doctor/ कोई भी योग्य डाक्टर…..…….2<br>Trained & Certified doctor/प्रशिक्षित और प्रमाणित डाक्टर……………………….……………...…3<br>Women's Specialist doctor (Ob-gyn) स्त्री रोग विशेषज्ञ (Ob-gyn) ………………….……..………..……4<br>No idea / Do not know / पता नही ……...…………8	
409	In your knowledge, what is the nearest location where you or your neighbor can access abortion related services? आपकी जानकारी में सबसे नजदीकी जगह कौन सी है जहां आप या आपके पड़ोसी गर्भपात सम्बन्धी सेवायें प्राप्त कर सकते है?	Govt/District hospital Khunti/सरकारी अस्पताल……..1<br>Govt/Rural hospital Silli/सरकारी / ग्रामीण अस्पताल सिल्ली ……..…………………….……………..…...2<br>PHC/RH/CHC/ सामुदायिक चिकित्सा केन्द्र / ग्राम अस्पताल / प्राथमिक चिकित्सा केन्द्र…………………....3<br>Other Place (specify)________________________6<br>अन्य स्थान (स्पष्ट)<br>No idea / Do not know/पता नही …………..……..….8	

411. Now, I would like to talk about different methods by which abortion can be performed – the various ways in which a couple can abort an unwanted pregnancy. Could you tell me what methods have you heard of? **अब मै गर्भपात के अलग अलग तरीकों के बारे में बात करना चाहूँगा जिनसे गर्भपात किया जा सकता है – वे विभिन्न तरीके जिनके द्वारा एक दम्पति अपने अनचाहे गर्भ को गिरा सकते है। क्या आप बता सकते है कि आपने गर्भपात के किन तरीकों के बारे में सुना है।**

INS: DO NOT READ THE LIST OR PROMPT. RECORD ANSWERS GIVEN SPONTANEOUSLY FIRST BY CODING '1'.
कृप्या लिस्ट में लिखे गयें उतरों को पढ़कर न सुनायें। उत्तरदाता को उत्तर स्वंय देने दें। स्वयं दिये गये उतरों को "1" कोड करें।
FOR METHODS NOT MENTIONED SPONTANEOUSLY ASK: (जिन विधियों के नाम उत्तरदाता न ले सके पूछियें)

Now I am going to read you a list of methods of abortion. For each method I mention, please tell me if you have ever heard of the method. अब मैं गर्भपात के तरीकों के बारे में पड़ने जा रहा हूँ। कृप्या प्रत्येक तरीके के बारे में बताये कि क्या आपने उसके बारे में सुना है।
INS: RECORD THE ANSWER ACCORDINGLY UNDER CODE 2 "Yes on probing" or Code 3 "No, on probing"
INS: FOR EACH METHOD THAT THE RESPONDENT HAS HEARD OF (either prompted or unprompted), ASK THE APPROPRIATE FOLLOW-UP QUESTIONS BELOW. IF THE RESPONDENT HAS NEVER HEARD OF A GIVEN METHOD, DO NOT ASK THE FOLLOW-UP QUESTIONS FOR THAT METHOD.
बताने पर दिये गये उत्तर को "2" कोड दे और बताने पर नहीं दिये गए उत्तर को "3" कोड दे।
INST.- जिन विधियों के बारे में उत्तरदाता ने सुना हो (स्वयं बताये गये या आपके बताये गए) उन विधियों के बारे में आगे के प्रश्न पुछे। यदि उत्तरदाता विधि के बारे में नहीं जानते, उनसे उस विधि के बारे में आगे के प्रश्न ना पुछें।

411	**Questions and filters**<br>*What methods of abortion have you heard of?*<br>आपने गर्भपात के किन–किन तरीकों के बारे मे सुना है?	What methods of abortion have you heard of? आपने गर्भपात के किन–किन तरीकों के बारे मे सुना है?	Specific knowledge about the method<br>तरीके के बारे में विस्तृत जानकारी	Where do you think it is available<br>आप क्या सोचते है कि यह कहाँ उपलब्ध है
		X1	X2	X3
A	<u>MVA</u><br>Abortion can be performed by a hand held machine that pulls out the fetus through suction<br>गर्भपात हाथ से इस्तेमाल करने वाली मशीन से हो सकता है जो कि भ्रूण को खिचाव के द्वारा बाहर निकालता है	1 Yes, unprompted हाँ, स्वयं बताने पर<br>2 Yes, on probing हाँ, बताये जाने पर<br>3 No, on probing नहीं, बताये जाने पर<br>**Next method**	A surgical method normally done under local anesthesia<br>आमतौर पर, बेहोश करके सर्जीकल तरीके से गर्भपात<br>1 Yes हाँ<br>7 Other response अन्य कोई<br>8 Do not know जानते नहीं	1. District hospital<br>2. PHC<br>3. CHC<br>4 Private doctor /Nursing home<br>5. ANM<br>6. Chemist<br>7. Other_______________<br>8. Dk/CS<br>**PLEASE NOTE DOWN THE LOCATION OF FACILITY**<br>**कृप्या सेवा मिलने वाले स्थान का नाम लिखें**

B	<u>D&C</u><br><br>A doctor opens the cervix and scrapes the uterus to remove the fetus<br>डॉ गर्भाशय नली को खोलता है और भ्रूण को निकालने के लिए गर्भाशय को खरोचता है	1 Yes, unprompted हाँ, स्वयं बताने पर<br>2 Yes, on probing हाँ, बताये जाने पर<br>3 No, on probing नहीं, बताये जाने पर ↓<br><br>**Next method**	A surgical method normally done under general anesthesia<br>आमतौर पर, बेहोश करके सर्जीकल तरीके से गर्भपात<br>1 Yes हाँ<br>7 Other response अन्य कोई<br>8 Do not know जानते नहीं	1. District hospital<br>2. PHC<br>3. CHC<br>4 Private doctor /Nursing home<br>5. ANM<br>6. Chemist<br>7. Other_______________<br>8. Dk/CS<br><br>**PLEASE NOTE DOWN THE LOCATION OF FACILITY**<br>**कृप्या सेवा मिलने वाले स्थान का नाम लिखें**
C	<u>Medical Abortion</u><br><u>(Abortion with pills or tablets)</u><br>Abortion can be done by taking two different types of tablets. The second dose should be taken after 3 days of the first dose.<br>(गोलियों द्वारा गर्भपात)<br>दो अलग तरह की गोलीया खाकर गर्भपात कराया जा सकता है। दुसरी गोली, पहली गोली के तीन दिन बाद लेनी चाहिए।	1 Yes, unprompted हाँ, स्वयं बताने पर<br>2 Yes, on probing हाँ, बताये जाने पर<br>3 No, on probing नहीं, बताये जाने पर ↓<br><br>**Next method**	A woman needs to consume tablets/ pills for complete abortion<br>पूर्ण गर्भपात के लिए महिला को गोली खाने की जरूरत होती है<br>1 Yes हाँ<br>7 Other response अन्य कोई<br>8 Do not know जानते नहीं	1. District hospital<br>2. PHC<br>3. CHC<br>4 Private doctor /Nursing home<br>5. ANM<br>6. Chemist<br>7. Other_______________<br>8. DK/CS<br><br>**PLEASE NOTE DOWN THE LOCATION OF FACILITY**<br>**कृप्या सेवा मिलने वाले स्थान का नाम लिखें**
D	<u>Any other method</u><br><br><u>Please specify (it might be any method)</u><br>कोई अन्य तरीका–<br>यदि कोई अन्य तरीका हो, तो विवरण दें			**PLEASE NOTE DOWN THE LOCATION OF FACILITY**<br>कृप्या सेवा मिलने वाले स्थान का नाम लिखें

CHECK BOX NO. 7

CHECK Q411C: Mentioned Medical Abortion =1 → Q412 Not Mentioned Abortion =2 → Section 5
यदि **Q411C** मे औषधिय गर्भपात(**Medical Abortion**) है तो→ **Q412** यदि औषधिय गर्भपात नहीं है तो → **Section 5**

412	You just have mentioned about pills and tablets. How did you come to know about this method?<br><br>**[WRITE DOWN IN VERBATIM]**<br><br>आप अभी टेबलेट/गोली के संबंध में बताया। आपको इस विधि/तरीके के संबंध में कैसे जानकारी मिली?<br>**बतांए गए उत्तर को लिखे**	ANM/ए एन एम…..……………………………...………….1<br>AWW/ ए डब्लू डब्लू……………………………...………….2<br>TBA/प्रशिक्षित दाई….………………………………...…..3<br>IPC/ Group Meeting/आई पी सी/समूह की बैठक……….4<br>Wall Sign/ दिवार लेखन द्वारा ……………………………...5<br>Street Drama/ नुक्कड नाटक………………….……………..6<br>Other (specify)________________________________9<br>अन्य (स्पष्ट)	
413	Up to what length of pregnancy a woman can terminate her pregnancy by taking tablets?<br><br>RECORD IN VERBATIM<br><br>गर्भ के कितने समय बाद तक गोली/टेबलेट से गर्भपात किया जा सकता है?<br><br>**INS: Please transfer the responses into weeks**<br>**निर्देशः कृपया उत्तर को हफ्ते में बदले एवं लिखे ।**	Record Actual Response ☐☐<br>बताये गये उत्तर को लिखें।<br>____________________<br><br>Don't know/पता नही ……………………….……………8	

414	Would you please tell me how many tablets does a woman need to complete the abortion?<br>क्या आप बता सकते है कि गर्भपात के लिये गर्भवती महिला को कितनी गोलिया लेनी होंगी?	कितनी गोलियाँ<br>Number of tablets……………………. ☐<br><br>Don't know/ No idea/पता नही …………………..……….8	
415	Do you know whether a woman needs to take all tablets at once or in sequence to complete the abortion? क्या आप को मालूम है कि गर्भपात के लिये महिला को एक साथ सारी गोलिया खानी होती है या गोलियों को क्रम से लेना होता है?	All at once/सभी एक साथ……………………….…….……1<br>In sequence/क्रम से…………………….….……..…………...2<br>No idea/Can't say/पता नही / कह नही सकते……......……3	→ 501<br><br>→ 501
416	If in sequence, would you be able to tell me the sequence?<br>यदि क्रम में लेना होता है, तो क्या आप बता सकते है कि इसका क्रम क्या होता है?<br>**RECORD IN VERBATIM. PROBE FOR THE DAY OF CONSUMPTION.**<br>**जानने कि कोशिश किजिए कि गोलियाँ कब ली गई थी।**	(बताये गए उत्तर को लिखें)<br>Record Actual Response ________________________<br>____<br><br>Don't Know /Can't say/पता नही / कह नहीं सकते……….8	

SECTION 5: COMMUNICATION & COMMUNITY EXPOSURE

Q.No	Questions and filters	Coding Categories	Skip To
501	In the last one year, have you received any information on family planning?<br>पिछले एक साल में क्या आपको परिवार नियोजन पर कोई जानकारी मिली है?<br>If yes where /from whom did you get this information from?<br>यदि हाँ, तो कहाँ से / किससे आपको यह जानकारी मिली थी?<br><br>**(Multiple responses possible)**<br>**(एक से ज्यादा उत्तर संभव है)**	TV/टीवी………………………..…….……..……..….A<br>Radio/रेडियो….……………………….……..…..……….....B<br>Newspaper/अखवार….…………………………...…...…C<br>Wall Sign/दीवार चिन्ह…….………………...……….D<br>Poster/billboard/पोस्टर / विज्ञापन……...........…...…...E<br>Community Club/सामूदायिक क्लब…….…………...…..F<br>NGO worker (IPC didi)/ NGO कार्यकर्ता………..…. .G<br>Village doctor (local practitioner)/ग्राम डाक्टर (स्थानीय डाक्टर)…..……….H<br>Chemist/केमिस्ट..I<br>ANM/Nurse/ए एन एम / नर्स……...…..........……….J<br>Sahiya/ASHA/AWW/सहीया / आशा / ए डब्लू डब्लू…......................………........……..………….K<br>Dai/TBA/प्रशिक्षित दाई……..………...…...……….L<br>Government health facility/सरकारी स्वास्थ्य सुविधा…………………….………..………..…....M<br>Private provider/प्राइवेट कार्यकर्ता…..…..........……..N<br>Wife/ पत्नी ……………………..........……….O<br>Family member/ Friends/neighbors/परिवार के सदस्य / दोस्त / पडोसी……………….……………...……....P<br><br>Other (specify)______________________________Q<br>अन्य (स्पष्ट)<br>No information/पता नही……………….…......…...R	 <br><br><br><br><br><br><br><br><br><br><br><br><br><br><br><br><br><br><br>→ 503

502	Do you remember what information you received?<br>क्या आपको याद है कि आपको क्या जानकारी मिली थी?	________________	
503	In the last two years, do you remember seeing or hearing or attending any event promoting message or information on abortion related issues?<br>कृपया याद करके बताऐं कि पिछले दो वर्ष मे क्या आपने कोई ऐसे कार्यक्रम/मीटिंग में भाग लिया/देखा/सुना जिसमें गर्भपात संबंधित विषय पर जानकारी दी गई है।<br><br>**IF "NO" OR "DON'T KNOW", PROBE A BIT:**<br><br>**Do you recall anywhere you saw/hear any message on abortion?**<br>**यदि "नहीं" या "पता नही", तो पूछेः–**<br>**क्या आपने कहीं भी गर्भपात पर कोई संदेश सुना/देखा है?**	Yes/हाँ……………..…………………………….…1<br><br>No/नहीं…………………………….……………....2<br><br>Yes, with little probing/हाँ/कुछ बताने पर………….3	

SECTION 6: BEHAVIORAL ATTRIBUTES [COMMUNICATION CUES]

In the next couple of questions, I am interested to know your opinion on certain issues related to abortion practices in your community. I will read out some statements. Please let me know whether you 'strongly agree', 'agree somewhat' or 'neither agree or nor disagree' 'disagree somewhat' or "strongly disagree'. **आगे के कुछ प्रश्नों में मैं आपके समुदाय/बिरादरी में गर्भपात की रीतियों के संबध में आपकी राय जानना चाहूँगा। मैं आपको कुछ कथन पढकर सुनाउँगा। कृपया आप मुझे बताये कि आप इनसे "पूर्ण सहमत", "कुछ हद तक सहमत", या "न सहमत न असहमत", कुछ हद तक असहमत या पूर्ण असहमत है।**

INS: FEW SCALE STATEMENTS ARE IN REVERSE ORDER, PLEASE READ AND CODE CAREFULLY.

		**Strongly disagree** पुर्ण असहमत	**Disagree somewhat** कुछ हद तक असहमत	**Neither agree nor disagree** न सहमत ना असहमत	**Agree somewhat** कुछ हद तक सहमत	**Strongly Agree** पुर्ण सहमत
	**Availability/ उपलब्धता**					
A	We do not have any option to get abortion services at a facility close to our village.<br>गर्भपात कराने के लिऐ हमारे गाँव के आस पास/हमारे गाँव के करीब में सुविघा उप्लब्ध नही हैं	1	2	3	4	5
B	Abortion services are normally available at urban based private clinic/nursing home.<br>गर्भपात की सुविधा आमतौर पर शहरों में प्राइवेट क्लिनिक/नर्सिग होम में उपलब्ध हैं	1	2	3	4	5
C	We don't have any information on abortion providers / हम लोगों को गर्भपात के तरीकों एंव गर्भपात करने वाले सेवाप्रदाताओं की जानकारी नहीं हैं	1	2	3	4	5

		Strongly disagree पुर्ण असहमत	Disagree somewhat कुछ हद तक असहमत	Neither agree nor disagree न सहमत ना असहमत	Agree somewhat कुछ हद तक सहमत	Strongly Agree पुर्ण सहमत
	**Affordability/ व्यय क्षमता**					
D	Abortions usually cost more than any other treatment गर्भपात में दूसरे इलाज की अपेक्षा अधिक खर्च पडता हैं	1	2	3	4	5
E	Doctors usually charge more if they use any surgical procedure / यदि चिकित्सक शल्य चिकित्सा का प्रयोग करता है तो वह अधिक पैसे मांगता है	1	2	3	4	5
	**Social Norms/ सामाजिक रीति**					
F	In this community, abortions/pregnancy among **unmarried** women are usually being treated as bad/sin./ इस समुदाय में, अविवाहित महिलाओं के गर्भ/गर्भपात को बुरा/पाप माना जाता हैं	1	2	3	4	5
G	In my community, it is not acceptable to talk about any abortion related issue. हमारे समुदाय/बिरादरी में गर्भपात से जुडें किसी भी विषय पर बात करना बुरा माना जाता हैं	1	2	3	4	5
H	In this community, people who want to terminate a pregnancy prefer to go to a known health worker (like Dai, ANM didi) rather than a medical doctor हमारे समुदाय/बिरादरी में जो गर्भपात कराना चाहते है वह चिकित्सक की अपेक्षा उस स्वास्थ्यकर्मी के पास जाना पसंद करते है जिन्हें वह जानते है (जैसे दाई, एएनएम दीदी)	1	2	3	4	5
I	Couple who want to terminate their unwanted pregnancy would like to approach a provider who respects confidentiality./ अनचाहे गर्भ का गर्भपात कराने के लिये दंपति उस सेवाप्रदाता के पास जाना पसंद करेगें जहॉ उन्हे गोपनीयता का भरोसा हो।	1	2	3	4	5
	**Social Support/ सामाजिक सहायता**					
K	I can discuss all use of contraceptives with my spouse. मैं गर्भनिरोधक के सभी उपयोग के बारे में अपनी पत्नी से बात कर सकता हूं।	1	2	3	4	5
L	If we were to go for an abortion, my wife would support me / यदि मैं गर्भपात कराने का निर्णय लेता हूं तो मेरी पत्नी इसमे मेरा साथ देगी।	1	2	3	4	5
M	If we (me & my wife) were to go for an abortion, my other family members (say parents) would support me. यदि, हम (मै और मेरी पत्नी) गर्भपात के लिए जाते है तो मेरे अन्य परिवार जन (जैसे माता पिता) मेरा साथ देंगें।	1	2	3	4	5
N	If we need to go for abortion, I would have to do so without telling anyone/ यदि हमें (मै और मेरी पत्नी) को गर्भपात कराने जाने की जरूरत पड़े तो, हमें यह बिना किसी को बताऐ कराना होगा।	1	2	3	4	5
O	If we were to go for abortion, my friends and relatives would make fun of me./ यदि हमे गर्भपात के लिए जाना पड़े तो मेरे मित्र एंव संबंधी हमारा मज़ाक बनाऐंगे।	1	2	3	4	5

		Strongly disagree पुर्ण असहमत	Disagree somewhat कुछ हद तक असहमत	Neither agree nor disagree न सहमत ना असहमत	Agree somewhat कुछ हद तक सहमत	Strongly Agree पुर्ण सहमत
P	If we were to go for abortion, doctors and health care providers would treat me rudely/ यदि हमें गर्भपात के लिए डॉ या स्वास्थ्य कार्यकर्ता के पास जाना पड़े, तो उनका व्यवहार अच्छा नहीं होगा	1	2	3	4	5
	**Self-Efficacy/ स्वंय क्षमता**					
Q						
R						
S						
T	I can confidently talk to a medical doctor about issues related to abortion / मैं विश्वास के साथ एक चिकित्सक से गर्भपात संबंधी विषयों पर बातचीत कर सकता हूँ।	1	2	3	4	5
U	I can initiate discussing issues related to abortion with my neighbors/मैं अपने पडोसियों के साथ गर्भपात से जुडे विषयों पर बात करने की पहल कर सकता हूँ।	1	2	3	4	5
V	I can help my friends and relatives to choose a trained doctor./मै अपने मित्रों सगे संबधियों को प्रशिक्षित चिकित्सक चुनने में सहायता कर सकता हूँ।	1	2	3	4	5
	**Issue Severity/ विषय की गम्भीरता**					
W	Women may suffer with severe complications if abortions are carried out by untrained providers / गर्भपात अप्रशिक्षित चिकित्सक के द्वारा कराने से महिला को जटिल समस्याओं का सामना करना पड़ सकता है।	1	2	3	4	5
X	There is a risk to a mother's health if abortion is done at a mature stage / यदि गर्भपात 12 सप्ताह के बाद कराया जाए तो महिला के स्वास्थ्य को खतरा हो सकता हैं।	1	2	3	4	5
Y	Repeated abortions harm woman's health/लगातार गर्भपात महिला के स्वास्थ्य को नुकसान पहुँच सकता हैं।	1	2	3	4	5

CHECK BOX NO. 8
CHECK Q209, Q211 & Q214
Reported an induced abortion of wife during last 3 years? Yes =1 → Q701 No=2 → Continue
पिछले 3 वर्ष में पत्नी का गर्भपात कराया हैं।

	Attitude					
Z	Abortion is immoral / गर्भपात अनैतिक है।	1	2	3	4	5
AA	It is better to deliver the child than to abort it / गर्भपात कराने की अपेक्षा बच्चे को जन्म देना बेहतर हैं	1	2	3	4	5
AB	I feel guilt when I think for abortion / मुझे पाप / अपराध का आभास होता है जब मैं गर्भपात कराने की सोचती हूँ।	1	2	3	4	5
AC	Abortion is very private issue. It is better not to disclose this issue with outsiders/गर्भपात निजी मामला है। इस मामले को दूसरो को बताना ठीक नहीं है।	1	2	3	4	5

CMS social

		Strongly disagree पुर्ण असहमत	Disagree somewhat कुछ हद तक असहमत	Neither agree nor disagree न सहमत ना असहमत	Agree somewhat कुछ हद तक सहमत	Strongly Agree पुर्ण सहमत
AD	Occasional abortion is better than regular contraception / निरंतर गर्भनिरोध से कभी कभार गर्भपात करा लेना अच्छा हैं।	1	2	3	4	5
	**Beliefs** मान्यताएं / विश्वास					
AE	Village practitioners are more reliable than outside doctors in terms of confidentiality./जहां तक गोपनीयता का प्रश्न है गांव के डाक्टर बाहरी डाक्टरों से बेहतर हैं।	1	2	3	4	5
AF	A woman will never be pregnant in future if she goes for abortion एक महिला कभी भी भविष्य में गर्भवती नहीं हो सकती है यदि वह गर्भपात करवाती है।	1	2	3	4	5
AG	Women can not work normally immediately after abortion / गर्भपात के बाद महिला तुंरत सामान्य रुप से काम–काज नहीं कर सकती।	1	2	3	4	5
AH	A woman can die of abortion/ महिला को गर्भपात से मृत्यु हो सकती है।	1	2	3	4	5
AI	I know woman from my community who was seriously ill from abortion related complications/ मै अपनी समुदाय में ऐसी महिला को जानता हूँ जिसकी गर्भपात कराने से हालात बहुत खराब हो गई थी।	1	2	3	4	5

SECTION 7: CLOSING STATEMENT

We have reached the end of the interview. I realize that it was quite long and want to thank you again for participating. The information you have given me will be very helpful in improving services for women. As I know some of the matters we discussed today may have been embarrassing or made you feel uncomfortable, please call Mr. Debashis Sinha on 09334196224 Ipas Ranchi Office if you would like to talk to someone about this or any other problem that may arise due to this survey. हम साक्षात्कार समाप्त कर चुके। मैं आपका साक्षात्कार में भाग लेने के लिए धन्यवाद देता हूँ। आपके द्वारा दी गयी जानकारी महिलायों को पहुँचाई जाने वाली सेवाओं में सुधार लाने के लिए बहुत सहायक सिद्ध होगीं। साक्षात्कार में जिन मुद्दों / विषयो पर बात हुई उनमें से कुछ विषयो ऐसे हो सकते है जिसपर आपको बात करने मे हिचकिचाहट हुई होगी या आपको इस पर बात करने में संकोच हुआ होगा। इस सर्वेक्षण के बाद आपको कोई जानकारी लेनी हो या इस सर्वेक्षण के कारण आप को किसी असुविधा का सामना करना पडा हो तो आप कृप्या **श्री देवाशिश सिन्हा से फोन न0 09334196224** आइपास, राँची आफिस मे सम्पर्क करें।

Q.No	Questions and filters	Coding Categories	Skip To
701	Before I depart, do you have any question about the survey or the study? मेरे जाने के पहले, क्या आपके इस अध्ययन / सर्वे के बारे में कुछ प्रश्न है?	Yes/हाँ……………….………………..……1<br>No/नहीं……..……………………..………….2	
	**यदि हाँ, तो क्या**	____________<br>____________	

THANK AND TERMINATE THE INTERVIEW

धन्यवाद, साक्षात्कार समाप्त हुआ
