# Supplementary material for: Evaluation of a multi-pronged intervention to improve access to safe abortion care in two districts in Jharkhand
Source: BMC Health Serv Res. 2014 May 21;14:227. doi: 10.1186/1472-6963-14-227 (PMC4035795; doi:10.1186/1472-6963-14-227)
Supplement: Additional file 2 — Abortion related knowledge and care seeking behavior and practice in Jharkhand: A KABP follow-up study among women and men of reproductive age. Women’s questionnaire. [file 1472-6963-14-227-S2.pdf]

CMS social

HOUSEHOLD LEVEL SURVEY

Abortion related knowledge and care seeking behavior and practice in Jharkhand: A KABP follow-up study among women and men of reproductive age

WOMEN'S QUESTIONNAIRE

PSU IDENTIFICATION	CODES
1. District: ______________________	[ ]
2. Block : ______________________	[ ][ ][ ]
3. Panchayat: ______________________	[ ][ ][ ]
4 Village: ______________________	[ ][ ][ ]
**CLIENT IDENTIFICATION:**	
5. Respondent's ID No (Record from listing sheet): ______________	[ ][ ][ ]
6. Address (with landmark): ______________________	
**INTERVIEW STATUS:**	
7. Date of interview<br>**Date** **Month** **Year**<br>[ ][ ] [ ][ ] [ ][ ][ ][ ]	
8. Interview Status: ______________________<br>1 Completed<br>2 Partly Completed<br>3 Refused by respondent<br>4 Refused by husband/ attendants<br>5 Not at Home<br>6 Other (specify)______________________	[ ]

Name & Code	**Investigator**	**Monitored by**	**Edited by**
	______________<br>[ ][ ]	______________<br>[ ][ ]	______________<br>[ ][ ]

INTRODUCTION AND CONSENT

नमस्ते मेरा नाम ———————————————— है। मैं सेन्टर फार मीडिया स्टडीज की तरफ से हूँ। जो कि एक गैर सरकारी शोध संस्था है।

मेरे साथ बातचीत तथा समय देने का आपका घन्यवाद। यह सर्वे सेन्टर फार मीडिया स्टडीज और आइपास, के द्वारा करवाया जा रहा है। आइपास दिल्ली में स्थित अन्तर्राष्ट्रीय संस्था है जो महिलाओं के प्रजनन स्वास्थ्य और अधिकारों खासकर सुरक्षित गर्भपात की सेवाओं को प्राप्त करने में उनकी मदद करती है। इस अध्ययन के द्वारा हम महिलाओं के स्वास्थ्य, खासकर गर्भ के बारे में और महिलाएँ गर्भ को रोकने के लिए क्या कर सकती है और ऐसी परिस्थिति में जब वह गर्भ ना चाहते हुए भी गर्भ धारण कर ले के बारे में भी जानना चाहते है। और स्वास्थ्य सेवाऐं जो इस तरह की परिस्थितियों में महिलाओं के लिए उपलब्ध है। यह अध्ययन झारखण्ड के दो जिले में हो रहा है और हम आप जैसी महिलाओं से इन मुद्दों के बारे में बात कर रहे है। यह जानकारी महिलाओं की इस तरह की जरुरतों के लिए कार्यक्रम को बेहतर बनाने और जवाबों को समझने में हमारी मदद करेगी।

इस अध्ययन मे भाग लेने के लिए मै आपकी अनुमति लेना चाहूँगी। खासतौर से मै आप जैसी औरतों के गर्भ से सम्बधित उनके अनुभव के बारे में आपसे प्रश्न पूछना चाहूँगी। इस सर्वे में भाग लेना स्वैच्छिक है। यदि आप सर्वे में भाग नही लेना चाहती है या साक्षात्कार के दौरान किसी भी समय साक्षात्कार बन्द कर देती है, तो किसी भी क्लीनिक व अन्य जगहों से आपको मिलने वाली सेवाओं पर वर्तमान या भविष्य में कोई असर नही पड़ेगा।

हम आपको भरोसा दिलाते है कि जो भी जानकारी आप हमें देंगीं उन्हे पूर्ण रूप से गुप्त रखा जायेगा और इस जानकारी को सिर्फ महिलाओं की जरूरतो को समझने और उनसे सम्बन्धित कार्यक्रमों के विकास के लिए इस्तेमाल किया जायेगा। जब इस अध्ययन के नतीजे प्रकाशित होंगे, आपकी गोपनियता को पूर्ण रूप से सुरक्षित रखा जायेगा। हमे उम्मीद है कि आप हमारे प्रश्नो का जबाव देने के लिए तैयार होंगी, आपकी राय और अनुभव हमारे लिए महत्वपूर्ण और लाभदायक होंगे।

यदि आप साक्षात्कार के लिए तैयार हैं, तो हम ऐसी जगह बातचीत करेंगे जहां कोई हमारी बातचीत ना सुन सके, इस बातचीत मे लगभग आधा घंटे का समय लगेगा।

यदि आपका इस अध्ययन के बारे में कोई प्रश्न है तो श्री देवाशिश सिन्हा - फोन न0 09334196224, से सम्पर्क कर सकती है।

उत्तरदाता का हस्ताक्षर ....**दिनांक** ....

मैने इसकी पुष्टि कर ली है कि उत्तरदाता साक्षात्कार के लिए सहमत/असहमत है। (जो लागू नही उसे काट दें)

साक्षात्कारकर्त्ता के हस्ताक्षर..**दिनांक** ....

उत्तरदाता साक्षात्कार के लिए सहमत ....1 ↓ उत्तरदाता साक्षात्कार के लिए असहमत........ 2 समाप्त ⟶

क्या मै अब साक्षात्कार शुरू कर सकती हूँ?

Thank you for agreeing to take part in this study. I want to start by asking you a few general questions about you and your household. इस सर्वे में भाग लेने हेतू सहमति के लिए धन्यवाद। अब मैं आपके एवं आपके परिवार संबंधित कुछ सामान्य प्रश्न पूछना चाँहूगी।

SECTION 1: RESPONDENT'S BACKGROUND & HOUSEHOLD INFORMATION

Q.No	Questions and filters	Coding Categories	Skip to
100	How old were you at your last birthday?<br>आपने अपने आखिरी जन्मदिन पर कितने वर्ष पूरे किए?<br>[COLLECT AGE IN COMPLETED YEARS]<br>आयु पूर्ण वर्षों में लिखें.	Age in completed years<br>आयु पूर्ण वर्षों में ☐☐	
101	Have you ever attended school?<br>क्या आप कभी स्कूल गईं हैं?<br>If Yes, what is the highest level of schooling you have successfully completed?<br>यदि हाँ, तो आपने सबसे ऊँची कौन–सी कक्षा सफलतापूर्वक पूरी करी है?	Completed years of schooling<br>स्कूल में शिक्षा पूर्ण वर्षो में लिखें ☐☐<br>Never attended school……………..………95<br>स्कूल कभी नहीं गए	
**CHECK BOX NO. 1**<br>**IF Q101= LESS THAN 05 or 95 → Q102**		**IF Q101= 05 OR MORE → Q103**	
102	Can you read and write?<br>क्या आप पढ़ लिख सकती है?	Read only/सिर्फ पढ़ना………………………..…...1<br>Write only/सिर्फ लिखना………………….....…..2<br>Read & Write both/पढ़ना और लिखना दोनो…..3<br>Can't read and write/<br>पढ़ना और लिखना नहीं आता……………….… .4	
103	What is your current marital status?<br>Are you……<br>आपकी अभी वैवाहिक स्थिति क्या है?<br>क्या आप.......	Currently Married/वर्तमान में शादी–शुदा………1<br>Separated/ Divorced/छोड़ दिया/तलाकशुदा......2<br>Widow/विधवा……………………………..…....3<br>Never Married/शादी नहीं हुई……………….…4	 **►End**<br>**►End**<br>**►End**
104	How old were you at the time of your marriage?<br>आपकी शादी के समय आपकी आयु क्या थी?	Age in completed years<br>आयु पूर्ण वर्षो में लिखे ☐☐	
105	How old were you when you started staying with your husband? जब आप अपने पति के साथ रहने लगी उस समय आपकी क्या आयु थी?	Age in completed years<br>आयु पूर्ण वर्षो में लिखे ☐☐	
106	What is the religion you follow?<br>आप किस घर्म के है?	Hindu/हिन्दु……………….......……….……......1<br>Muslim/मुस्लिम……………...…….…………..2<br>Christian/ईसाई……………..…………….…......3<br>Sarna/सरना…...………………….....….......4<br>Others (specify)__________________________5<br>अन्य (स्पष्ट करें)	
107	Do you belong to scheduled caste, scheduled tribe or other backward class or general caste?<br>क्या आप अनुसूचित जाति या अनुसूचित जन–जाति या अन्य पिछड़े वर्ग या सामान्य वर्ग से हैं?<br>**(INS: If respondent doesn't know specify the caste)**<br>**(निर्देशः यदि उत्तरदाता अपनी ग्रुप नहीं बता सकता है, तो जाति का नाम लिखें)**	SC/एस सी………………………………….......1<br>ST/एस टी…………………………....………...2<br>OBC/ओबीसी…...………...………….……......3<br>General/सामान्य……....……………………….4<br>Caste Name___________________________<br>जाति का नाम	

108	Now I would like to know about your family composition that is whether you are living in a nuclear, or extended or joint family.<br>अब मे आपके परिवार के संबध में जानना चाहूँगी कि आपका एकल परिवार है या संयुक्त<br><br>Please tell me the names of all household members one by one and their relationship to the head of the household. ***कृप्या आप परिवार के सारे सदस्यों का एक–एक कर नाम बताए तथा घर के मुखिया से उनके संबध भी बताऐं।***	Nuclear Family/एकल परिवार .... 1<br>Extended Family/विस्तारित परिवार .... 2<br>Joint Family/संयुक्त परिवार .... 3<br><br>**I**NS: NOTE DOWN THE NAMES AND RELATIONS IN YOUR NOTE BOOK AND CIRCLE THE APPROPRIATE CODE. SEE THE FAMILY MATRIX BELOW<br>**निर्देष**: नाम और उनका संबंध अपनी पुस्तिका में लिखें एंव उचित कोड को चिन्हित करें। नीचे दिया गया परिवार मेट्रीक्स देखें	

**Type of Family** परिवार के प्रकार	**Husband** पति	**Children (Unmarried)** बच्चे (विवाहित नही है)	**Father-In-law/ Mother-in-law/ Father/Mother** ससुर / सास / माता–पिता	**Brother/Sister [Unmarried]** भाई / बहन (विवाहित नही)	**Brother/Sister's family and/or their children** भाई / बहन परिवार और उनके बच्चे
**Nuclear**					
**Extended**					
**Joint Family**					
**Joint Family**					

109	Have you done any work in the last 3 months for which you have been paid in cash, or kind or both?<br>क्या आपने पिछले तीन माह में कोई काम किया जिसके लिये आपको नकद या वस्तु या दोनो प्राप्त हुए हो?	Yes, only cash/हाँ,सिर्फ नकद .... 1<br>Yes, only kind/हाँ,सिर्फ वस्तु .... 2<br>Yes, cash & kind both/हाँ,नकद एवं वस्तु .... 3<br>No, did not work/नहीं, काम नही किया 4	→ 111
110	What kind of such work have you mainly done in the last 3 month?<br>आपने पिछले तीन माह में मुख्य रूप से किस प्रकार का काम किया?	Cultivation/Family farm/<br>खेती / परिवारिक खेती .... 1<br>Agricultural labour/कृषि श्रमिक .... 2<br>Non-agri wage Labour/गैर कृषि श्रम मजदूरी .... 3<br>Business/व्यापार .... 4<br>Salaried/वेतन भोगी .... 5<br>Other (specify) ________ 9<br>अन्य (स्पष्ट करें)	
111	What is the main occupation of your husband?<br>आपके पति का मुख्य व्यवसाय क्या है?	Cultivation/Family farm/खेती / परिवारिक खेती .... 1<br>Agricultural labour/कृषि श्रमिक .... 2<br>Non-agri wage Labour/गैर कृषि श्रम मजदूरी .... 3<br>Business/व्यापार .... 4<br>Salaried/वेतन भोगी .... 5<br>Not working/काम नहीं करते .... 6<br>Other (specify) ________ 9<br>अन्य (स्पष्ट करें)	

112	What is the main source of your household income?<br>आपके घर की मुख्य आमदनी कहाँ से आती है?	Own farm/अपनी जमीन पर खेती .... 1<br>Share Farm/बंटाई पर खेती .... 2<br>Daily Wage/ दहाड़ी मजदूरी .... 3<br>Business/निजी कारोबार .... 4<br>Salary/pension/(वेतन/पेंशन) .... 5<br>No regular work/नियमित काम नहीं .... 6<br>Other (specify)________ 9<br>अन्य (स्पष्ट करें)	
113	What is the average monthly income of your household?<br>आपके घर की औसतन मासिक आय क्या है?	Less than 1000/1000 से कम .... 1<br>1001-2000 .... 2<br>2001-3000 .... 3<br>3001-4000 .... 4<br>4001 and above/4001 से ज्यादा .... 5<br>Don't want to share/बताना नहीं चाहते .... 6<br>No Regular income/नियमित आय नहीं .... 7<br>Don't Know/पता नहीं 8	
114	Agricultural Land (in acre)<br>कृषि योग्य भूमि (एंकड में)<br>**INS: Note local units and transfer it to acre**<br>**(स्थानिय युनिट में लिखें को एकड़ में बदल कर लिखें)**	Total/कुल ______ ☐☐<br>Irrigated/सिंचित______ ☐☐<br>Non-irrigated/गैर–सिंचित ______ ☐☐	

114a	Does your household own/access any of the following?<br>क्या आपके घर में निम्नलिखित चीजें हैं/स्वामित्व में हैं?		Yes	No
		Electricity at home/घर पर बिजली	1	2
		TV (B&W)/टी.वी (ब्लैक एंड व्हाईट)	1	2
		TV (color)/टी.वी (रंगीन)	1	2
		Radio/रेडियो	1	2
		Thresher/थ्रेसर	1	2
		Tractor/ट्रेक्टर	1	2
		Water pump/पानी का पंप	1	2
		Cycle/साइकिल	1	2
		Moped/Motorcycle/मोटरसाइकिल	1	2
		Telephone/Mobile/फोन/मोबाइल	1	2
		Electric fan/बिजली का पंखा	1	2
		Sewing machine/सिलाई मशीन	1	2

EXPOSURE TO MASS MEDIA

115	Question		Exposure		Frequency of exposure			
			Yes	No	All 7 Days	5-6 days	3-4 days	Not regular
	Do you watch TV or listen to radio or read Newspaper?<br>क्या आप टी.वी देखती है या रेडियो सुनती है या समाचार पत्र पढ़ती है?<br>**Please ask one by one.**<br>**कृपया एक–एक कर पूछे**<br>If Yes, in an average week how many days out of 7 do you watch TV or listen Radio or read Newspaper?<br>**(Please tick as appropriate)**<br>यदि हाँ, तो औसतन सप्ताह के सात दिनों में आप टी.वी. कितने दिन देखती है? रेडियो कितने दिन सुनती है? अखवार कितने दिन पढ़ती है?<br>**(सभी उपयुत्त उत्तरो पर निशान लगायें)**	TV/टीवी	1	2	1	2	3	4
		Radio/ रेडियों	1	2	1	2	3	4
		Newspaper /अखवार	1	2	1	2	3	4

CMS *social*

HEALTH SEEKING BEHAVIOUR स्वास्थ्य के प्रति व्यवहार			
121	Where do you normally go to treat general ailments for your household members (children, parents etc.)?<br>आप अपने घर के सदस्यों (बच्चे, माता–पिता आदि) की सामान्य बिमारी के उपचार के लिये कहाँ जाती है?<br><br>**MULTIPLE RESPONSES POSSIBLE**<br>**(एक से अधिक उत्तर सम्भव)**<br><br>**INS: WRITE DOWN THE NAME IF CAN'T IDENTIFY THE SITE TYPE**<br>**यदि स्वास्थ्य सुविधा को पहचान न सके तो कृपया नाम लिखें**	**Public Medical Sector**<br>Govt./Urban Hospital (DH/SDH)/<br>सरकारी / शहरी अस्पताल (डीएच / एसडीएच)....A<br>CHC/Rural Hospital/PHC/<br>सामुदायिक चिकित्सा केन्द्र / ग्रामीण अस्पताल / प्राथमिक चिकित्सा केन्द्र....B<br>Sub-Centre/सब सेन्टर....C<br>Nurse / ANM/नर्स / एएनएम....D<br>Other Public Sector Health Facility (Specify)/<br>अन्य सार्वजनिक स्वास्थ्य सुविधा<br>(स्पष्ट करे)____________E<br><br>**NGO Sector**<br>NGO/Trust Hospital/Clinic/गैर–सरकारी संस्था / ट्रस्ट अस्पताल / क्लिनिक....F<br>NGO Worker/ NGO कार्यकर्ता....G<br>Sahiyaa didi/ASHA/सहिया दीदी / आशा....H<br><br>**PVT Sector**<br>Private hospital/Clinic/Nursing home/<br>प्राइवेट अस्पताल / क्लिनिक / निजी अस्पताल…I<br>Private Doctor/प्राइवेट डाक्टर....J<br>Pharmacy/Drugstore/दवा की दुकान....K<br>Vaidya/Hakim/Homeopath/<br>वैद्य / हकीम / होम्योपेथ....L<br>Traditional Healer (*Ojha*)/ओझा....M<br>Quack/unqualified village doctor/<br>नीम हकीम / अयोग्य ग्राम डाक्टर....N<br>Other Private Sector Health Facility (Specify)/<br>अन्य सार्वजनिक स्वास्थ्य सुविधा …………….. O<br>Don't Know/पता नही....X<br><br>Other (specify)____________Y<br>अन्य (स्पष्ट करे)	

No.	Question	Response	ANC	Personal
122	Where do you mostly go to consult/treat your personal health problems, more specifically for the events like:<br>आप अपने स्वास्थ्य की समास्याओं की सलाह एंव ईलाज के लिये कहाँ गई खास तौर पर निम्नलिखित परिस्थितियों में:<br><br>a. Antenatal care (ANC)<br>प्रसवपूर्व देखभाल<br><br>b. Personal health problem (white discharge, RTI, Menstruation)<br>निजी स्वास्थ्य समस्याएँ (जैसे सफेद पानी निकलना, प्रजनन मार्ग संक्रमण (RTI), माहवारी संबंधी)<br><br>**INS: ASK ABOUT THEIR LAST VISIT**<br>**निर्देशः अन्तिम बार कब गई इसके संबंध में पूछें**		**ANC**	**Personal**
		Govt./Urban Hospital (DH/SDH)/ सरकारी / शहरी अस्पताल (डीएच / एसडीएच)	11	11
		CHC/Rural Hospital/PHC/ सामुदायिक चिकित्सा केन्द्र / ग्राम अस्पताल / प्राथमिक चिकित्सा केन्द्र	12	12
		Sub-Centre/सब सेन्टर	13	13
		Other Public Sector Health Facility/ अन्य सार्वजनिक स्वास्थ्य सुविधा	14	14
		Nurse / ANM/नर्स / एएनएम	15	15
		NGO/Trust Hospital/Clinic/ गैर–सरकारी संस्था / ट्रस्ट अस्पताल / क्लिनिक	21	21
		NGO Worker/ NGO कार्यकता	22	22
		Sahiyaa didi/ASHA/ सहिया दीदी / आशा	23	23
		Dai /TBA /प्रशिक्षित / ट्रेंड दाई	24	24
		Pvt. hospital/Clinic/Nursing Home/ प्राइवेट अस्पताल / क्लिनिक / निजी अस्पताल	31	31
		Private Doctor/प्राइवेट डाक्टर	32	32
		Pharmacy/Drugstore/दवा की दुकान	33	33
		Vaidya/Hakim/Homeopath/ वैद्य / हकीम / अयोग्य ग्राम डाक्टर	34	34
		Traditional Healer (*Ojha*) /ओझा	35	35
		Quack/ unqualified village doctor/ नीम हकीम / अयोग्य ग्राम डाक्टर	36	36
		Other Private Sector Health Facility/ अन्य सार्वजनिक स्वास्थ्य सुविधा	37	37
		Don't Know / Never Tried/ पता नहीं / कोशिश नही की	98	98
		Other (specify) _________________ अन्य (स्पष्ट करें)	99	**99**
123	What is the location of the health facility (mentioned in Q122)? Is it located in this village or other village or in any nearby town?<br>स्वास्थ्य सेवा (प्रश्न 122 मे बताई गई) कहॉ पर स्थित है। यह आपके गॉव में ही है या दूसरे गॉव में है या करीब के शहर में है?	Own village/अपना गांव…………….	1	1
		Other Village/अन्य गांव…..………..	2	2
		Town/शहर…..……………………...	3	3
		Don't know/Can't say/ पता नहीं / कह नही सकते…………	8	8

Now, I would like to ask you some questions regarding the decision making in your household
अब में आपसे कुछ प्रश्न आपके परिवार के निर्णय लेने संबंधी विषय के बारे में पूछना चाहूँगी

124	Please tell me, who makes the following decisions in your household.<br>कृपया बताएं कि निम्नलिखित मे आपके परिवार में निर्णय कौन लेता है	1=Respondent/स्वंय<br>2=Husband/पति<br>3= Jointly with husband/पति के साथ मिलकर<br>4=Others in household/घर के अन्य सदस्य<br>5= Jointly with others in household/ घर के अन्य सदस्यों के साथ मिलकर				
	a. Obtaining Health care for yourself?<br>आपके आपने स्वास्थ्य देखभाल के लिए?	1	2	3	4	5
	b. Choosing any particular doctor?<br>किसी चिकित्सक को चुनने के लिए?	1	2	3	4	5
	c. What items to cook?<br>खाने में क्या बने?	1	2	3	4	5
	d. Purchasing major household items (land, Jewelry)?<br>घर की मुख्य वस्तुए (जमीन, गहने) खरीदने के संबध मे?	1	2	3	4	5

SECTION-2: REPRODUCTIVE HISTORY

Now I would like to ask you some questions about your reproductive experiences. I know that some of these questions may embarrass you or make you feel uncomfortable, but your response will be kept confidential and help us to improve issues for women like you. **अब मै आपके प्रजनन के अनुभव के बारे में आपसे कुछ प्रश्न पूछना चाहूँगी। हो सकता है कुछ प्रश्नो का जबाव देने में आपको संकोच हो लेकिन आपके द्वारा दिये गये जबाव गुप्त रखे जायेंगे और महिलाओं की समस्याओं में सुधार लाने में हमें सहायता देगें।**

Q.No	Questions and filters	Coding Categories	Skip to
201	Now I would like to ask you about all the pregnancies you have had during your life. Have you ever been pregnant? अब में आप से, आप अब तक कितनी बार गर्भवती हुई है के बारे में पूछना चाहूँगी? क्या कभी आप गर्भवती हुई है?	Yes/हाँ……………………………………….1<br>No/नहीं…..…………………………………..2	 → 212
202	How many times you became pregnant (include all pregnancies)? आप कितनी बार गर्भवती हुई। (सभी गर्भधारण को मिलाकर)?	**Total number of pregnancies** ☐☐<br>Not sure/Can't say/पता नही…………98	
203	How many live births did you have (male, female)?<br>अब तक आपने कितने बच्चों (जीवित) को जन्म दिया (लडका/लडकी)?	Male ………………… ☐<br>Female ………………… ☐<br>Total ………………… . ☐	
204	Among them how many are alive now?<br>उनमे से अभी तक कितने जीवित है?	Male ………… ☐<br>Female……… ☐	
205	Have you ever had a pregnancy that ended in a still birth?<br>क्या आपने कभी मृत बच्चे को भी जन्म दिया है?	Yes/हाँ……………………………….……….1<br>No/नहीं ……………………………..………2<br>Don't know/पता नहीं………………………8	 → 207<br>→ 207
206	How many still births did you have?<br>कितने मृत बच्चों को आपने जन्म दिया?	**Number of still births** … … … . ☐	

207	Have you ever had a pregnancy that ended in a miscarriage (spontaneous abortion)? क्या आप कभी गर्भवती हुई है जिसमें आपका स्वतः(अपने आप) गर्भपात हुआ हो?	Yes/हाँ……………………………………..1<br>No/नहीं ………………………………………2<br>Don't know/पता नहीं………………………….8	 → 209<br>→ 209
208	How many such miscarriages happened?<br>ऐसे कितने गर्भपात हुऐ है?	**Number of miscarriages** ☐	
209	Have you ever aborted a pregnancy (induced)?<br>क्या आपने कभी गर्भपात कराया है?	Yes/हाँ……………………………………..1<br>No/नहीं ………………………………………2<br>Don't know/पता नहीं………………………….8	 → 212<br>→ 212
210	How many such induced abortions happened?<br>आपने कितनी बार ऐसे गर्भपात कराया है?	**Number of induced abortions** ☐	
211	When did you have your last induced abortion?<br>आपने अन्तिम गर्भपात कब कराया?	☐☐ ☐☐<br>**m m** **y y**	
212	In addition to the details of pregnancies mentioned above, has there been any occasion during the past **three years** when your menstruation was delayed?<br>उपर बताये गये गर्भधारण के संबध में दिये विवरण के अतिरिक्त, क्या **पिछले तीन सालों** में कभी ऐसा समय आया जब आपका मासिक देर से हुआ था?	Yes/हाँ……………………………………..1<br>No/नहीं ………………………………………2<br>Don't know/पता नहीं………………………….8	 → 215<br>→ 215
213	Did you do something to resume your menstruation, as it was not in time? क्या आपने कोई उपाय किये जिससे आपका मासिक समय पर हो?	Yes/हाँ……………………………………..1<br>No/नहीं ………………………………………2<br>Don't know/पता नहीं………………………….8	 → 215<br>→ 215
214	If yes, What did you do? यदि हॉ, तो आपने क्या किया?<br>**(Open ended)**<br><br>**(INS FOR SUPERVISORS: IF YOU FEEL IT IS INDUCED ABORTION, GO BACK TO Q209 AND CORRECT IT)**<br>**(यदि आपको लगता हैं कि स्वयं कराया गया गर्भपात है, तो प्रश्न 209 में जाकर इसे सही करे।)**	______________<br>______________<br>______________	
215	Are you currently pregnant?<br>क्या आप वर्तमान में गर्भवती है?	Yes/हाँ……………………………………..1<br>No/नहीं ………………………………………2	**Check Box 2 & box 3**

CHECK BOX NO. 2
Check Q203, Q206, Q208 Q210 & Q215. Sum of all these should be equal to Q202.
Cross-check and make correction.
Check Q215=1 (Currently Pregnant)→ Check Box No: 3
Q203, Q206, Q208 Q210 और **Q215.** का जोड़ **Q202** के बराबर होना चाहिए।
यादि **Q215=1** महिला वर्तमान में गर्भवती हो तो→ **Check Box No: 3**

216	Are you or your husband currently doing something or using any method to delay or avoid getting pregnant? क्या आप या आपके पति ने गर्भ ना ठहरने या गर्भ में अन्तराल के लिये वर्तमान समय में कोई उपाय/तरीका अपनाया है?	Yes/हाँ……………………………………..1<br>No/नहीं ………………………………………2	→ 218

217	Why are you or your husband currently not using any methods to delay or avoid pregnancy?<br>आप या आपके पति ने वर्तमान समय मे गर्भ से बचने या इसमे अन्तराल रखने के लिये कोई उपाय क्यों नही अपनाये है?<br><br>INS: MULTIPLE RESPONSES POSSIBLE.<br>**निर्देशः एक से अधिक उत्तर संभव है**<br><br>**PROBE: Any other reason?**<br>**पूछें: कोई अन्य कारण?**	**Fertility related reasons**<br>Want more children/ज्यादा बच्चे चाहते है…A<br>Not having sex/infrequent sex/husband away/संबंध नहीं बना रहे/कभी–कभी संबंध/पति बाहर है.......................................B<br>Menopausal/had hysterectomy/ महावारी बंद हो जाना/बच्चादानी निकाल दी……….…C<br>Sub fecund/infecund/बाँझपन……..........…...D<br>**Opposition to use**<br>Opposed to family planning/परिवार नियोजन के विरूद्व…………..……………….……….E<br>Husband opposed/पति का विरोध……........F<br>Other family members/friends opposed/ अन्य सदस्यों/मित्रो का विरोध…………......G<br>Against religion/धर्म के विरूद्व…....….... …H<br>**Access/Knowledge**<br>Knows no method/तरीका मालूम नहीं……...I<br>Knows no source/ श्रोत मालूम नहीं...…......J<br>Hard to get method/provider too far away/ clinic hours restrictive/मिलने में कठिनाई/सेवाप्रदाता का दूर होना/क्लिनिक का समय सीमित है…………………...…….K<br>**Method related reasons**<br>Health concerns/स्वास्थ्य की चिन्ता…..……L<br>Fears side effects/नकारात्मक प्रभाव…..... ...M<br>Fears insertion/procedure/treatment/ विधि अपनाने में डर…………………………...N<br>Not effective/प्रभावशाली नहीं…..……....…O<br>Inconvenient/असुविधाजनक…..…………….P<br>Costs too much/अधिक मँहगा…….…..……Q<br>Don't like existing methods/तरीके मुझे पसंद नहीं…………………………………..……….R<br>Embarrassed/शर्म आती है…………..…...…..S<br>**Other reasons**<br>Other (specify)______________________X<br>अन्य (स्पष्ट करें)<br>Do not know/पता नही…..……..…..……… Y	**If any response Goto Check box 3**
218	Which method are you or your husband currently using?<br><br>आप या आपके पति वर्तमान मे कौन सा तरीका प्रयोग कर रहे है?	Female sterilization/महिला नसबंदी.……….A<br>Male sterilization/पुरूष नसबंदी.………..…B<br>IUD/Cu-T/आई यू डी/कापर टी…….…….C<br>Pill/गोली……………….……….....………..…D<br>Condom/Nirodh/कंडोम/निरोध……………E<br>Injectables/DMPA/ गर्भ–निरोधक इंजेक्शन/डीएमपीए..……..….F<br>Rhythm/Safe period/सुरक्षित काल पद्दित….G<br>Withdrawal/अर्धपतन यानि विदद्रावल……..H<br>Others (Specify) ____________________ X<br>अन्य (स्पष्ट करें)	

219	Since how long have you or your husband been using the method continuously? आप या आपके पति कब से इसका नियमित रूप से प्रयोग कर रहे है?	months ☐☐<br>8 years or longer……………………….96<br>8 वर्ष या ज्यादा	

CHECK BOX NO. 3

Check Q209, Q211 & Q214: Had an induced Abortion during last 3 years? (August 2007 to July 2010)

Yes =1 → Q301 No=2 → Q400 [Section 4]

क्या पिछले 3 साल के दौरान कोई गर्भपात करवाया है?

SECTION 3: ABORTION PREVELANCE AND EXPERIENCE (IN LAST 3 YEARS)

Now I would like to ask you some questions about your abortion experience. Please remember that this interview is confidential. Your answers will be very helpful in improving abortion services in communities like this. I want to talk only about the last abortion you had. **अब मै आपके गर्भपात के अनुभव के बारे में आपसे कुछ प्रश्न पूछना चाहूँगी। कृपया ध्यान दे यह साक्षात्कार गोपनीय है। आपके जबाव आपके समुदाय में गर्भपात की सेवाओं में सुधार लाने के लिए बहुत मददगार होगें। मै सिर्फ आप से आपके अंतिम गर्भपात के बारे में पूछना चाहूँगी।**

Q.No	Questions and filters	Coding Categories	Skip to
301	Why did you have this abortion (TALK ABOUT LAST ABORTION ONLY)?<br><br>आपने यह गर्भपात क्यों करवाया?<br>(सिर्फ आखरी गर्भपात के विषय में बात करे)<br><br>**Any other reason?**<br>**अन्य कोई कारण?**<br><br>**[MULTIPLE RESPONSES POSSIBLE]**<br>**एक से अधिक उत्तर संभव है**	Did not want another child then/ last child was too young/और बच्चा नहीं चाहते/अखिरी बच्चा बहुत छोटा है…….. A<br>Did not want a child at all/ have enough/बच्चा नहीं चाहते/काफी है……………………..…..... B<br>Contraceptive Failed/गर्भ–निरोधक का फेल होना………………………………………… C<br>Husband did not want the child/पति बच्चा नहीं चाहते थे……………………………….………... D<br>Mother-in-law did not want the child/सास बच्चा नहीं चाहती थी…………………….………. E<br>Could not afford another child/poverty/और बच्चे का पालन–पोषण नहीं कर सकते/गरीबी…..….. F<br>Pregnancy was a result of rape/गर्भधारण बलात्कार द्वारा हुआ……………..……………… G<br>Health problems/स्वास्थ्य संबंधी समस्या…..…….. H<br>Fetus had congenital defects/भ्रूण में पैदायशी दोष…………………………………………….. I<br>Fetus was female/भ्रूण का लड़की होना…..……. J<br>Fetus was male/भ्रूण का लड़का होना………… K<br>Other (specify)__________________________<br>अन्य (स्पष्ट करें) X	
302	Who all were involved in taking decision of your last abortion?<br>आपके आखिरी गर्भपात से संबधित निर्णय लेने में कौन कौन सम्मिलित थे?<br>**Anyone else?**<br>**अन्य कोई?**<br>**[MULTIPLE RESPONSES POSSIBLE]**<br>**(एक से अधिक उत्तर संभव है)**	Myself/स्वय…..……………………………..….A<br>Husband /पति….……………………………………B<br>Parents-in-law/सास–ससुर…...……..………………....C<br>Parents/माता–पिता…..……………………………..….D<br>Other relatives/अन्य रिश्तेदार…..………….…………..E<br>Other (specify)___________________________X<br>अन्य (स्पष्ट करें)	

<table>
<tr><td>302A</td><td>Who was the main decision maker?
मुख्य रूप से इसका निर्णय किसने लिया था?

[SINGLE RESPONSE ONLY]
(एक ही उत्तर)</td><td>Myself/स्वंय……………………………………………1
Husband/पति …………………………………………2
Mother-in-law/सास ……………………..…………3
Father-in-law/ससुर…...……………………………4
Parents/माता–पिता……………..……………………...5
Other relatives/अन्य रिश्तेदार ………………………...6
Other(specify) ________________________9
अन्य (स्पष्ट करें)</td><td>1 → 303</td></tr>
<tr><td>302B</td><td>Did you have agreement in this decision?
क्या आप इस गर्भपात के लिए सहमत थी?</td><td>Yes/हाँ…...……………………………………………1
No/नहीं……………………………………………..2
Other (specify) ________________________9
अन्य (स्पष्ट करें)</td><td></td></tr>
<tr><td>303</td><td>Where did you perform this (last) abortion? I mean at home or health facility/clinic or both at home and health facility?
आपने अंतिम गर्भपात कहाँ करवाया?अर्थात् क्या आपने गर्भपात घर मे <u>या</u> क्लिनिक/अस्पताल मे कराया <u>या</u> घर मे और क्लिनिक/अस्पताल दोनो जगह कराया?</td><td>At Home/घर मे……………………………...…...…...1
At clinic/hospital/क्लिनिक/अस्पताल मे........................2
Both at home and clinic/घर और क्लिनिक दोनो में…...3</td><td>1 → 304
2 → 308
3 → 303A</td></tr>
<tr><td>303A</td><td>You just mentioned that you performed abortion both at home and clinic; can you please explain this process in little detail? Did you first attempt performing abortion at home and then visited a clinic? Or, you visited a clinic (doctor, chemist) first and performed abortion at home.

NOTE DOWN THE STEPS (क्रम लिखें)

अभी आपने बताया किं आपने गर्भपात घर मे और क्लिनिक/अस्पताल दोनो जगह कराया। क्या आप इस बारे मे कुछ विस्तार मे बता सकती है? क्या पहले आपने गर्भपात घर में कराने की कोशिश की, फिर क्लिनिक गई थी या पहले आप क्लिनिक गई और बाद में घर पर गर्भपात कराया?</td><td colspan="2"><table><tr><th>Steps</th><th>At home / Clinic</th></tr><tr><td>1st</td><td></td></tr><tr><td>2nd</td><td></td></tr><tr><td>3rd</td><td></td></tr><tr><td>4th</td><td></td></tr></table></td></tr>
<tr><td>304</td><td>If at home, who assisted you to perform your last abortion?

यदि अंतिम गर्भपात घर पर कराया, तो इसमें आपकी सहायता किसने की?</td><td>Mother-in-Law/सास……………………………….A
Sister/बहन……………………………………………B
Sister-in-Law/ननंद…………………………………C
Mother/माँ……………………………………………..D
Friend/दोस्त……………………………………………...E
Husband /पति……………..…………………...……F
Any other(specify) ______________________X
कोई अन्य (स्पष्ट करें)</td><td></td></tr>
</table>

305	You just mentioned that you (your huband/relative/friend) tried to perform the abortion.<br><br>जैसा कि आपने कहा कि आप/आपके पति/रिश्तेदार/आपके मित्र ने आपके गर्भपात की कोशिश की<br><br>What did you (Or that person) do?<br>आपने या उस व्यक्ति ने यह कैसे किया?	Took home made concoction (Kahva, papaya, chilli) घरेलू विधि से (काहवा/पपीता/मिर्च)……………….....1<br>Took goli/tablets/गोलियाँ लीं………………...…......2<br>Ayurvedic remedies/आयुर्वेदिक दवाई ली…………......3<br>Inserted herbs/stick/जड़ी–बूटी को अंदर डाला………..4<br>Inserted instruments/कोई उपकरण अंदर डालकर…….5<br>External massage/बाहरी मालिश…………………….…6<br>Any other (Specify)__________________________ 7<br>कोई अन्य (उल्लेख करें)<br>Don't know/can't say/पता नही/कह नही सकते…..…8	
306	Did this action enable you to terminate your pregnancy completely?<br>क्या उस विधि को अपनाने से आपका पूर्ण गर्भपात हो पाया?	Yes/ हाँ……………………….………………..…1<br>No, it was incomplete/नहीं, अधूरा रह गया……..…....2<br>No, nothing has happened/नहीं,कुछ भी नहीं हुआ........3<br>I am not sure/ मुझे ठीक से नहीं पता………………....4<br>Other (specify)______________________________9<br>कोई अन्य (उल्लेख करें)	
307	Did you experience any complication after this self tried abortion?<br>स्वयं गर्भपात करने से क्या आपको कुछ जटिलताओं/समस्याओं का सामना करना पड़ा?	Yes/हाँ…………………………………………..……1<br>No/नहीं…………………………….…………….2<br><br>Other (specify)______________________________9<br>अन्य (स्पष्ट करें)	→ **308**
307A	What kind of abortion related complication(s) did you have?<br><br>आपको गर्भपात से सम्बंधित किस–किस तरह की जटिलताएँ/समस्याएँ आयी?<br><br>**MULTIPLE RESPONSES POSSIBLE**<br>**एक से अधिक उत्तर संभव है**<br><br>**Probe: Any other problems/ complications?**<br>**पूछें: अन्य कोई समस्या?**	Immediate heavy bleeding……………………...……….A<br>तुरंत ज्यादा रक्तस्राव<br>Persistent bleeding…………………………………...….B<br>(for 2 to 4 weeks post-abortion)<br>लगातार रक्तस्राव (गर्भपात के पश्चात 2 से 4 सप्ताह तक)<br>Persistent bleeding…………………………..………….C<br>(for more than 4 weeks post-abortion)<br>लगातार रक्तस्राव (गर्भपात के पश्चात 4 सप्ताह से ज्यादा)<br>Backache/ कमरदर्द……..………………………….….D<br>Abdominal pain/cramps/ पेट दर्द…………….…..….….E<br>Nausea/vomiting/ ……..……………………….....…...F<br>Foul smelling discharge……………………………..….G<br>दुर्गंधयुक्त स्राव<br>High-grade fever……………………………..……....H<br>तेज बुखार<br>Fatiuge and weakness…………………………………I<br>थकावट और कमजोरी<br>Fainting spells……………………..................................J<br>वेहोशी जैसा लगना<br>Uterus perforation/rapture…………………………..…..K<br>बच्चेदानी मे छेद<br>Irregular menstruation……………………………..……L<br>अनियमीत महावारी<br>Infertility/sterility……………………………………….....M<br>बच्चा न होना<br>Psychological symptoms: sadness, guilt, sleep, disturbance…………………………………………………N<br>मनोवैज्ञानिक लक्षण, दुःखी, आत्मग्लानी, नीद, झिझक<br><br>Other (specify) _________________________________X	

308	Did you go or consult any doctor or health worker for your abortion? By health worker we mean doctor, nurse, dai, anganwadi workers, Sahiyya/ASHA, Chemist etc. क्या आप चिकित्सक/ स्वास्थ्यकर्त्ता के पास गर्भपात के लिए या सलाह लेने गयी थी? स्वास्थ्यकर्त्ता का मतलब चिकित्सक, नर्स, दाई, आंगनवाड़ी दीदी, सहिया/आशा प्रशिक्षित दवा विक्रेता, आदि	Yes/हाँ……………………………..……………….1<br>No/नहीं……………………………….……………..2<br>Don't know/पता नहीं……………………..…………….8	 → 338<br>→ 338
309	How many providers did you go for your abortion? For example how many doctors, nurse, Sahiyya, chemist/pharmacist, dais etc.<br>आप अपने गर्भपात के लिए कितने स्वास्थ्यप्रदाता के पास गई?	No of providers …………………….. ☐<br><br>Don't know/cant's say/पता नहीं/कह नही सकते……8	
310	How many times did you visit this/these provider/s? I mean to ask, to whom did you consult in your first visit and then next and so on?<br><br>आप इन सेवाप्रदाताओं के पास कितनी बार गयी? अर्थात् गर्भपात या सलाह के लिए आप सवप्रथम किस सेवाप्रदाता के पास गयी उसके बाद क्रमशः किन सेवाप्रदाताओ के पास गयी थी<br>**Please tell me in sequence of your visits.**<br>कृपया मुझे क्रमानुसार बताऐं आप कितने बार गई<br>**INS: IT MIGHT BE ONE PROVIDER & MANY VISITS**<br>**एक सेवा प्रदाता के पास कई बार गये हो सकते हैं।**<br>**INS: IT MIGHT BE MANY PROVIDERS MANY VISITS**<br>**कई सेवा प्रदाता के पास कई बार गये हो सकते हैं।**	(see visit table below)	

Visit/ मुलाकात #	Name (if known)	Type of Provider/s
1st Visit/ मुलाकात		
2nd Visit/ मुलाकात		
3rd Visit/ मुलाकात		
4th Visit/ मुलाकात		

310A	How did you come to know that you can go to the provider for abortion?<br><br>आपको कैसे पता चला कि सेवाप्रदाता के पास गर्भपात के लिए जा सकते हैं?	ANM suggested /ए एन एम ने सलाह दी ……………......1<br>ASHA/SAHIYA sugested/ सहिया दीदी/आशा ने सलाह दी ……………………………………………………….2<br>Anganwadi Worker (AWW) suggested/ आंगनवाड़ी कार्यकर्ता ने सलाह दी………………………………….3<br>Trained Birth Attendent (TBA) suggested/ प्रशिक्षित दाई ने सलाह दी ……………………….………………........4<br>Any other health worker suggested /अन्य स्वास्थयकर्ता ने सलाह दी ______________________________5<br>NGO Worker NGO/ के कार्यकर्ता …………………...….6<br>Husband shared/ पति ने बताया ……..……….….………7<br>Family members/Friends shared/ परिवार के सदस्य/मित्र ने बताया…………………………………..……......8<br>Wall Sign/ दिवार लेखन द्वारा………………..…...…….9<br>Street Drama/ नुक्कड़ नाटक……………….…..…......10<br>Other (Specify)/ अन्य (स्पष्ट करें)________________ 11	

Now, I would like to ask you about your experiences with the provider(s) you went to for your last abortion. You told me that you visited ___ times (see 310 number of visits). Please tell me about your experience with that/each visits, starting with the 1st visit. अब में आपसे आपके आखरी गर्भपात में सेवाप्रदाता के साथ आपके अनुभव के बारे में कुछ प्रश्न पूछना चाहूंगी, आपने बताया कि आप (310 के उत्तर को लिखें)............. के पास (310 के उत्तर को लिखें) .... बार गयी, कृपया पहली बार जब गई तब से शरू करके अपने हरेक अनुभव को बतायें।

Q. No.	Questions	Coding Categories	1st Visit ________ ________	2nd visit ________ ________	3rd visit ________ ________
311	**INS: CHECK THE NAME OR TYPE OF PROVIDER UNDER EACH VISIT** निर्देशः प्रत्येक मुलाकात के दोरान स्वास्थ्यकर्त्ता का नाम और प्रकार जॉच लें			Same as 1st →318<br>Different 3 →312	Same as 1st 1→318<br>Same as 2nd 2→318<br>Different 3→ 312
312	Who was the provider you went for the last abortion? आप अपने आखिरी गर्भपात करवाने के लिए जिनके पास गई थी वो सेवाप्रदाता कौन थे ?<br><br>**INS: ASK EACH QUESTION (312-319) BY VISIT**<br>निर्देशः प्रत्येक सवाल (312-319) मुलाकात के अनुसार पुछे<br><br>**INS: IN CASE OF ONE (ONLY) PROVIDER, DON'T REPEAT (312-319)** निर्देशः यदि एक ही सेवाप्रदाता है तो सवाल (312-319) की पुनरावत्ती न करें।	Doctor- Govt./ District hospital Khunti /डॉक्टर–सरकारी ज़िला अस्पताल खुंटी	1	1	1
		Doctor- Govt. Rural hospital Silli / डॉ–सरकारी ग्रामीण अस्पताल सिल्ली	2	2	2
		Other Govt. hospital PHC/CHC______ अन्य सरकारी अस्पताल/ सामुदायिक स्वास्थ्य केंद/प्राथमिक स्वास्थ्य केंद्र	3	3	3
		Nurse/ANM/ नर्स/ए०एन०एम	4	4	4
		Other staff-Govt.Hospital/centre/ अन्य सेवादाता–सरकारी अस्पताल	5	5	5
		Doctor-Private Clinic/Ipas Provider ______________________________ डॉक्टर–प्राईवेट/क्लीनिक Ipas प्रवाईडर	6	6	6
		Other Private Doctor /Clinic/ Nurshing Home _______________________ अन्य डॉक्टर–प्राईवेट/क्लीनिक/ नर्सिंग होम	7	7	7
		Other Staff Nursging home/ अन्य सेवादाता–नर्सिंग होम	8	8	8
		Chemist/pharmacist/medical shop/ दवा विक्रेता	9	9	9
		RMP/Jholachap/quack/आर०एम०पी	10	10	10
		Dai/ दाई	11	11	11
		AWW/ आंगनवाड़ी दीदी	12	12	12
		Other(specify)/ ___________________ अन्य (उल्लेख करें)	13	13	13
		Don't know/Can't say/ पता नहीं/कह नही सकते	88	88	88
313	What was the location of the provider? वह सेवाप्रदाता किस जगह स्थित है?<br><br>**[Write down the name of location]**<br>स्थान का नाम लिखें		1st Visit ________ ________	2nd visit ________ ________	3rd visit ________ ________
		Same village/ उसी गाँव मे	1	1	1
		Other town/अन्य शहर	2	2	2
		Other village/ अन्य गाँव	3	3	3

314	How far did you travel to reach this provider?<br>आपको सेवाप्रदाता तक पहुंचने में कितनी दूरी तय करनी पड़ी?	Distance दूरी (in K.M.)<br>**Put 00 in case of same village**<br>उसी गाँव के लिए 00 लिखें।	**1st Visit**<br>________	**2nd visit**<br>________	**3rd visit**<br>________
315	Who made the final decision to go to the provider?<br>सेवाप्रदाता के पास जाने का अन्तिम निर्णय किसका था?	Self/स्वंय	A	A	A
		Husband/पति	B	B	B
		Jointly with Family/<br>परिवार के सदस्यों के साथ	C	C	C
		Mother- In -Law/सास	D	D	D
		Mother/माँ	E	E	E
		Other (specify)/________________<br>अन्य (स्पष्ट)	X	X	X
316	Why did you choose this particular provider<br>आपने इस सेवाप्रदाता को क्यों चुना?	**1st Visit**<br>________________<br>________________	**2nd visit**<br>________________<br>________________	**3rd visit**<br>________________<br>________________	
		________________<br>________________<br>________________<br>________________<br>________________	________________<br>________________<br>________________<br>________________<br>________________	________________<br>________________<br>________________<br>________________<br>________________	
317	How did you learn of the provider?<br>इस सेवा देने वाले के बारे में आपको कैसे पता चला?<br><br>**[MULTIPLE RESPONSES POSSIBLE]**<br>**(एक से अधिक उत्तर संभव है)**	Husband/पति	A	A	A
		Family members/Friends/<br>परिवार के सदस्य/दोस्त	B	B	B
		Local provider/<br>स्थानीय सेवाप्रदाता	C	C	C
		ANM/एएनएम	D	D	D
		ASHA/SAHIYA/आशा/सहिया	E	E	E
		AWW/आगंनवाड़ी कार्यकर्ता	F	F	F
		TBA/प्रशिक्षित दाई	G	G	G
		Other Health Worker ________<br>अन्य स्वास्थ्यकर्ता	H	H	H
		NGO worker/ NGO के कार्यकर्ता	I	I	I
		Wall sign /दीवार लेखन द्वारा	J	J	J
		Street Drama/नुक्कड़ नाटक	K	K	K
		Other (specify) /____________<br>अन्य (स्पष्ट)	X	X	X
318	What month of pregnancy did you visit the provider?<br>गर्भ के कौन से महिने के दौरान आप सेवाप्रदाता के पास गयी?	**Record in month/**<br>**महिनो मे लिखे**<br><br>Don't know/Can't say/<br>पता नहीं/कह नही सकते	__________<br><br>8	__________<br><br>8	__________<br><br>8

319	What type of treatment/ consultancy did you receive at this visit?<br><br>इस मुलाकात में आपने किस प्रकार की चिकित्सा/ परामर्श प्राप्त किया?<br><br>**[MULTIPLE RESPONSES POSSIBLE]**<br>**(एक से अधिक उत्तर संभव है)**<br><br>**(INS: please note down services at this visit only)**<br>**"कृप्या उन्हीं सेवाओं को लिखें जो आपको सेवाप्रदाता से इस मुलाकात में प्राप्त हुई"**	Had internal examination/ अंदरूनी जाँच की	A	A	A
		Had pregnancy test/ गर्भ की जाँच की	B	B	B
		Discussed reasons of seeking abortion/ गर्भपात करवाने के कारणों के बारे में चर्चा की	C	C	C
		Had ultra sound/ अल्ट्रासाऊँड हुआ	D	D	D
		Had surgical abortion with general anesthesia/ ऐनेस्थीसिया के साथ औजारों के प्रयोग से गर्भपात किया	E	E	E
		Had surgical abortion without general anesthesia/ ऐनेस्थीसिया के बिना औजारों के प्रयोग से गर्भपात किया	F	F	F
		Got tablets for abortion/ गोली खाकर गर्भपात किया	G	G	G
		Injections/ सुई/इंजैक्शन	H	H	H
		Given something for applying at home/ घर पर लगाने के लिए कोई पदार्थ दिया	I	I	I
		Inserted something into the vagina/ गुप्तांग/योनि के अंदर कुछ डाला	J	J	J
		Had heavy (oil) massage/ तेल से जोर से मालिश की	K	K	K
		Had abortion by_______________ से गर्भपात करवाया	L	L	L
		Treatment for abortion related complication / गर्भपात जटिलता के लिए इलाज	M	M	M
		Could not meet any doctor/refused to see/ चिकित्सक से मिल नहीं पाए/ मिलने से मना किया	N	N	N
		Referred to another doctor/hospital/ अन्य चिकित्सक/अस्पताल के पास भेजा	O	O	O
		Other (specify)__________ अन्य (स्पष्ट)	X	X	X

**CHECK BOX NO. 4**<br>**CHECK Q319 Had abortion in this visit?**<br>**इस मुलाकात में गर्भपात कराया?**			Y:1→320<br>N:2→Next visit	Y:1→320<br>N:2→Next visit	Y:1→320<br>N:2→Next visit
320	Did the Provider tell you the name of the abortion procedure?<br>क्या सेवाप्रदाता ने आपको गर्भपात की विधि का नाम बताया था?		Y e s . . . . 1<br>N o . . . 2	Y e s . . . . 1<br>N o . . . 2	Y e s . . . . 1<br>N o . . . 2
321	Could you tell me what methods have been applied on you?<br>क्या आप बता सकते हैं कि आप पर कौन सी विधि का इस्तेमाल किया था?<br>**INS: PROBE TO IDENTIFY THE METHOD AS THE**<br>**निर्देशः तरीके की पहचान के संबंध में पूछें**	1: Any surgical method with general anasthesia<br>जनरल एनसथेसिया के साथ कोई शल्य तरीका	1	1	1
		2: Any surgical method without general anasthesia<br>बिना जनरल एनसथेसिया के साथ कोई शल्य तरीका	2	2	2
		3. Medical Abortion (MA)<br>दवाइयों द्वारा गर्भपात	3	3	3
	MA: tablets or pills which can be taken orally or can be inserted into the vagina for abortion<br>MA: गर्भपात के लिए टेबलेट या गोलियां जो कि मुंह से खाई जा सकती है या फिर योनि के अन्दर डाली जा सकती है	4. Other (specify)____________<br>अन्य (स्पष्ट)	9	9	9
**INS: PLEASE ASK FOR ALL VISITS BEFORE GOING TO CHECK BOX NO.5**			**GO TO Next Visit**	**GO TO Next Visit**	**GO TO Next Visit**

CHECK BOX NO. 5

CHECK Q321: Had medical abortion ? **Yes=1→ Q322** **No=2→ Q328**
क्या गोलियो से गर्भपात हुआ था?

322	You just mentioned that your abortion has been carried out through tablets. How did you get these tablets?<br>आपने बताया कि आपका गर्भपात टेबलेट/गोलियॉ के द्वारा हुआ। आपको ये टेबलेटस/गोलियाँ कैसे प्राप्त हुयी?<br>**Would you say…….READ THE RESPONSE**<br>**क्या आप कहेंगें.........**	Doctor provided the tablets/चिकित्सक द्वारा……..…......1<br>Doctor given prescription to buy tablets………………..2<br>चिकित्सक ने टेबलेट/गोलियॉ लेने के लिए लिखित विवरण दिया<br>Chemist provided without prescription……………..…3<br>कैमिस्ट द्वारा बिना चिकित्सक के विवरण के<br>Other(specify) ____________________________4<br>अन्य (स्पष्ट)<br>Don't Know /Can't say/ पता नही/कह नही सकते…....8	→323
322a	In total, how many times did you visit to your doctor or hospital for completing the procedure?<br>कुल मिलाकर, गर्भपात के लिए, आप कितनी बार डाक्टर के पास या अस्पताल में गई/गए?	__________________ ☐	
323	Could you remember, in total, how many tablets did you take for this abortion?<br>क्या आप याद कर सकते है कि आपने कुल मिलाकर, इस गर्भपात के लिए कितनी टेबलेट/गोलियॉ खाईं?	1 tablet/1 गोली..……………………………...………1<br>2 tablets/2 गोली ……………………..……………....2<br>3 tablets/3 गोली ……………………………………….3<br>More than 3 tablets/ 2 से ज्यादा गोली…..………...……4<br>Don't remember/याद नहीं…….……………………….8	

324	Did you take one particular type of tablet or two different types of tablets?<br>क्या आपने एक ही प्रकार की टेबलेट/गोलियॉ ली थी या दो अलग अलग प्रकार की टेबलेट/गोलियॉ ली थी?	One type of tablet/एक तरह की गोली…………………1<br>Two different types of tablet/दो अलग तरह की गोली..2<br>More than two types of tablet/दो से ज्यादा तरह की गोली…………………………………………………….3<br>Don't remember/Can't say/याद नही/कह नही सकते..8	
325	Would you be able to tell me the names of the tablets you have taken for this abortion?<br>क्या आप मुझे उन टेबलेटस/गोलियॉ के बारे में बता सकते है जो आपने इस गर्भपात के लिए खाई थी?<br><br>**MULTIPLE RESPONSE POSSIBLE**<br>(एक से अधिक उत्तर संभव)<br>**INS: PLEASE CHECK THE LIST OF MA BRANDS AVAILABLE IN THE MARKET**	Mefipreston/MT Pill……………………………..…A<br><br>Misoprostol………………………………………..…B<br><br>Other(specify)_______________________________ X<br>अन्य (स्पष्ट)<br>Don't Know /Can't say/पता नही/कह नही सकते……Y	
325a	Did you take these tablets at hospital / doctor's clinic or at home?<br><br>क्या आपने ये गोलियाँ अस्पताल में/डाक्टर के क्लिनिक में/घर मे खाई?	All at clinic/ Hospital/हर बार क्लिनिक में/अस्पतालमें..1<br>One at hospital & other at home/एक बार अस्पताल और अन्य बार घर में…………….…………………..…. 2<br>All at chemist shop हर बार दवाई की दुकान में ………3<br>One at chemist shop & other at home/<br>एक बार दवाई की दुकान में और अन्य बार घर में ……4<br>All at Home/हर बार घर में ………………………….... 5<br>Other (Specify)/ _______________________________ 9<br>/अन्य (स्पष्ट)	
326	Did you take all tablets one at a time or in sequence? If in sequence, would you be able to tell me the sequence?<br>क्या आपने सभी टेबलेटस/गोलियॉ एक ही बार मे खाई थी या क्रम से खाई थी? यदि क्रम से ली थी तो क्या आप मुझे क्रम बता सकते है?<br>**PROBE FOR THE DAY OF CONSUMPTION.**<br>**उपभोग के दिन के बारे में पूछें**	All at once/एक साथ सभी…………………...………1<br>One on the 1st day & then 2nd dose on 3rd -4th day…..….2<br>1 दिन एक गोली,दूसरे दिन दूसरी, तीसरे दिन तीसरी और चौथे दिन चौथी<br><br>Other (specify)_____ ___________________________9<br>अन्य (स्पष्ट)<br>Don't Know /Can't say/पता नहीं/कह नही सकते……8	
326A	Who explained you about how to take the tablets?<br><br>आपंको यह किसने बताया कि गोलियाँ कैसे लेनी है?	Doctor डॉक्टर ……..……………………………….….1<br>Chemist कैमिस्ट ……..……………………………..2<br>ANM /एएनएम ……………………………………….3<br>ASHA / SAHIYA आशा/सहिया ……………….……..…4<br>TBA/ प्रशिक्षित दाई ……………………………………..5<br>Other (Specify) अन्य (स्पष्ट)______________________9<br>No body explained किसी ने नहीं बताया …………………7	
327	Did your provider discuss you about the possible side effects of medical abortion?<br>क्या आपके सेवाप्रदाता ने आपको औषधियुक्त गर्भपात के दुष्परिणामों के बारे में बताया था?	Yes/हाँ…..……………………………………………1<br><br>No/नहीं………………………………………………….2	

328	Thinking of all the visits you made to the provider for this abortion, can you tell me approximately how much cost did you incur on the following events:<br>सभी मुलाकातों, को ध्यान में रखते हुए। क्या आप बता सकते है कि इस गर्भपात के लिए सेवाप्रदाता के पास जाने मे इन सभी मुलाकातों पर लगभग कितना खर्चा आया<br><br>**INS: IN CASE RESPONEDENT IS UNABLE TO PROVIDE THE COST BREAKS, PROBE HER TO PROVIDE THE TOTAL APPROXIMATE COST.**<br>**निर्देशः यदि उत्तरदाता अलग–अलग खर्चे के बारे में नही बता सकता है तो उनसे पूरे खर्चे के बारे में पूछें**	Travel /Transport यातायात में [ ][ ][ ]<br>Fees/Consultation फीस में [ ][ ][ ]<br>Abortion procedure गर्भपात करवाने में [ ][ ][ ]<br>Tablets for abortion गर्भपात की गोलिया [ ][ ][ ]<br>Medicines दवाईयों में [ ][ ][ ]<br>Any test /examination अन्य जाँच में [ ][ ][ ]<br>Other(specify):_______ अन्य उल्लेख करें [ ][ ][ ]<br>**TOTAL COST(Rs.)** [ ][ ][ ][ ]	
329	Did your provider discuss you about different / any other methods of abortion?<br>क्या सेवाप्रदाता ने आपसे गर्भपात के दूसरे तरीको के बारे में भी बातचीत की?	Yes/हाँ……………………………………………………1<br>No/नहीं…………………………………………………….2<br>Don't Know/Can't say/पता नहीं / कह नही सकते….…8	
329a	Did your husband accompany you to the various providers?<br>क्या आपके पति ने आपके साथ विभिन्न सेवाप्रदाता के पास जाने में साथ दिया।	Yes, accompanied/ हाँ, साथ दिया…..…………………1<br>No/नहीं…………………………………………………….2	→ **330**
329b	Who among these accompanied you to the various providers?<br>इनमें से कौन आपके साथ विभिन्न सेवाप्रदाताओं के पास गया।	Went alone/ अकेले जाते है……………………………..…1<br>Sister-in law/ ननद / भाभी / गोतनी…………….........…2<br>Mother-in law/ सास……………………………………..…3<br>Parents/माता–पिता………………………………...….4<br>Friends/मित्र……………………………………………..5<br>Other (specify)/ अन्य (स्पष्ट)____________________ 8	
	**POST ABORTION CONTRACEPTION गर्भपात के बाद गर्भ निरोधन**		
330	Did any provider talk to you about how to avoid getting pregnant again?<br>क्या आपका गर्भपात करने वाले ने दोबारा गर्भधारण को रोकने के बारे में बातचीत की?	Yes/हाँ……………………………………………………1<br>No/नहीं…………………………………………………….2<br>Don't know/Can't say/पता नहीं / कह नही सकते……..8	 → **331**<br>→ **331**
330a	Who was that provider?<br>वह सेवाप्रदाता कौन था / थी?	_________________________	
330b	In which visit, provider talked about post abortion contraception?<br>सेवाप्रदाता ने आपको गर्भपात के बाद गर्भनिरोधक के बारे मे कब बताया?<br><br>**MULTIPLE RESPONSES POSSIBLE**<br>**एक से अधिक उत्तर संभव है**	1st Visit/ 1st मुलाकात……………………………………1<br>2nd Visit/ 2nd मुलाकात……..…………………………...2<br>3rd Visit/ 3rd मुलाकात……………….……….….……3	

331	Did you or your husband start using any contraception after your abortion?<br>गर्भपात के बाद क्या आप या आपके पति ने किसी भी प्रकार के गर्भ निरोधक का इस्तेमाल शुरू किया?	Yes/हाँ…..……………………………………………1<br>No/नहीं………………………………………………….2	→ 333
332	Why didn't you or your husband accept any family planning method to avoid another unwanted pregnancy after your abortion?<br><br>गर्भपात के बाद अनचाहे गर्भ को रोकने के लिए आप या आपके पति ने परिवार नियोजन का कोई तरीका क्यों नही अपनाया?	Wasn't counseled about FP ………………………….…..1<br>परिवार नियोजन के बारे मे किसी ने बताया नहीं<br>Didn't know any methods ……………………………….2<br>परिवार नियोजन के किसी विधि के बारे मे पता नहीं<br>Didn't know how to use any methods ………………………..3<br>परिवार नियोजन के विधि का प्रयोग कैसे करते है पता नहीं<br>Don't want to use FP methods ……………………………….4<br>किसी विधि का प्रयोग नहीं करना चाहते है<br>Due to side effects …………………………………..……….5<br>दुष्प्रभाव के डर से<br>Husband did not want ………………………….…………….6<br>पति की इच्छा नही<br>Wanted a child ………………………………………………..7<br>और बच्चे की चाहत<br>Any other reason (specify) _________________________ 8<br>काई अन्य कारण (स्पष्ट)	334A
333	Which method(s) did you accept?<br>आप या आपके पति ने कौन सा तरीका अपनाया?<br><br>**MULTIPLE RESPONSES POSSIBLE**<br>**एक से अधिक उत्तर संभव है**<br><br>**PROBE: Any other method?**<br>**पूछें: अन्य कोई तरीका?**	Male sterilization/पुरूष नंसबदी…..……………………A<br>Female sterilization/महिला नंसबदी……..…………….B<br>Pills/गोली……………………………….……………...C<br>IUD/आई यू डी…..………………………..…………...D<br>Condom/कांडोम……………………………….……..…E<br>Injectables/इंजेक्शन…..………………………………F<br>Rhythm/Periodic method/सुरक्षित काल पद्वति…..……..G<br>Withdrawal method/अर्धपतन यानि विदड्रावल...............H	
334	Did you or your husband accept that/those methods(s) because you wanted to or because it was a pre-condition for receiving the abortion?<br>क्या आप या आपके पति ने उन तरीको को इसलिए अपनाया क्योकि आप चाहते थे या यह गर्भपात के लिए निर्धारित शर्त थीा?	Willingly/खुद चाहते थे…..……..………………..…..1<br>As condition for getting abortion services/<br>यह एक शर्त थी…………………….…………..……..……2<br>Both willingly and as a condition for getting abortion services/हम चाहते भी थे और इसकी शर्त भी थी………3<br>Others (specify)/<br>अन्य (स्पष्ट) ________________________________ 6<br>Don't know/Can't say/<br>पता नहीं / कह नहीं सकते……………………………....8	

	**SATISFACTION OF WOMEN महिला की सेवा से संतुष्टि**		Refer to 1st visit ________ ________	Refer to 2nd visit ________ ________	Refer to 3rd visit ________ ________	
334A	Do you think the provider gave you enough time to explain your health situation?<br>क्या आपको लगता है की सेवाप्रदाता ने आपको काफी समय दिया।	Yes<br>No<br>Don't know	1<br>2<br>8	1<br>2<br>8	1<br>2<br>8	
334B	Do you think there was enough privacy when you shared your problem with the provider?<br>क्या आपको लगता है की सेवाप्रदाता ने काफी गोपनीयता बरती।	Yes<br>No<br>Don't know	1<br>2<br>8	1<br>2<br>8	1<br>2<br>8	

334C	How satisfied were you with the service received from this provider? Would you say, very satisfied, satisfied, neutral, unsatisfied or very unsatisfied?<br>जो सेवा आपको प्राप्त हुई उस सेवा से आप कितना संतुष्ट है, बहुत संतुष्ट, संतुष्ट, ना संतुष्ट ना असंतुष्ट, असंतुष्ट, या बहुत असंतुष्ट।	Very Satisfied<br>Satisfied<br>Nutral<br>Unsatisfied<br>Very unsatisfied	1<br>2<br>3<br>4<br>5	1<br>2<br>3<br>4<br>5	1<br>2<br>3<br>4<br>5	

Now I would like to ask you some questions regarding any complication that you may have experienced in the last abortion. **अब मैं आपसे कुछ प्रश्न पूछना चाहूँगी जो आपके आखरी गर्भपात के दौरान आई समस्याओं के अनुभव से सम्बधित है**

335	Did you have any complication after your last abortion? आखरी गर्भपात के बाद क्या आपको कोई जटिलता/ समस्या आयी है?	Yes/हाँ……1<br>No/नहीं……2<br>Don't know/पता नही/कह नही सकते……8	 → **401**<br>→ **401**
336	What kind of abortion related complication(s) did you have?<br><br>आपको गर्भपात से सम्बधित किस–किस तरह की जटिलताएँ /समस्याएँ आयी?<br><br>**MULTIPLE RESPONSES POSSIBLE.**<br>**एक से अधिक उत्तर संभव है**<br><br>**Probe: Any other problems/ complications?**<br>**पूछें: अन्य कोई जटिलता/समस्या?**	Immediate heavy bleeding……A<br>तुरंत ज्यादा रक्तस्राव<br>Persistent bleeding (for 2 to 4 weeks post-abortion)……B<br>लगातार रक्तस्राव (गर्भपात के पश्चात 2 से 4 सप्ताह तक)<br>Persistent bleeding (for more than 4 weeks post-abortion)……C<br>लगातार रक्तस्राव (गर्भपात के पश्चात 4 सप्ताह से ज्यादा)<br>Backache/कमरदर्द……D<br>Abdominal pain/cramps/पेट दर्द……E<br>Nausea/vomiting/बेहोशी/उल्टी होना……F<br>Foul smelling discharge/दुर्गंधयुक्त स्राव……G<br>High-grade fever/तेज बुखार……H<br>Fatigue and weakness/थकावट और कमजोरी……I<br>Fainting spells/वेहोशी जैसा लगना……J<br>Uterus perforation/rapture/बच्चेदानी मे छेद……K<br>Irregular menstruation/अनियमीत महावारी……L<br>Infertility/sterility/बच्चा न होना……M<br>Psychological symptoms: sadness, guilt, sleep,<br>Disturbance/मनोवैज्ञानीक लक्षण: दुःखी, आत्मग्लानी, नीद, झिझक……N<br><br>Other (specify) ________________X<br>अन्य (स्पष्ट)	
338	Did you seek care from a health care provider for any of your complication/s?<br>क्या आपने स्वास्थ्यप्रदाता से इन जटिलताओं /समस्याओं के लिए कोई सलाह/चिकित्सा ली?	Yes/हाँ……1<br><br>No/नहीं……2	→ **339**
338A	What treatment did you take to overcome from the complication(s)?<br><br>इन जटिलताओ/समस्याओं से उभरने के लिए आपने क्या ईलाज लिया?	1 ________________<br>2 ________________<br>3 ________________	Goto<br>**Q401**

339	Were you hospitalized because of any of your complications/s?<br>क्या आपको इन जटिलताओं/ समस्यों की वजह से अस्पताल में भर्ती होना पड़ा?	Yes/हाँ……………………………………….………..…1<br>No/नहीं……………………..……………………………2	→ 401
340	Where were you hospitalized?<br>आप कहां भर्ती हुयी थी?	District hospital/जिला अस्पताल…………………….……..1<br>CHC/PHC/SC/समुदायिक चिकित्सा केन्द्र/प्राथमिक चिकित्सा केन्द्र/सब सेन्टर……………………………...2<br>Private Clinic/ Nurshing Home/प्राइवेट क्लिनिक/नर्सिग होम……………………………..……..………………3<br>Any other (specify) ____________________________ 4<br>कोई अन्य (स्पष्ट)	
341	What type of treatment did you receive for this complication?<br>इस जटिलताए/समस्या के लिए आपको किस तरह का इलाज मिला?	IV injections/ Fluids(IV इंजेक्शन/फ्लूइड)………....…A<br>Abortion Again/दुबारा गर्भपात………………………….B<br>Blood Transfusion/रक्तधान…………………………….C<br>Any other (specify) ___________________________ D<br>कोई अन्य (स्पष्ट)	

SECTION 4: KNOWLEDGE AND OPINION ON ABORTION RELATED ISSUES & TECHNIQUES

Now, I would like to ask you few questions about abortion related services that women from your community can access **अब में आपसे कुछ प्रश्न पूछना चाहूँगी जो कि गर्भपात संबंधि सेवाऐं जो आपके समुदाय की महिलाएँ प्राप्त कर सकती है के बारे में हैं।**

Q.No.	Questions and filters	Coding Categories	Skip To
401	Do you think termination (induced) of pregnancy is legal in India?<br>क्या आपको लगता है कि भारत में गर्भपात वैध है?	Yes, legal/हाँ,कानूनी………………………………..1<br>Yes, legal if woman is married/हाँ, कानूनी अगर महिला शादीशुदा है तो……….……………….…....2<br>Yes, legal with certain conditions/ हाँ,कानूनी मगर कुछ शर्तो के साथ…………………3<br>No, illegal/नहीं,गैरकानूनी……………………….……4<br>No idea / Do not know/पता नही……..……………8	4, 8 → **404**
402	In your opinion, up to what length (month or weeks) of pregnancy abortion is legal in India?<br>आपकी राय में कितने समय (महिने/हफ्ते) तक का गर्भ, गर्भपात के लिए वैध है?<br>**INS: Probe a little & if answer is in month please transfer that into weeks**<br>**निर्देशः पूछें एंव यदि उत्तर महिनों मे हो तो हफ्ते में बदले**	Up to (weeks)……………..……[ ][ ]<br>No idea / Do not know…………..…………..……..98	
402A	In your opinion, up to what length (month or weeks) of pregnancy abortion can be performed using tablets?<br>आपकी राय में कितने समय (महिने/हफ्ते) तक के गर्भ का गर्भपात गोलियो से हो सकता है?<br>**INS: Probe a little & if answer is in month please transfer that into weeks**<br>**निर्देशः पूछें एंव यदि उत्तर महिनों मे हो तो हफ्ते में बदले**	Up to (weeks)……………..…….. [ ][ ]<br>No idea / Do not know……………..…..………..98	

403 & 404

403. In your knowledge, under which of the following conditions or situation abortion is legal in India? आपकी जानकारी में भारत में गर्भपात निम्नलिखित किन हालात या परिस्थितियों में वैध है?

404. Under which of the following conditions or situations do you think a woman should be able to have an abortion? Here, we are interested to know your personal opinion.
निम्नलिखित किन परिस्थितियों/दशाओं में आप सोचती है कि एक स्त्री गर्भपात करवा सकती है? यहाँ मैं आपकी व्यक्तिगत राय जानना चाहती हूँ

[INS: READ ALL OPTIONS A LOUD] **FIRST ASK Q403 AND THEN ASK Q404 FOR ALL THE OPTIONS**
(निर्देशः सभी विकल्प पढ़कर बताएं) **(सभी विकल्पो के लिए पहले प्र० 403 फिर प्र० 404 पुछे।)**

		Q403: Knowledge on legal status कानूनी स्थिति की जानकारी			Q404: Personal opinion should be able to have abortion गर्भपात के बारे में आपका निजी विचार		
		**Legal** कानूनी	**Illegal** कानूनी नहीं	**No idea** पता नहीं	**Yes** हाँ	**No** नहीं	**No idea** पता नहीं
A	If the woman is unmarried & pregnant यदि महिला अविवाहित और गर्भवती हो	1	2	8	1	2	8
B	If the pregnancy is an accident (result of a contraceptive failure)/ यदि अप्रत्याशित गर्भ हो (गर्भ निरोधक के निष्क्रिय हो जाने की वजह से)	1	2	8	1	2	8
C	If the pregnancy is a result of rape/ यदि गर्भ बलात्कार की वजह से हो	1	2	8	1	2	8
D	If the woman's health is endangered by the pregnancy/ यदि गर्भ से महिला का स्वास्थ्य खतरे में हो	1	2	8	1	2	8
E	If there is a strong chance of serious defect in the baby/ यदि पैदा होने वाले बच्चे में काफी गंभीर त्रुटि होने की संभावना हो	1	2	8	1	2	8
F	If the woman is more than 20 weeks pregnant यदि गर्भ 20 हफ्ते से ज्यादा का हो	1	2	8	1	2	8
G	If the fetus is female/ यदि भ्रूण लड़की हो	1	2	8	1	2	8
H	If the fetus is male / यदि भ्रूण लड़का हो	1	2	8	1	2	8
I	If the woman does not want another child यदि महिला एक और बच्चा ना चाहती हो	1	2	8	1	2	8
J	If the woman cannot afford the child यदि महिला बच्चे का खर्च वहन करने मै असमर्थ हो	1	2	8	1	2	8

405	Can you tell me where do married women from your community normally go to access abortion related services?<br>क्या आप बता सकती है कि आपके समुदाय की विवाहित महिलाएं आमतौर पर गर्भपात सम्बन्धित सेवाओं को पाने के लिए कहाँ जाती है?<br><br>**MULTIPLE RESPONSE POSSIBLE**<br>**(एक से अधिक उत्तर संभव हैं)**<br><br>**WRITE DOWN THE NAME OF THE DOCTOR IN CASE YOU ARE NOT SURE ABOUT FACILITY TYPE**<br><br>**अगर सुविधा के प्रकार के बारे में नही बता सकते तो डॉक्टर का नाम लिखे।**	Govt/District hospital Khunti/<br>सरकारी अस्पताल/जिला अस्पताल खुटी……………A<br>Govt/Rural hospital Silli/सरकारी/ ग्रामीण अस्पताल सिल्ली ……….……………..……..……....B<br>PHC/RH/CHC/ समुदायिक चिकित्सा केन्द/ ग्राम अस्पताल/प्राथमिक चिकित्सा केन्द्र……………......C<br>ANM/Nurse/ए एन एम/नर्स…......………........…D<br>Doctor-Private Clinic (Ipas provider)…..................E<br>डाक्टर–प्राइवेट क्लिनिक (IPAS सेवाप्रदाता)<br>Doctor- Other Private Clinic ....F<br>डाक्टर–अन्य प्राइवेट क्लिनिक<br>Nursing Home/नर्सिंग होम….……….…….……...G<br>Chemist Shop/दवा की दुकान………..…………...H<br>Ayurved Doctor/आयुवेदिक डाक्टर…….…………..I<br>Dai/TBA/प्रशिक्षित/दाई…………………….........J<br>Other traditional Healer (Ojha)/ ओझा…………...K<br>Village practitioner (unqualified)/………………..L<br>ग्राम सेवाप्रदाता (अप्रशिक्षित)<br>Other (specify)___________________________.X<br>अन्य (स्पष्ट)<br>No idea/Do not know/पता नही……………..……Z	
**CHECK BOX NO. 6**<br>**CHECK Q209, Q211 & Q214**<br>**Had an induced abortion during last 3 years? Yes =1➔Q411 No=2➔Q406**<br>**क्या आपने पिछले तीन साल में गर्भपात कराया?**			
406	If your friend/relative needed an abortion, whom would you advise them to go?<br>यदि आपके दोस्तों/रिश्तेदारों में किसी को गर्भपात की जरूरत हो तो आप किसके पास भेजना चाहेंगी?<br><br>**MULTIPLE RESPONSE POSSIBLE**<br>**(एक से अधिक उत्तर संभव हैं)**<br><br>**WRITE DOWN THE NAME OF THE DOCTOR IN CASE YOU ARE NOT SURE ABOUT FACILITY TYPE**<br>**अगर सुविधा के प्रकार के बारे में नही बता सकते तो डॉक्टर का नाम लिखे।**<br><br>**(INS: Please keep a copy of list of Ipas Supported providers with you while interviewing)**	Govt/District hospital Khunti/<br>सरकारी अस्पताल जिला अस्पताल खुटीं…………...…A<br>Govt/Rural hospital Silli/सरकारी/ ग्रामीण अस्पताल सिल्ली ……...…….……………….….…..B<br>PHC/RH/CHC/ समुदायिक चिकित्सा केन्द्र/ ग्राम अस्पताल/प्राथमिक चिकित्सा केन्द्र..................…..C<br>ANM/Nurse/ए एन एम/नर्स…..………......…….D<br>Doctor-Private Clinic (Ipas provider).....................E<br>डाक्टर–प्राइवेट क्लिनिक<br>Doctor- Other Private Clinic ....F<br>डाक्टर–अन्य प्राइवेट क्लिनिक<br>Nursing Home/नर्सिंग होम….……………......…G<br>Chemist Shop/दवा की दुकान……………...……..H<br>Ayurved Doctor/आयुर्वेदिक डाक्टर………..…..….I<br>Dai/TBA/प्रशिक्षित/दाई……………....……...…..J<br>Other traditional Healer (Ojha)/ ओझा…………....K<br>Village practitioner (unqualified)/……….……....L<br>ग्राम सेवाप्रदाता (अप्रशिक्षित)<br>Other (specify)____________________________X<br>अन्य (स्पष्ट)<br>No idea/Do not know/पता नही……………….…Z	

407	How did you come to know that you can go to the provider for abortion?<br><br>आपको कैसे पता चला कि गर्भपात के लिए सेवाप्रदाता के पास जा सकते हैं?	ANM suggested/ ए एन एम ने सलाह दी ……..……1<br>ASHA/SAHIYA sugested/ सहिया दीदी/आशा ने सलाह दी ………………………….………..…2<br>Anganwadi Worker (AWW) suggested/ आंगनवाड़ी कार्यकर्ता ने सलाह दी……..…………………………..3<br>Trained Birth Attendent (TBA) suggested/ प्रशिक्षित दाई ने सलाह दी ………………………….…..…..…….…..4<br>Any other health worker suggested/ अन्य स्वास्थयकर्ता ने सलाह दी ……………..…………………….…….…5<br>NGO Worker NGO/ कार्यकर्ता …..……..…..……........6<br>Husband shared/ पति ने बताया ……….…..……..……7<br>Family members/Friends shared/ परिवार के सदस्य/मित्र ने बताया………………..…..….………..8<br>Wall Sign/ दिवार लेखन द्वारा………..….…………….9<br>Street Drama/ नुक्कड़ नाटक………......….…………...10<br>Other (Specify) ______________________________ 11<br>अन्य (स्पष्ट करें)	
408	In your opinion, who can provide abortion related services? Would you say any health worker, or any qualified doctor, or a qualified doctor who is trained and certified or you have no idea? आपकी राय मे गर्भपात संबधित सेवायें कौन दे सकता है? क्या आप कहेंगे कोई योग्य डॉक्टर /योग्य प्रशिक्षित एवं प्रमाणित डॉक्टर /महिला स्पेशलिस्ट / या पता नहीं	Any health worker/कोई भी स्वास्थ्य कार्यकर्ता…....1<br>Any qualified doctor/कोई भी योग्य डाक्टर…..…..2<br>Trained & Certified doctor/प्रशिक्षित और प्रमाणित डाक्टर………………………………………..….3<br>Women’s Specialist doctor (Ob-gyn)/<br>स्त्री रोग विशेषज्ञ (Ob-gyn) ……………..………4<br>No idea / Do not know/पता नही …….….………8	
409	In your knowledge, what is the nearest location where you or your neighbor can access abortion related services? आपकी जानकारी में सबसे नजदीकी जगह कौन सी है जहां आप या आपके पड़ोसी गर्भपात सम्बन्धि सेवायें प्राप्त कर सकते है?	Govt/District hospital Khunti/<br>सरकारी अस्पताल…………………..………………1<br>Govt/Rural hospital Silli/सरकारी/ग्रामीण अस्पताल सिल्ली……………………..…..……………………..2<br>PHC/RH/CHC/ सामुदायिक चिकित्सा केन्द्र/ ग्राम अस्पताल/प्राथमिक चिकित्सा केन्द्र...…................…..3<br>Other Place (specify)________________________6<br>अन्य स्थान (स्पष्ट)<br>No idea / Do not know/पता नही ………..………...8	

411. Now, I would like to talk about different methods by which abortion can be performed – the various ways in which a couple can abort an unwanted pregnancy. Could you tell me what methods have you heard of?

अब मै गर्भपात के अलग अलग तरीकों के बारे में बात करना चाहूॅंगी जिनसे गर्भपात किया जा सकता है – वे विभिन्न तरीके जिनके द्वारा एक दम्पति अपने अनचाहे गर्भ को गिरा सकते है। क्या आप बता सकती है कि आपने गर्भपात के किन तरीकों के बारे में सुना है।

INS: DO NOT READ THE LIST OR PROMPT. RECORD ANSWERS GIVEN SPONTANEOUSLY FIRST BY CODING '1'.
कृप्या लिस्ट में लिखे गयें उतरों को पढ़कर न सुनायें। उत्तरदाता को उत्तर स्वंय देने दें। स्वयं दिये गये उतरों को "1" कोड करें।

FOR METHODS NOT MENTIONED SPONTANEOUSLY ASK: (जिन विधियों के नाम उत्तरदाता न ले सके पूछियें)

Now I am going to read you a list of methods of abortion. For each method I mention, please tell me if you have ever heard of the method.
अब मैं गर्भपात के तरीकों के बारे में पड़ने जा रहा हूँ। कृप्या प्रत्येक तरीके के बारे में बताये कि क्या आपने उसके बारे में सुना है।

INS: RECORD THE ANSWER ACCORDINGLY UNDER CODE 2 "Yes on probing" or Code 3 "No, on probing"
INS: FOR EACH METHOD THAT THE RESPONDENT HAS HEARD OF (either prompted or unprompted), ASK THE APPROPRIATE FOLLOW-UP QUESTIONS BELOW. IF THE RESPONDENT HAS NEVER HEARD OF A GIVEN METHOD, DO NOT ASK THE FOLLOW-UP QUESTIONS FOR THAT METHOD.
बताने पर दिये गये उत्तर को "2" कोड दे और बताने पर नहीं दिये गए उत्तर को "3" कोड दे।
INST.- जिन विधियों के बारे में उत्तरदाता ने सुना हो (स्वयं बताये गये या आपके बताये गए) उन विधियों के बारे में आगे के प्रश्न पुछे। यदि उत्तरदाता विधि के बारे में नहीं जानते, उनसे उस विधि के बारे में आगे के प्रश्न ना पुछें।

411	**Questions and filters**<br>*What methods of abortion have you heard of?*<br>आपने गर्भपात के किन–किन तरीकों के बारे मे सुना है?	What methods of abortion have you heard of?<br>आपने गर्भपात के किन–किन तरीकों के बारे मे सुना है?	Specific knowledge about the method<br>तरीके के बारे में विस्तृत जानकारी	Where do you think it is available<br>आप क्या सोचते है कि यह कहॉं उपलब्ध है
		X1	X2	X3
A	<u>MVA</u><br><br>Abortion can be performed by a hand held machine that pulls out the fetus through suction<br>गर्भपात हाथ से इस्तेमाल करने वाली मशीन से हो सकता है जो कि भ्रूण को खिचाव के द्वारा बाहर निकालता है	1 Yes, unprompted हाँ, स्वयं बताने पर<br>2 Yes, on probing हाँ, बताये जाने पर<br>3 No, on probing नहीं, बताये जाने पर ↓<br>**Next method**	A surgical method normally done under local anesthesia<br>आमतौर पर, बेहोश करके सर्जीकल तरीके से गर्भपात<br>1 Yes हाँ<br>7 Other response अन्य कोई<br>8 Do not know जानते नहीं	1. District hospital<br>2. PHC<br>3. CHC<br>4 Private doctor /Nursing home<br>5. ANM<br>6. Chemist<br>7. Other_______________<br>8. Dk/CS<br><br>**PLEASE NOTE DOWN THE LOCATION OF FACILITY**<br>**कृप्या सेवा मिलने वाले स्थान का नाम लिखें**
B	<u>D&C</u><br><br>A doctor opens the cervix and scrapes the uterus to remove the fetus<br>डॉ गर्भाशय नली को खोलता है और भ्रूण को निकालने के लिए गर्भाशय को खरोचता है	1 Yes, unprompted हाँ, स्वयं बताने पर<br>2 Yes, on probing हाँ, बताये जाने पर<br>3 No, on probing नहीं, बताये जाने पर ↓<br>**Next method**	A surgical method normally done under general anesthesia<br>आमतौर पर, बेहोश करके सर्जीकल तरीके से गर्भपात<br>1 Yes हाँ<br>7 Other response अन्य कोई<br>8 Do not know जानते नहीं	1. District hospital<br>2. PHC<br>3. CHC<br>4 Private doctor /Nursing home<br>5. ANM<br>6. Chemist<br>7. Other_______________<br>8. Dk/CS<br><br>**PLEASE NOTE DOWN THE LOCATION OF FACILITY**<br>**कृप्या सेवा मिलने वाले स्थान का नाम लिखें**

C	Medical Abortion<br>(Abortion with pills or tablets)<br>Abortion can be done by taking two different types of tablets. The second dose should be taken after 3 days of the first dose.<br>(गोलियों द्वारा गर्भपात)<br>दो अलग तरह की गोलीया खाकर गर्भपात कराया जा सकता है। दुसरी गोली, पहली गोली के तीन दिन बाद लेनी चाहिए।	1 Yes, unprompted हाँ, स्वयं बताने पर<br>2 Yes, on probing हाँ, बताये जाने पर<br>3 No, on probing नहीं, बताये जाने पर →<br>**Next method**	A woman needs to consume tablets/ pills for complete abortion<br>पूर्ण गर्भपात के लिए महिला को गोली खाने की जरूरत होती है<br>1 Yes हाँ<br>7 Other response अन्य कोई<br>8 Do not know जानते नहीं	1. District hospital<br>2. PHC<br>3. CHC<br>4 Private doctor /Nursing home<br>5. ANM<br>6. Chemist<br>7. Other________<br>8. DK/CS<br>**PLEASE NOTE DOWN THE LOCATION OF FACILITY**<br>**कृप्या सेवा मिलने वाले स्थान का नाम लिखें**
D	Any other method<br>Please specify (it might be any method)<br>कोई अन्य तरीका–<br>यदि कोई अन्य तरीका हो, तो विवरण दें			**PLEASE NOTE DOWN THE LOCATION OF FACILITY**<br>**कृप्या सेवा मिलने वाले स्थान का नाम लिखें**

CHECK BOX NO. 7

CHECK Q411C: Mentioned Medical Abortion =1 → Q412 Not Mentioned Abortion=2 → Section 5

यदि **Q411C** मे औषधिय गर्भपात(**Medical Abortion**) है तो→ **Q412** यदि औषधिय गर्भपात नहीं है तो → **Section 5**

412	You just have mentioned about pills and tablets. How did you come to know about this method?<br>**[WRITE DOWN IN VERBATIM]**<br>आप अभी टेबलेट/गोली के संबंध में बताया। आपको इस विधि/तरीके के संबंध में जानकारी कैसे मिली?<br>**बतांए गए उत्तर को लिखे**	ANM/ए एन एम……………………………………..1<br>AWW/ ए डब्लू डब्लू…………………………………..2<br>TBA/प्रशिक्षित दाई………………………………….3<br>IPC/ Group Meeting/ आई पी सी/समूह की बैठक………………………… 4<br>Wall Sign/दीवार पर चिन्ह…………………………...5<br>Street Drama/ नुक्कड नाटक……………………….6<br>Other (specify)________________9<br>अन्य (स्पष्ट)	
413	Up to what length of pregnancy a woman can terminate her pregnancy by taking tablets?<br>RECORD IN VERBATIM<br>गर्भ के कितने समय बाद तक गोली/टेबलेट से गर्भपात किया जा सकता है?<br>**INS: Please transfer the responses into weeks**<br>**निर्देशः कृपया उत्तर को हफ्ते में बदले एवं लिखे ।**	Record Actual Response [ ][ ]<br>बताये गये उत्तर को लिखें।<br>________________<br>Don't know/पता नही ……………………………..8	
414	Would you please tell me how many tablets does a woman need to complete the abortion?<br>क्या आप बता सकते/सकती है कि गर्भपात के लिये गर्भवति महिला को कितनी गोलिया लेनी होंगी?	कितनी गोलियाँ<br>Number of tablets…………………… [ ]<br>Don't know/ No idea/पता नही ………………………8	

415	Do you know whether a woman needs to take all tablets at once or in sequence to complete the abortion?<br>क्या आप को मालूम है कि गर्भपात के लिये महिला को एक साथ सारी गोलिया खानी होती है या गोलियों को क्रम से लेना होता है?	All at once/सभी एक साथ……………………………1<br>In sequence/क्रम से……………….…….…………..2<br>No idea/Can't say/पता नही/कह नही सकते………......3	→ Sec 5<br><br>→ Sec 5
416	If in sequence, would you be able to tell me the sequence?<br>यदि क्रम में लेना होता है, तो क्या आप बता सकते है कि इसका क्रम क्या होता है?<br><br>**RECORD IN VERBATIM. PROBE FOR THE DAY OF CONSUMPTION.**<br>**जानने कि कोशिश किजिए कि गोलियाँ कब ली गई थी।**	(बताये गए उत्तर को लिखें)<br>Record Actual Response<br>___________________________<br>___________________________<br><br>Don't Know /Can't say/<br>पता नही/कह नहीं सकते……………………………..8	

SECTION 5: COMMUNICATION & COMMUNITY EXPOSURE

Q.No	Questions and filters	Coding Categories	Skip To
501	In the last one year, have you received any information on family planning?<br>पिछले एक साल में क्या आपको परिवार नियोजन पर कोई जानकारी मिली है?<br>If yes where /from whom did you get this information from?<br>यदि हाँ, तो कहाँ से/किससे आपको यह जानकारी मिली थी?<br><br>**(Multiple responses possible)**<br>**(एक से ज्यादा उत्तर संभव है)**	TV/टीवी………………………………………….A<br>Radio/रेडियो….………………………...…...B<br>Newspaper/अखवार….……………………...…C<br>Wall Sign/दीवार चिन्ह………………..…………….D<br>Poster/billboard/पोस्टर/विज्ञापन…………..............E<br>Community Club/सामूदायिक क्लब……………….....F<br>NGO worker (IPC didi)/गैर सरकारी संस्था कार्यकर्ता…………………..…….………..G<br>Village doctor (local practitioner)/ग्राम डाक्टर (स्थानीय डाक्टर)…..............................……….H<br>Chemist/केमिस्ट...I<br>ANM/Nurse/ए एन एम/नर्स………….........……J<br>Sahiya/ASHA/AWW/सहीया/आशा/ए डब्लू डब्लू…........................…………........……….K<br>Dai/TBA/प्रशिक्षित दाई……..……………………L<br>Government health facility/सरकारी स्वास्थ्य सुविधा……………………….……….............…....M<br>Private provider/प्राइवेट कार्यकर्ता…………………..N<br>Husband/पति……………………………………..O<br>Family member/ Friends/neighbors/परिवार के सदस्य/दोस्त/पडोसी……………………………....P<br><br>Other (specify)_______________Q<br>अन्य (स्पष्ट)<br>No information/पता नही…………….…………….R	 <br><br><br><br><br><br><br><br><br><br><br><br><br><br><br><br><br><br><br><br>→ 503
502	Do you remember what information you received?<br>क्या आपको याद है कि आपको क्या जानकारी मिली थी?	___________________________<br>___________________________	

503	In the last two years, do you remember seeing or hearing or attending any event promoting message or information on abortion related issues?<br>कृपया याद करके बताऐं कि पिछले दो वर्ष मे क्या आपने कोई ऐसे कार्यक्रम/मीटिंग में भाग लिया/देखा/सुना जिसमें गर्भपात संबंधित विषय पर जानकारी दी गई है।<br>**IF "NO" OR "DON'T KNOW", PROBE A BIT:**<br>**Do you recall anywhere you saw/hear any message on abortion?**<br>**यदि "नहीं" या "पता नही", तो पूछेः–**<br>**क्या आपने कहीं भी गर्भपात पर कोई संदेश सुना/देखा है?**	Yes/हाँ……………………………..…………………1<br>No/नहीं………………………………………………2<br>Yes, with little probing<br>हाँ/कुछ बताने पर ……………………..…...………3	 → Sec 6

504: In the last two years, where did you get abortion related messages? Or, Tell me if someone provided you abortion related information? पिछले दो वर्ष में, गर्भपात संबंधित जानकारी कहाँ से मिली? या बताऐं यदि किसी ने आपको गर्भपात संबंधित जानकारी दी। [NOTE DOWN SPONTANEOUS RESPONSE FIRST AND SHOW CARD FOR THOSE WHICH WAS NOT SPONTANEOUSLY RECALLED] **पहले उतरदाता द्वारा स्वंय से दिए गए जवाब को दर्ज करे उसके बाद कार्ड दिखाये।**	**Reported seeing a communication event संदेशों के माध्यमों को देखा**				505: What do you feel this event was trying to tell you? Tell one by one that you still recall. आपके अनुसार इसमें आपको क्या जानकारी दी गई है? एक–एक करके याद करके बताऐं? **[ASK THIS QUESTION IF RESPONDENT RECALLED ANY EVENT. FOR EXAMPLE 504=1 OR 2]** **(यह प्रश्न तभी पुछे, यदि उत्तरदाता किसी भी माध्यम का याद करके बताता हैं)**
	**Yes: Spontaneously** हाँ, देखा खुद से बताया	**Yes: After showing cue-cards** हाँ, कार्ड दिखाने के बाद	**No: Never seen** नही, कभी नहीं देखा	**How many times did you seen** कितनी बार देखा	
A. Group Meeting- IPC [someone came to our village & discussed in a group] (कोई हमारे गाँव में आकर समूह में लोगों को जानकारी दी)	1	2	3 → Next		Message 1 ☐☐ Message 2 ☐☐ Message 3 ☐☐ Other (specify) ____
B. Wall Sign दिवार पर लिखा हुआ	1	2	3 → Next		Message 1 ☐☐ Message 2 ☐☐ Message 3 ☐☐ Other (specify) ____
C. Street Drama नुक्कड़ नाटक	1	2	3 → Next		Message 1 ☐☐ Message 2 ☐☐ Message 3 ☐☐ Other (specify) ____
D. ANM ए एन एम	1	2	3 → Next		Message 1 ☐☐ Message 2 ☐☐ Message 3 ☐☐ Other (specify) ____
D. ASHA / SAHIYA आशा/सहिया	1	2	3 → Next		Message 1 ☐☐ Message 2 ☐☐ Message 3 ☐☐ Other (specify) ____
D. Anganwadi Worker (AWW) आंगनवाड़ी कार्यकर्ता	1	2	3 → Next		Message 1 ☐☐ Message 2 ☐☐ Message 3 ☐☐ Other (specify) ____
D. Trained Birth Attendent (TBA) प्रशिक्षित दाई	1	2	3 → Next		Message 1 ☐☐ Message 2 ☐☐ Message 3 ☐☐ Other (specify) ____
E. Other [specify]____ अन्य (स्पष्ट)	1				Message 1 ☐☐ Message 2 ☐☐ Message 3 ☐☐ Other (specify) ____

E. Other<br>[specify]___________________________<br>अन्य (स्पष्ट)	1				Other (specify) ____

505: Pre-coded Options

Abortion is legal in India ……………………………………………………….…..01

Abortion can be done by using tablets ………………………………………...02

Abortion can be done by tablets upto 7 weeks of pregnancy …...……...……..03

Abortion can be done by tablets upto 9 weeks of pregnancy ……….………..04

Abortion is legal upto 20 weeks ($4^{1/2}$months) of pregnancy ……………..….. 05

Early abortion is better and safe ……………………………………….…….. 06

Nearest PHC/CHC/Govt. hospital can provide safe abortion services ……..…07

Abortion is safe if performed by a trained provider ………………………..…08

Use of regular contraception helps prevent unwanted pregnancy …………... 09

One can request abortion in case of contraceptive failure …………………. 10

One can request abortion in case of rape ……………………………….…11

Woman can request abortion in case her health is endangered ……………… 12

One can request abortion if there is a strong chance of serious risk of physical or mental abnormality to the child if born ………………………. 13

Abortion is not legal if someone wants to terminate a female …………….…14

505: Pre-coded Options

भारत में गर्भपात वैघ है……………………………..……………. 01

गर्भपात गोलियों द्वारा कराया जा सकता है……….………….……...... 02

7 हफ्ते तक का गर्भपात गोलीयो के द्वारा कराया जा सकता है. ….……. … 03

9 हफ्ते तक का गर्भपात गोलीयो के द्वारा कराया जा सकता है ……………04

गर्भधारण के 20 सप्ताह ($4^{1/2}$ महीने) के भीतर गर्भपात कराया जा सकता है… 05

गर्भपात जल्दी कराना स्वास्थ्य के लिए अच्छा और सुरक्षित है……………. 06

सरकारी अस्पताल में सुरक्षित गर्भपात की सुविधा उपलब्ध है………........07

प्रशिक्षित स्वास्थ्यप्रदाता के द्वारा कराया गया गर्भपात सुरक्षित होता है ………08

गर्भनिरोधक का नियमित इस्तेमाल द्वारा अवांछित गर्भ से बच सकते है…….. .09

यदि गर्भनिरोधक असफल रहे तो महिला गर्भपात करा सकती है…............... 10

बलात्कार होने पर महिला गर्भपात करा सकती है ……………..…..……...11

महिला गर्भपात करा सकती है यदि गर्भवती रहना उसके स्वास्थ्य के लिए नुकसानदायक हो सकता है …..12

महिला गर्भपात करा सकती है यदि गर्भ में पल रहे बच्चे में कोई बड़ी शारिरिक विकंलागता का पता चले ………..…………………………………..13

गर्भपात द्वारा महिला भ्रूण–हत्या गैर–कानूनी है ....…………….……. 14

NOTE: INTERVIEWER PLEASE DO NOT FORGET TO KEEP CUE CARDS ALONG WITH YOU
(कृप्या कार्डो को अपने पास रखें)

CHECK BOX No. 8

CHECK Q504: Exposed with any event=1 (Q504 =1 OR 2) → Q506 ; Not Exposed =2 → Section 6

यदि Q504 मे उत्तर 1 या 2 (Exposed) → Q506 ; Not Exposed → Section 6

506	Have you ever discussed/shared these issues with someone else?<br>क्या आपने यह जानकारी/विषय पर किसी से कभी चर्चा की है?	Yes/हाँ……………………………………..……1<br>No/नहीं……..……..……………………………2	→ sec 6
507	If yes, with whom?<br>यदि हाँ, तो लिखे किससे?	________________________________	

SECTION 6: BEHAVIORAL ATTRIBUTES [COMMUNICATION CUES]

In the next couple of questions, I am interested to know your opinion on certain issues related to abortion practices in your community. I will read out some statements. Please let me know whether you 'strongly agree', 'agree somewhat' or 'neither agree or nor disagree' 'disagree somewhat' or "strongly disagree'. **आगे के कुछ प्रश्नों में मैं आपके समुदाय/बिरादरी में गर्भपात की रीतियों के संबध में आपकी राय जानना चाहूँगी/चाहूँगा। मैं आपको कुछ कथन पढकर सुनाऊॅगी। कृपया आप मुझे बताये कि आप इनसे "पूर्ण सहमत", "कुछ हद तक सहमत", या "न सहमत न असहमत", कुछ हद तक असहमत या पूर्ण असहमत है।**

INS: FEW SCALE STATEMENTS ARE IN REVERSE ORDER, PLEASE READ AND CODE CAREFULLY.

		**Strongly disagree** पुर्ण असहमत	**Disagree somewhat** कुछ हद तक असहमत	**Neither agree nor disagree** न सहमत ना असहमत	**Agree somewhat** कुछ हद तक सहमत	**Strongly Agree** पुर्ण सहमत
	**Availability/ उपलब्धता**					
A	We do not have any option to get abortion services at a facility close to our village.<br>गर्भपात कराने के लिऐ हमारे गाँव के आस पास/हमारे गाँव के करीब में सुविघा उप्लब्ध नही हैं	1	2	3	4	5
B	Abortion services are normally available at urban based private clinic/nursing home.<br>गर्भपात की सुविधा आमतौर पर शहरों में प्राइवेट क्लिनिक /नर्सिग होम में उपलब्ध हैं	1	2	3	4	5
C	We don't have any information on abortion providers / हम लोगों को गर्भपात के तरीकों एंव गर्भपात करने वाले सेवाप्रदाताओं की जानकारी नहीं हैं	1	2	3	4	5
	**Affordability/ व्यय क्षमता**					
D	Abortions usually cost more than any other treatment<br>गर्भपात में दूसरे इलाज की अपेक्षा अधिक खर्च पडता हैं	1	2	3	4	5
E	Doctors usually charge more if they use any surgical procedure / यदि चिकित्सक शल्य चिकित्सा का प्रयोग करता है तो वह अधिक पैसे मांगता है	1	2	3	4	5

		Strongly disagree पुर्ण असहमत	Disagree somewhat कुछ हद तक असहमत	Neither agree nor disagree न सहमत ना असहमत	Agree somewhat कुछ हद तक सहमत	Strongly Agree पुर्ण सहमत
	**Social Norms/ सामाजिक रीति**					
F	In this community, abortions/pregnancy among **unmarried** women are usually being treated as bad/sin./ इस समुदाय में, अविवाहित महिलाओं के गर्भ/गर्भपात को बुरा/पाप माना जाता हैं	1	2	3	4	5
G	In my community, it is not acceptable to talk about any abortion related issue. हमारे समुदाय/बिरादरी में गर्भपात से जुडें किसी भी विषय पर बात करना बुरा माना जाता हैं	1	2	3	4	5
H	In this community, people who want to terminate a pregnancy prefer to go to a known health worker (like Dai, ANM didi) rather than a medical doctor हमारे समुदाय/बिरादरी में जो गर्भपात कराना चाहते है वह चिकित्सक की अपेक्षा उस स्वास्थ्यकर्मी के पास जाना पसंद करते है जिन्हें वह जानते है (जैसे दाई, एएनएम दीदी)	1	2	3	4	5
I	Couple who want to terminate their unwanted pregnancy would like to approach a provider who respects confidentiality./ अनचाहे गर्भ का गर्भपात कराने के लिये दंपति उस सेवाप्रदाता के पास जाना पसंद करेगें जहॉ उन्हे गोपनीयता का भरोसा हो।	1	2	3	4	5
	**Social Support/ सामाजिक सहायता**					
K	I can discuss all use of contraceptives with my spouse. मैं गर्भनिरोधक के सभी उपयोग के बारे में अपने पति से बात कर सकती हूं।	1	2	3	4	5
L	If I were to go for an abortion, my husband would support me / यदि मुझे गर्भपात कराने का निर्णय लेना हो तो मेरा पति इसमे मेरा साथ देंगे।	1	2	3	4	5
M	If I were to go for an abortion, my family (say parents-in-law) would support me. यदि, हम (मै और मेरा पति) गर्भपात के लिए जाते है तो मेरे अन्य परिवार जन (जैसे माता पिता) मेरा साथ देंगें।	1	2	3	4	5
N	If I need to go for abortion, I would have to do so without telling anyone/ यदि हमें (मैं और मेरा पति) को गर्भपात कराने जाने की जरूरत पड़े तो, हमें यह बिना किसी को बताऐ कराना होगा।	1	2	3	4	5
O	If I were to go for abortion, my friends and relatives would make fun of me./ यदि हमे गर्भपात के लिए जाना पड़े तो मेरे मित्र एंव संबंधी हमारा मज़ाक बनाऐंगे।	1	2	3	4	5

		Strongly disagree पुर्ण असहमत	Disagree somewhat कुछ हद तक असहमत	Neither agree nor disagree न सहमत ना असहमत	Agree somewhat कुछ हद तक सहमत	Strongly Agree पुर्ण सहमत
P	If I were to go for abortion, doctors and health care providers would treat me rudely/ यदि हमें गर्भपात के लिए डॉ या स्वास्थ्य कार्यकर्ता के पास जाना पड़े, तो उनका व्यवहार अच्छा नहीं होगा	1	2	3	4	5
	**Self-Efficacy/ स्वंय क्षमता**					
Q	I am confident; I personally can decide when I want to have children./ मुझे अपने आप पर इतना विश्वास है कि मुझे कब बच्चे चाहिए इसका निर्णय में खुद कर सकती हूँ।	1	2	3	4	5
R	I can confidently talk to my spouse about any issues related to abortion. / मैं अपने पति के साथ गर्भपात से संबधित किसी भी विषय पर बात कर सकती हूँ।	1	2	3	4	5
S	I can convince my spouse to keep gap between two children/मै अपने पति को दो बच्चों के बीच अन्तर रखने पर सहमत कर सकती हूँ	1	2	3	4	5
T	I can confidently talk to a medical doctor about issues related to abortion / मैं विश्वास के साथ एक चिकित्सक से गर्भपात संबंधी विषयों पर बातचीत कर सकती हूँ।	1	2	3	4	5
U	I can initiate discussing issues related to abortion with my neighbors/मैं अपने पडोसियों के साथ गर्भपात से जुडे विषयों पर बात करने की पहल कर सकती हूँ।	1	2	3	4	5
V	I can help my friends and relatives to choose a trained doctor./मै अपने मित्रों सगे संबधियों को प्रशिक्षित चिकित्सक चुनने में सहायता कर सकती हूँ।	1	2	3	4	5
	**Issue Severity/ विषय की गम्भीरता**					
W	Women may suffer with severe complications if abortions are carried out by untrained providers / गर्भपात अप्रशिक्षित चिकित्सक के द्वारा कराने से महिला को जटिल समस्याओं का सामना करना पड़ सकता है।	1	2	3	4	5
X	There is a risk to a mother’s health if abortion is done at a mature stage / यदि गर्भपात 12 सप्ताह के बाद कराया जाए तो महिला के स्वास्थ्य को खतरा हो सकता हैं।	1	2	3	4	5
Y	Repeated abortions harm woman’s health/लगातार गर्भपात महिला के स्वास्थ्य को नुकसान पहुँच सकता हैं।	1	2	3	4	5

CHECK BOX NO. 9

CHECK Q209, Q211 & Q214

Had an induced abortion during last 3 years? Yes=1 ➔Q701 No=2➔Continue

पिछले 3 वर्ष में गर्भपात कराया हैं।

		Strongly disagree पुर्ण असहमत	Disagree somewhat कुछ हद तक असहमत	Neither agree nor disagree न सहमत ना असहमत	Agree somewhat कुछ हद तक सहमत	Strongly Agree पुर्ण सहमत
	**Attitude**					
Z	Abortion is immoral / गर्भपात अनैतिक है।	1	2	3	4	5
AA	It is better to deliver the child than to abort it / गर्भपात कराने की अपेक्षा बच्चे को जन्म देना बेहतर हैं	1	2	3	4	5
AB	I feel guilt when I think for abortion / मुझे पाप/अपराध का आभास होता है जब मैं गर्भपात कराने की सोचती हूँ।	1	2	3	4	5
AC	Abortion is very private issue. It is better not to disclose this issue with outsiders/गर्भपात निजी मामला है। इस मामले को दूसरो को बताना ठीक नहीं है।					
AD	Occasional abortion is better than regular contraception / निरंतर गर्भनिरोध से कभी कभार गर्भपात करा लेना अच्छा हैं।	1	2	3	4	5
	**Beliefs** मान्यताएं/विश्वास					
AE	Village practitioners are more reliable than outside doctors in terms of confidentiality./जहां तक गोपनीयता का प्रश्न है गांव के डाक्टर बाहरी डाक्टरों से बेहतर हैं।	1	2	3	4	5
AF	I will never be pregnant in future if I go for abortion / यदि मैं गर्भपात कराती हूँ तो मैं फिर कभी भी भविष्य में गर्भवती नहीं हो सकती।	1	2	3	4	5
AG	Women can not work normally immediatey after abortion / गर्भपात के बाद महिला तुंरत सामान्य रुप से काम–काज नहीं कर सकती।	1	2	3	4	5
AH	A woman can die of abortion/ महिला को गर्भपात से मृत्यु हो सकती है।	1	2	3	4	5
AI	I know woman from my community who was seriously ill from abortion related complications/ मै अपनी समुदाय में ऐसी महिला को जानता/जानती हुँ जिसकी गर्भपात कराने से हालात बहुत खराब हो गई थी।	1	2	3	4	5

SECTION 7: CLOSING STATEMENT

We have reached the end of the interview. I realize that it was quite long and want to thank you again for participating. The information you have given me will be very helpful in improving services for women. As I know some of the matters we discussed today may have been embarrassing or made you feel uncomfortable, please call Mr. Debashis Sinha on 09334196224 Ipas Ranchi Office if you would like to talk to someone about this or any other problem that may arise due to this survey. हम साक्षात्कार समाप्त कर चुके। मैं आपका साक्षात्कार में भाग लेने के लिए धन्यवाद देता हूँ। आपके द्धारा दी गयी जानकारी महिलायों को पहुँचाई जाने वाली सेवाओं में सुधार लाने के लिए बहुत सहायक सिद्ध होगीं। साक्षात्कार में जिन मुद्धों/विषयो पर बात हुई उनमें से कुछ विषयो ऐसे हो सकते है जिसपर आपको बात करने मे हिचकिचाहट हुई होगी या आपको इस पर बात करने में संकोच हुआ होगा। इस सर्वेक्षण के बाद आपको कोई जानकारी लेनी हो या इस सर्वेक्षण के कारण आप को किसी असुविधा का सामना करना पडा हो तो आप कृप्या **श्री देवाशिश सिन्हा से फोन न0 09334196224** आइपास, राँची आफिस मे सम्पर्क करें।

Q.No	Questions and filters	Coding Categories	Skip To
701	Before I depart, do you have any question about the survey or the study? मेरे जाने के पहले, क्या आपके इस अध्ययन/सर्वे के बारे में कुछ प्रश्न है?	Yes/हाँ……………………………………..……1<br>No/नहीं……..…..…………………………..…2	
	**यदि हाँ, तो क्या**	______________________<br>______________________	

THANK AND TERMINATE THE INTERVIEW

धन्यवाद, साक्षात्कार समाप्त हुआ
